# Concise Total Synthesis and Pharmacological Evaluation of Procerones A and B

**DOI:** 10.1021/acsptsci.6c00196

**Published:** 2026-08-21

**Authors:** Jasmin Janneschütz, Pia B. Gartlgruber, Phila Weissenstein, Christine Aczel, Vinzenz M. Herkner, Cornelia Shtepani, Celina Haller, Yuki Bacowsky, Fatima Aslam, Laureta Gashi, Hasier Eraña, Juan C. Fuentes-Monteverde, Sarah Abel, Stefan Simić, Judith Wackerlig-Damle, Arnold Grünweller, Christian Griesinger, Joaquín Castilla, Sophia Khom, Nina Schützenmeister

**Affiliations:** † Department of Pharmaceutical Sciences, 27258 University of Vienna, Josef-Holaubek-Platz 2, Vienna 1090, Austria; ‡ Vienna Doctoral School of Pharmaceutical, Nutritional and Sport Sciences, University of Vienna, Josef-Holaubek-Platz 2, Vienna 1090, Austria; § 73038 Center for Cooperative Research in Biosciences (CIC BioGUNE), Basque Research and Technology Alliance (BRTA), Bizkaia Technology Park, Derio 48160, Spain; ∥ ATLAS Molecular Pharma S. L., Bizkaia Technology Park, Derio 48160, Spain; ⊥ Centro de Investigación Biomédica en Red de Enfermedades Infecciosas (CIBERINFEC), Carlos III National Health Institute, C/Monforte de Lemos 3-5, Madrid 28029, Spain; # IKERBASQUE, Basque Foundation for Science, Prion Research Lab, Plaza Euskadi 5, Bilbao 48009, Spain; ¶ Department of NMR-Based Structural Biology, 28282 Max Planck Institute for Multidisciplinary Sciences, Am Fassberg 11, Göttingen 37077, Germany; ∇ Institute of Pharmaceutical Chemistry, 9377 Philipps-Universität Marburg, Marbacher Weg 6-10, Marburg 35032, Germany

**Keywords:** procerones, natural products, enantiomers, aldol condensation, davis oxidation, GABA_A_ receptor

## Abstract

Procerones are highly brominated marine natural products that were isolated in 2019 from *Polycarpa procera*. Herein, we report the first total syntheses of procerones A and B, together with their enantiomers, in only three linear steps, starting from commercially available acetophenone and benzaldehyde derivatives. The synthesis takes advantage of a chalcone core structure obtained by Aldol condensation followed by Davis oxidation to implement the chiral α-hydroxy ketone moiety without the use of heavy metals. The effects of all synthesized compounds, including the enantiopure natural products, on the major inhibitory neurotransmitter receptor in the mammalian brain (GABA_A_ receptor) were determined, and the four enantiopure procerones were additionally tested for antiprion activity. While no antiprion activity was observed for the tested compounds, one unprecedented precursor, as well as procerone B and its enantiomer, significantly enhanced GABA_A_ receptor function, suggesting that this scaffold may serve as a starting point for developing novel GABA_A_ receptor modulators.

## Graphical abstract

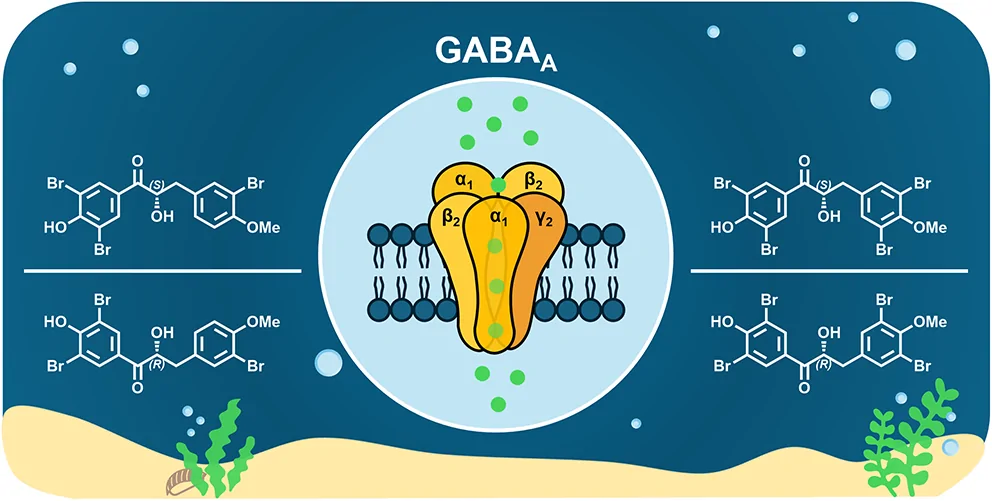

In the past decades, ascidians have emerged as a valuable source of biologically active marine natural products.^1,2^ This highlights their remarkable ability to adjust to their environment to survive.^1,2^ Procerones are a marine natural product class that was isolated from the ascidian *Polycarpa procera* in 2019.^3^ To date, two members of this novel natural product class are known, procerone A (**1a**) and B (**1b**) (Figure 1).^3^

**Figure 1:**
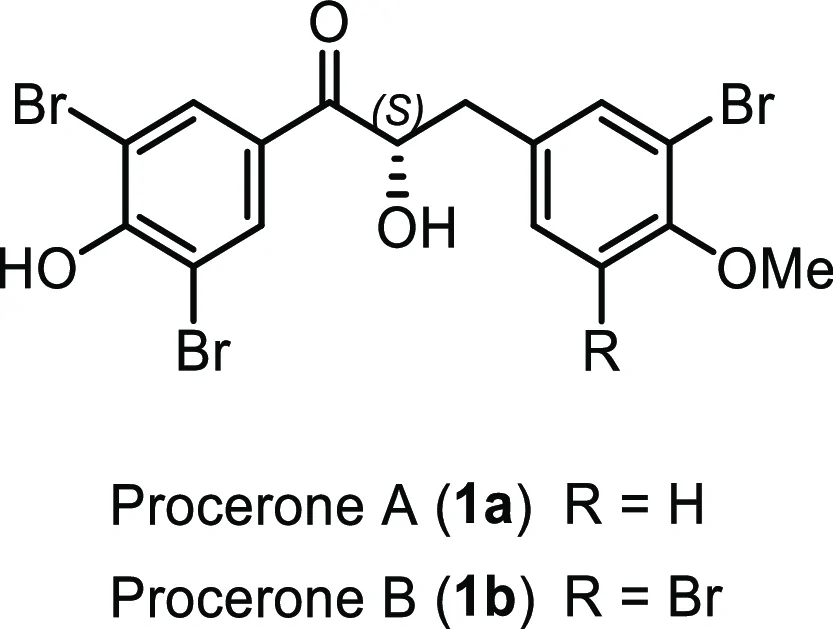
Structures of natural procerones A (**1a**) and B (**1b**).^3^

Procerones are α-hydroxy propanones, with a single stereogenic center in (*S*)*-*configuration, linked by two highly brominated phenolic rings. Biosynthetically, it was postulated that procerones are derived from amino acids, more specifically by the enzymatic condensation of two tyrosine derivatives. In a yeast prion assay, procerones A (**1a**) and B (**1b**) both showed potent antiprion activity, comparable to guanabenz,^3^ and procerone A (**1a**) further inhibited α-synuclein aggregation.^4^ These findings suggested that procerones might represent interesting scaffolds for the modulation of protein misfolding processes associated with neurodegeneration. Prion diseases are fatal neurodegenerative disorders caused by the conformational conversion of the cellular prion protein and include Creutzfeldt–Jakob disease in humans and bovine spongiform encephalopathy in cattle.^5,6^ The aggregation of certain proteins has been connected to the progression of disorders such as Parkinson’s disease.^7,8^ Procerones might become promising targets or lead structures for future drug development against neurodegenerative disorders.^3^ So far, no synthesis has been reported for this natural product class.

Structurally, procerones **1a**–**b** share similarities with chalcones **2**, a widely studied class of biologically active diaryl ketones (Figure 2).^9,10^

**Figure 2:**
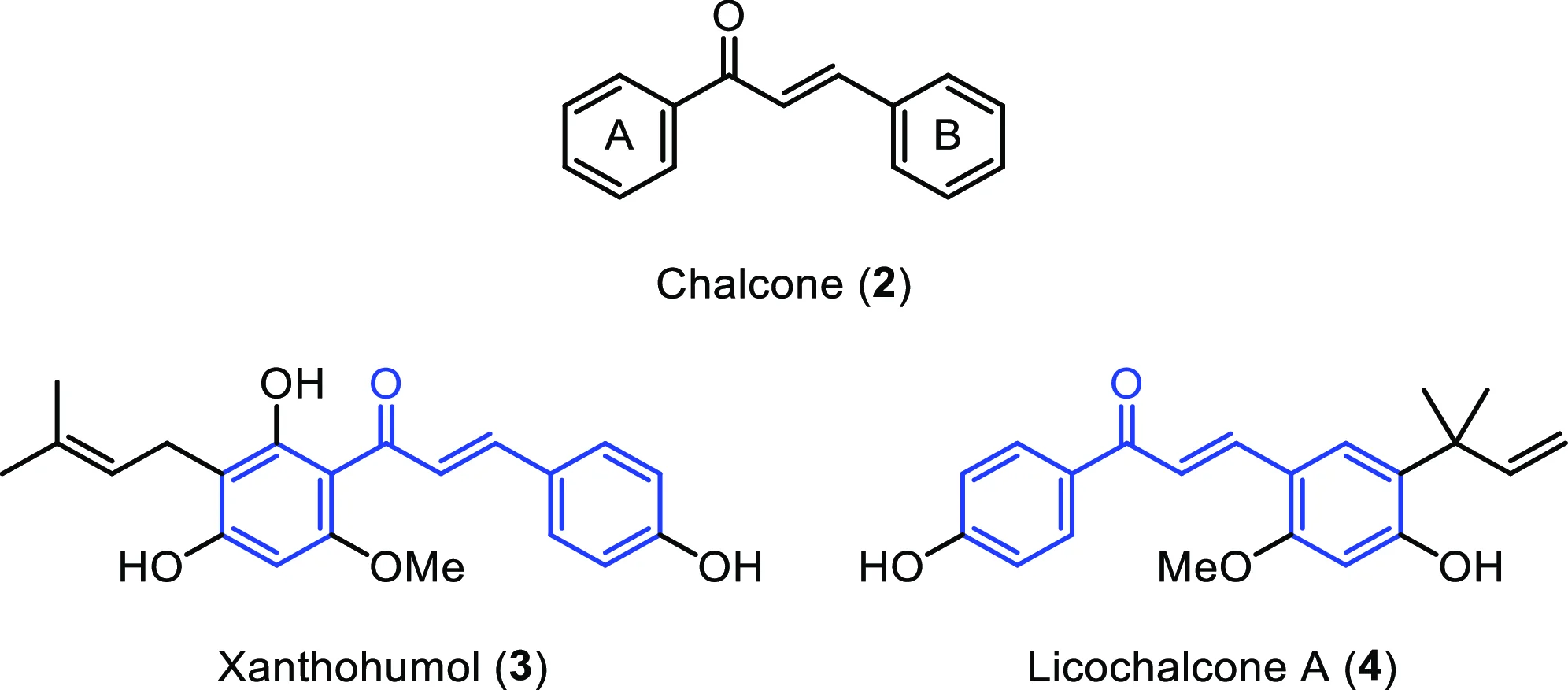
Structures of chalcone (**2**) and two representative chalcone-derived natural products, xanthohumol (**3**) and licochalcone A (**4**).

Chalcone-derived natural products, such as xanthohumol (**3**)^11^ and licochalcone A (**4**),^12^ have been synthesized via Claisen–Schmidt condensations in combination with rearrangement strategies. Although procerones **1a**–**b** contain an α-hydroxy ketone moiety rather than the α,β-unsaturated ketone characteristic of chalcones **2**, these approaches provide relevant insights for the synthesis of the procerone core structure.^9,11^

Various methods have been reported for the synthesis of α-hydroxy ketones **5** (Scheme 1A). Apart from the typical benzoin condensation,^13,14^ which involves a coupling between two aldehydes **6** and **7**, the most commonly used methods are based either on the α-hydroxylation of carbonyl compounds **8**, in which the carbonyl is converted into an enolate followed by oxidation of the double bond, or on the ketohydroxylation of alkenes **9**.^14,15^ For the asymmetric synthesis of α-hydroxy ketones, Davis reported the enantioselective α-hydroxylation using chiral *N*-sulfonyloxaziridines as oxidizing agents.^16–18^ Furthermore, asymmetric dihydroxylation of silyl enol ethers^19^ or alkenes,^20^ as well as catalytic hydrogenation^21,22^ of chiral epoxides, have also been employed for the synthesis of α-hydroxy ketones.

**Scheme 1:**
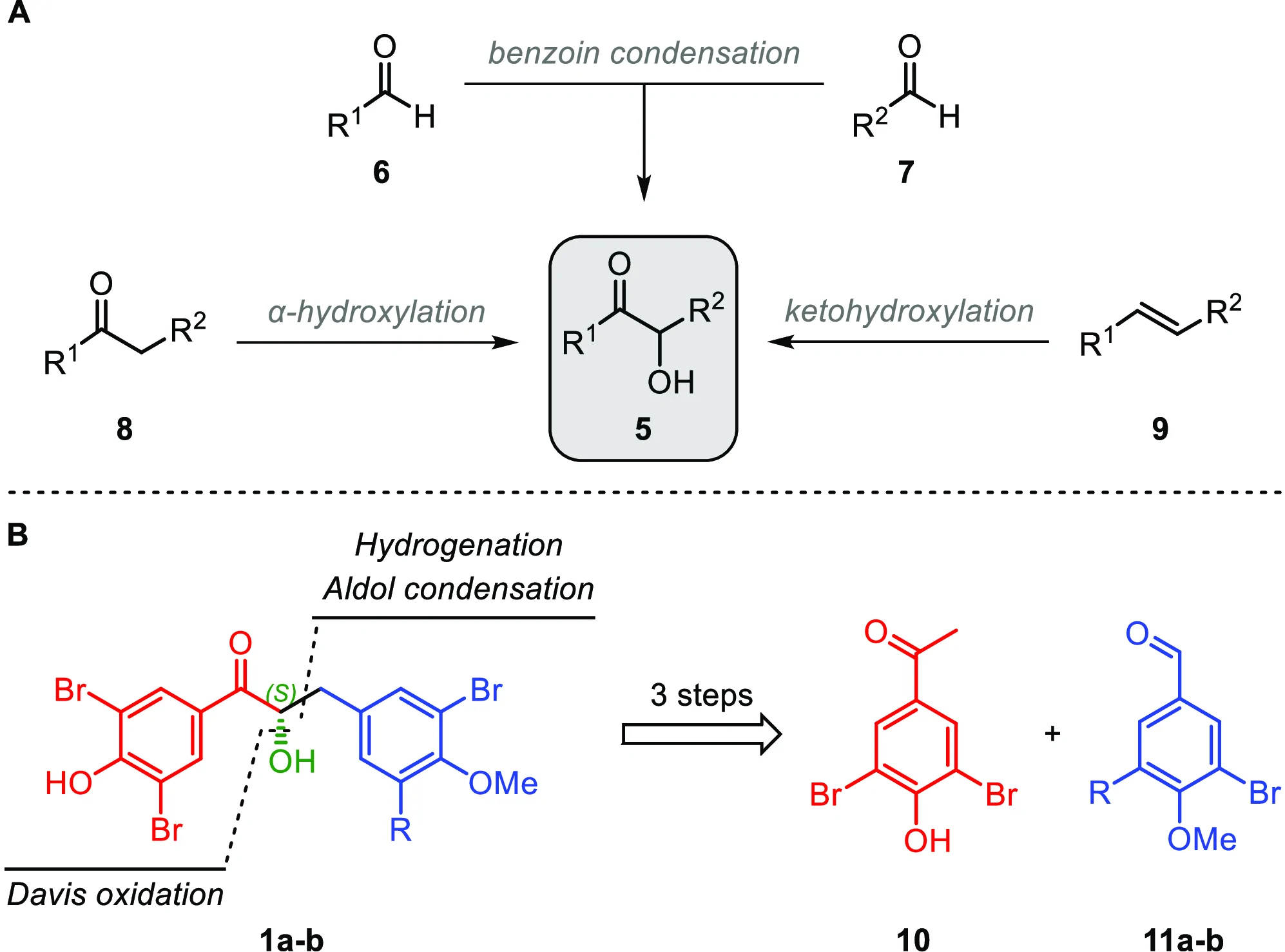
(A) Different Methods for the Synthesis of α-Hydroxy Ketones**5** and (B) Retrosynthesis of Procerones A (**1a**) and B (**1b**).

The reported methods^13–24^ have rarely been described on brominated substrates, which could increase the synthetic difficulty due to their strong destabilizing effects. The synthesis of procerones A (**1a**) and B (**1b**) employs a three-step approach starting from commercially available acetophenone and benzaldehyde derivatives **10** and **11a**–**b**, respectively (Scheme 1B). The core structure was obtained via an Aldol condensation, followed by hydrogenation of the double bond. The stereogenic center was introduced at a late stage via Davis oxidation as key step.

## Results and Discussion

The unprecedented brominated chalcones **12a**–**b** were synthesized by Aldol condensation with commercially available substituted acetophenone **10** and benzaldehyde derivatives **11a**–**b** under basic conditions and microwave irradiation (Scheme 2).^25^

**Scheme 2:**
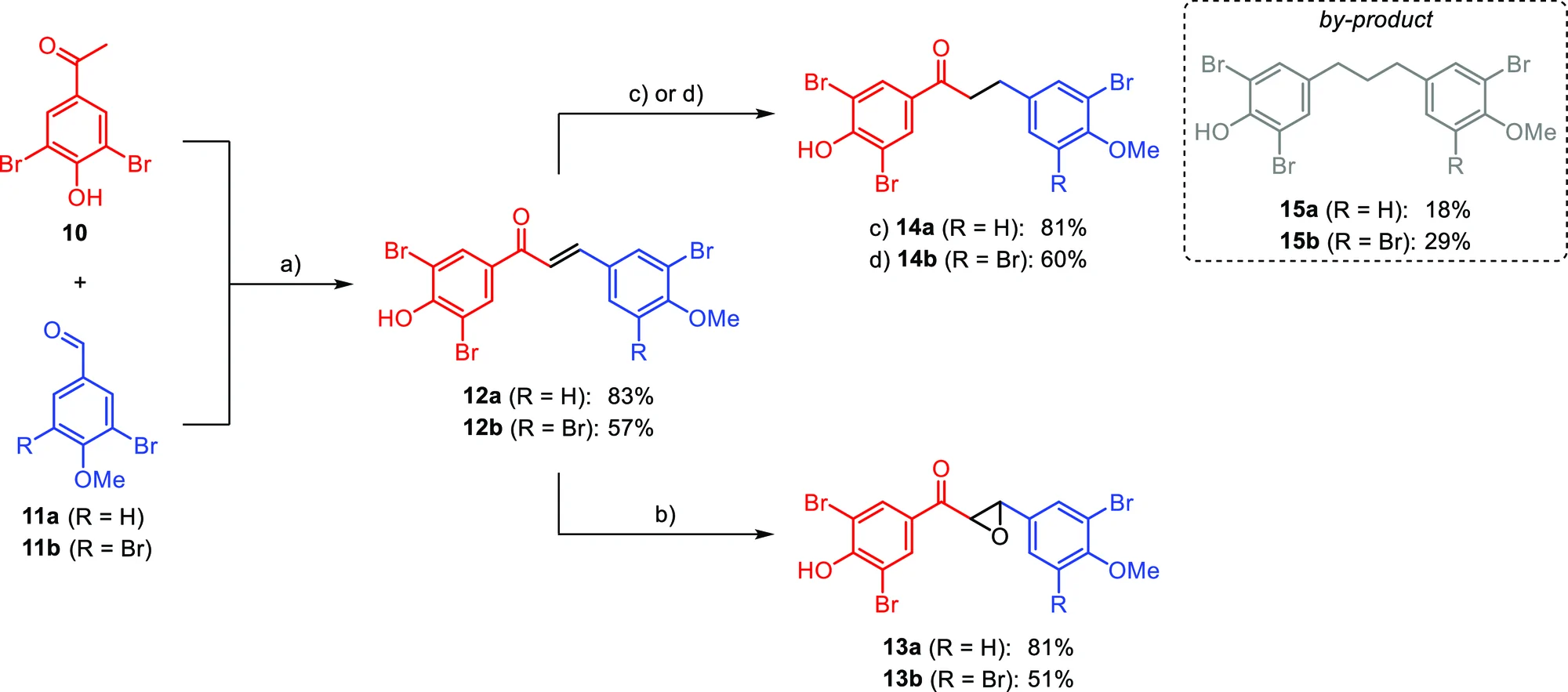
Synthesis of Epoxides**13a**–**b** and Precursors**14a**–**b** ^a^

a LiOH·H_2_O (4.0 equiv), MeOH, 55 °C (microwave irradiation), 3 h; (b) 20% aq. NaOH (4.0 equiv), 30% H_2_O_2_ (6.0 equiv), MeOH, 0 °C to r.t., 4.5 h; (c) Et_3_SiH (4.0 equiv), TFA (10 equiv), CH_2_Cl_2_, r.t., 18 h; (d) Et_3_SiH (7.0 equiv), TFA (10 equiv), CH_2_Cl_2_, 75 °C (microwave irradiation), 10 h. (TFA = trifluoroacetic acid).

The reaction conditions were optimized for the synthesis of chalcone derivative **12a** with benzaldehyde derivative **11a** as one reaction partner (Supporting Information, Table S1). Both compounds **12a**–**b** were obtained as pure *E*-isomers in yields of 83% and 57%, respectively. However, in the presence of light, the double bond can isomerize to the less stable *Z*-isomer, up to a maximum ratio of 3:1 (*E*/*Z*).

Subsequently, chalcone derivatives **12a**–**b** were epoxidized, yielding epoxides **13a**–**b** in 81% and 51% yield, respectively. Unfortunately, opening of the epoxide via selective hydrogenolysis,^21,22^ did not yield the desired products procerones A (**1a**) and B (**1b**). Alternative approaches, either by reducing agents such as BH_3_·SMe_2_,^26^ or under acidic conditions,^27^ were also unsuccessful (Supporting Information, Table S2). In addition, exposure to light caused epoxy ketones **13a**–**b** to undergo ring-opening conversions to yield the alcohol in the undesired β-position.^28^ Hence, chalcone derivatives **12a**–**b** were hydrogenated with triethylsilane in the presence of trifluoroacetic acid (TFA) (Scheme 2) to obtain **14a**–**b**. While derivative **14a** was hydrogenated at room temperature in 81% yield, olefin **12b** was hydrogenated to **14b** under microwave irradiation at 75 °C in 60% yield. In both cases, byproducts **15a**–**b** were obtained in 18% and 29% yield, respectively.

Procerones A (**1a**) and B (**1b**) were then successfully synthesized by Davis oxidation from the previously hydrogenated products **14a**–**b**, in 33% and 30% yield, respectively (Scheme 3).

**Scheme 3:**
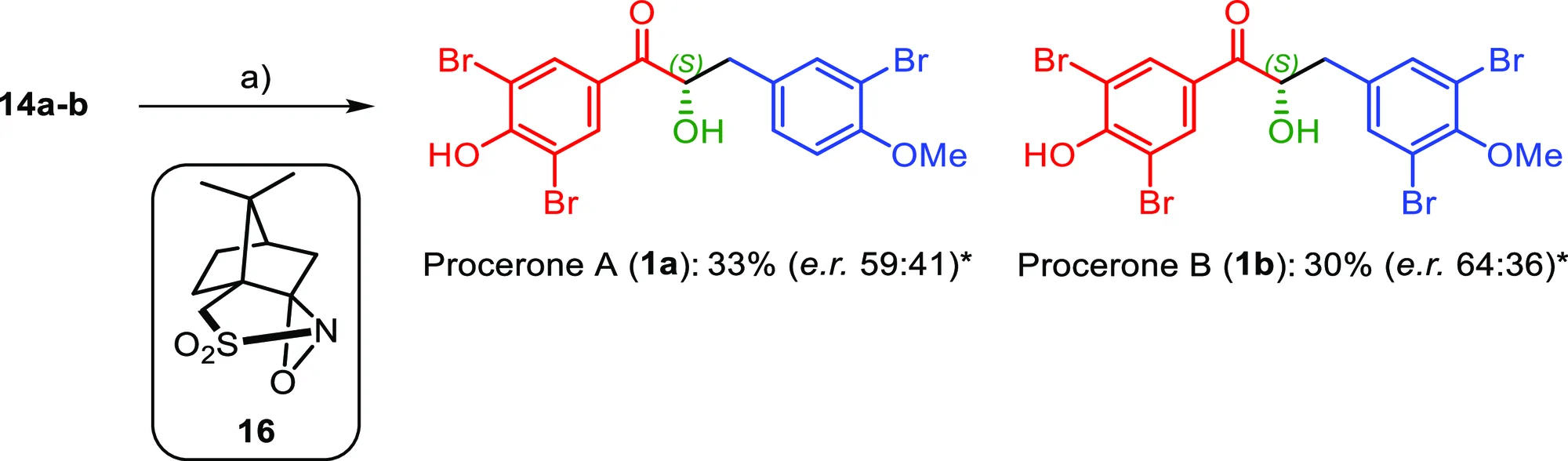
Synthesis of Procerones A (**1a**) and B (**1b**)­^a^

a NaHMDS (3.0 equiv), THF, −78 °C, 30 min, then (1*S*)-(+)-(10-camphorsulfonyl)-oxaziridine (**16**) (1.5 equiv), −78 °C, 30 min *Enantiomeric ratio (*e.r.*) determined by chiral HPLC analysis of the crude mixture.

The use of chiral camphorsulfonyl­(oxaziridine) **16** as oxidizing agent delivered procerones **1a**–**b** with moderate enantiomeric excess (*ee*). The *ee*-value of the fully brominated procerone B (**1b**) was slightly higher (27%) than that of procerone A (**1a**) (19%) (Table 1, entries 1–2). As described in the literature, the α-hydroxy ketone might not be formed until the workup step.^17^ In addition, it was shown that the enantioselectivity is directly dependent on the temperature during the workup step.^17^ Therefore, this temperature was screened using hydrogenated derivative **14a** as substrate (Table 1, entries 2–7).

**Table 1:** Screening Results of the Davis Oxidation with **14a**/**14b** (1.0 equiv) as Substrates^a^

entry	substrate	T [°C]^b^	*ee* [%]^c^	*ee* [%] after purification^c^
1	**14b**	–78	27	24^d^
2	**14a**	–78	19	8^e^
3	**14a**	–60	15	8^e^
4	**14a**	–40	5	2^e^
5	**14a**	–20	41	14^e^
6	**14a**	0	28	n.d.
7	**14a**	r.t.	21	10^e^

a All attempts were carried out with NaHMDS (3.0 equiv), THF, −78 °C, 30 min, then (1*S*)-(+)-(10-camphorsulfonyl)-oxaziridine (**16**) (1.5 equiv), −78 °C, 30 min (*ee* = enantiomeric excess, n.d. = not determined).

b Temperature at the beginning of the aqueous workup.

c Determined by chiral HPLC analysis.

d Trituration with CHCl_3_ and subsequent column chromatography.

e Column chromatography and subsequent recrystallization from MeOH.

Performing the reaction workup at −60 °C or −40 °C resulted in lower *ee*-values compared to −78 °C (entries 2–4). Interestingly, this trend was interrupted at −20 °C, where the highest *ee*-value of 41% was obtained (entry 5). At higher temperatures, the enantioselectivity decreased again (entries 6 and 7). Furthermore, the purification method significantly influenced the observed *ee*-values. Recrystallization from MeOH reduced the initial *ee* by approximately half or more, suggesting preferential crystallization of *ent*-**1a** relative to **1a**. In the case of procerone B (**1b**), recrystallization was replaced by trituration with CHCl_3_, which did not significantly affect the *ee*.

To our delight, a single-crystal X-ray diffraction (SXRD) structure of procerone A (**1a**) (CCDC 2471100) was obtained, which additionally confirmed the first successful synthesis (Figure 3).^29^

**Figure 3:**
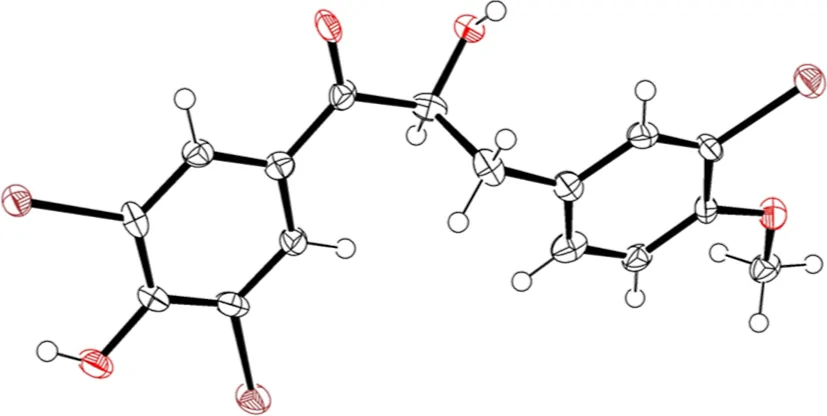
SXRD structure of procerone A (**1a**) (CCDC 2471100). Thermal ellipsoids are shown at 50% probability level. (SXRD = single-crystal X-ray diffraction).

Both enantiomers of **1a** and **1b** were separated by semipreparative high-performance liquid chromatography (HPLC) on a Chiralpak IA column (5 μm particle size; 20 mm ⌀ × 250 mm L, Daicel Inc., Tokyo, Japan), yielding the four enantiopure compounds **1a**/*ent-* **1a** and **1b**/*ent-* **1b**. The enantiomeric purity was determined by analytical chiral HPLC (see Supporting Information for more details).

The initial assignment of the absolute configurations was based on optical rotation measurements. The compounds that eluted first during the HPLC separation showed positive optical rotations, with values of +13.0° for procerone A (**1a**) and +24.0° for procerone B (**1b**). These results were consistent with the previously isolated natural products, and therefore supported the assignment as (2*S*)-enantiomers.^3^ In contrast, the previously unreported enantiomers showed corresponding negative optical rotations of −16.5° for *ent*-**1a** and −23.5° for *ent*-**1b**, and were assigned as (2*R*)-enantiomers. Our results were further supported by electronic circular dichroism (ECD) measurements (Figure 4).

**Figure 4:**
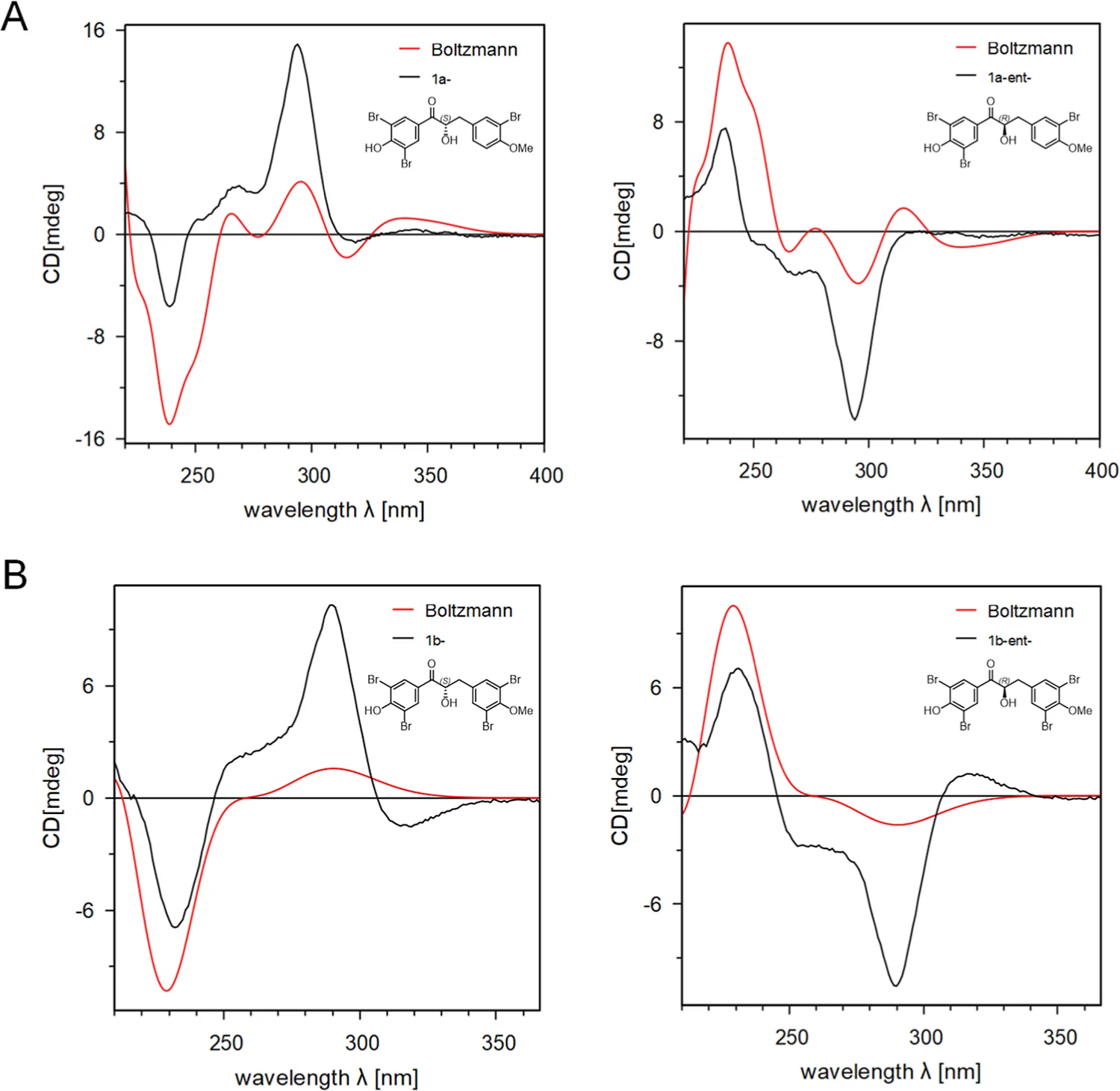
(A) ECD fitting of procerone A (**1a**) (left) and *ent*-**1a** (right). Experimental ECD appears in black, and DFT-computed Boltzmann averaged is shown in red. ECD was computed at PW6B95D3 ^30^/Def2TZVPP^31^/W06, solvent modeled by COSMO (MeOH), including the lowest 160 singlet excited states. Spectrum fitting parameters: Sigma/gamma (eV): 0.20; UV correction 20 nm; Wavelength (nm) 220–400. (B) ECD fitting of procerone B (**1b**) (left) and *ent*-**1b** (right). Experimental ECD appears in black, and DFT-computed Boltzmann averaged is shown in red, solvent modeled by COSMO (MeOH), including the lowest 260 singlet excited states. ECD was computed at CAM-B3LYP^32^/x2c-TZVPall-2c^33^/auto. Fitting parameters: Sigma/gamma (eV): 0.27; UV Correction −24 nm; Wavelength (nm) 210–366.

ECD spectra of procerones A (**1a** and *ent*-**1a**) and B (**1b** and *ent*-**1b**) were calculated at density functional theory (DFT) level and compared with the corresponding experimental spectra (see Supporting Information for more details). The calculated ECD spectra matched very well with the experimental spectra (Figure 4), confirming the absolute configurations of procerones A (**1a**) and B (**1b**) as (2*S*), and their corresponding enantiomers *ent*-**1a** and *ent*-**1b** as (2*R*).

## Experimental Section

Given the promising findings by Carroll and co-workers, we were interested in whether this novel natural product class could inhibit the propagation not only of yeast prions^3^ but also of mammalian prions. Furthermore, these results should contribute to the assessment of the proposed more general mechanism of action of these natural products.^4^

Hence, to evaluate the potential antiprion activity of procerones A (**1a**) and B (**1b**), together with their enantiomers *ent*-**1a** and *ent*-**1b**, protein misfolding shaking amplification (PMSA)^34^ propagation assays were performed using the four previously synthesized, distinct molecular entities. These compounds were tested at a concentration of 50 μm against two well-characterized recombinant prion strains: Ust02 (bank vole 109I PrP-derived)^35^ and btMI-09 (mouse L108I PrP-derived).^36^ All compounds were successfully solubilized in DMSO and maintained stable throughout the experimental period. Substrate quality controls demonstrated adequate protein content and structural integrity for both rec-Vole109I and rec-MoL108I substrates, as evidenced by total protein staining prior to inoculation (Figure 5).

**Figure 5:**
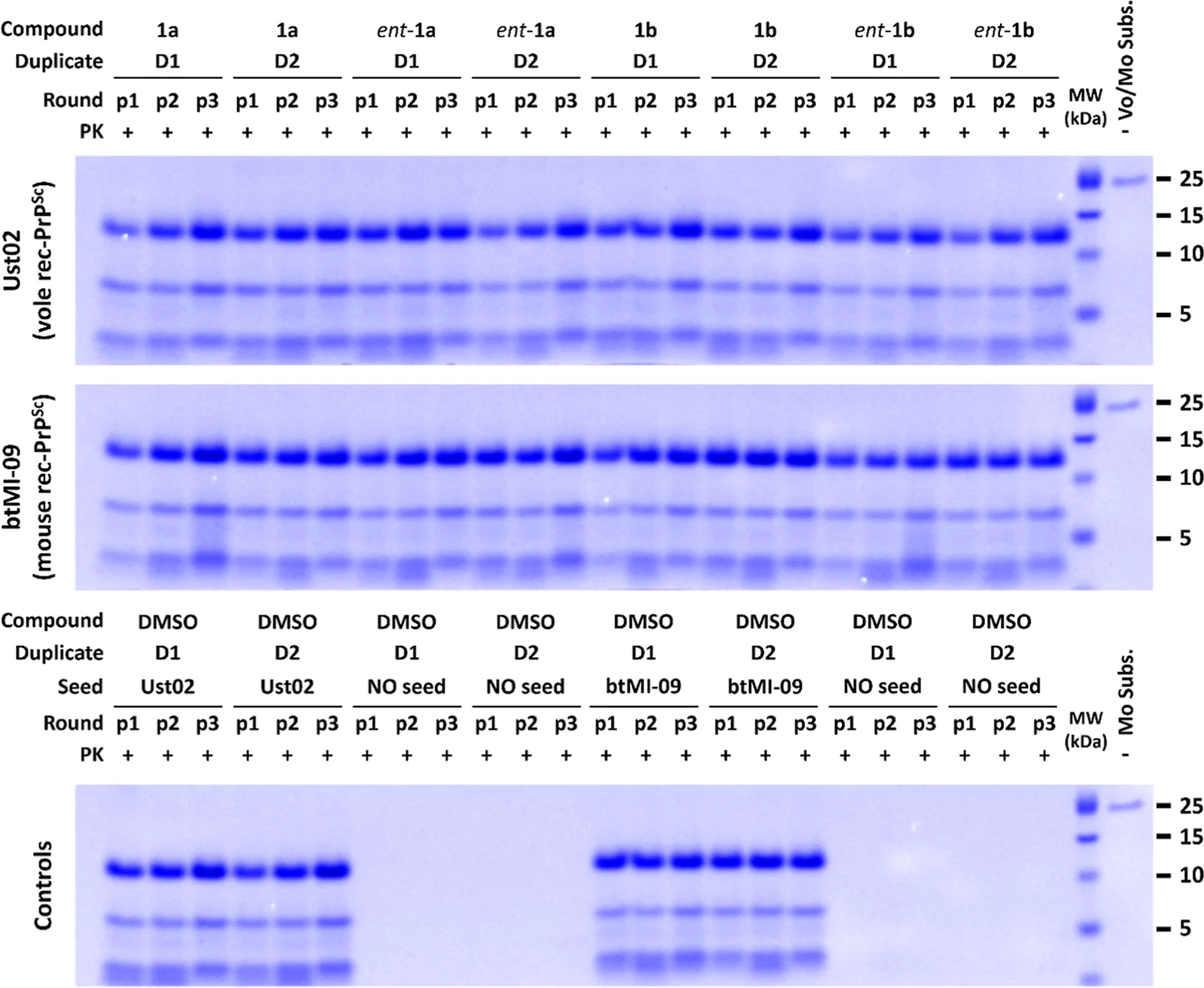
Evaluation of prion propagation in PMSA assays. PMSA assays using the four enantiopure compounds: procerone A (**1a**), *ent*-**1a**, procerone B (**1b**), and *ent*-**1b** at 50 μm concentration. **Upper panel**: Results using bank vole 109I recombinant prion protein substrate inoculated with Ust02 strain. **Lower panel**: Results using mouse L108I recombinant prion protein substrate inoculated with btMI-09 strain. Three consecutive serial passages or PMSA rounds (p1, p2, p3) performed in duplicate (D1, D2) for each compound are shown. DMSO controls represent conditions without compound. Negative controls (“No seed”) lack prion inoculum. Substrate quality was verified by protein staining (“Vo/Mo Subs”). Samples were digested with proteinase K (50 μg/mL) and analyzed by electrophoresis and total protein staining. Visible bands indicate protease-resistant forms, evidencing successful prion propagation. MW: molecular weight marker.

Following inoculation with either Ust02 or btMI-09 strains, robust prion propagation was observed across all three serial passages (R1, R2, R3) in both duplicate reactions for each compound tested. Proteinase K digestion followed by electrophoretic analysis revealed characteristic protease-resistant PrP patterns consistent with successful prion amplification. Notably, the intensity and migration patterns of PrP^res^ bands remained comparable between procerone-treated samples and DMSO controls throughout all passages, indicating no detectable inhibitory effect on prion propagation. Negative controls lacking prion seeds remained consistently negative across all passages and experimental conditions, confirming the absence of spontaneous prion formation and validating the specificity of the propagation signals observed in inoculated samples.

The propagation efficiency appeared unaffected by the presence of any of the four enantiopure compounds **1a**, *ent*-**1a**, **1b**, and *ent*-**1b**, as evidenced by the maintenance of strong PrP^res^ signals through three consecutive 1:100 dilution passages. No differences in electrophoretic mobility or band intensity were observed between compounds **1a**, *ent*-**1a**, **1b**, *ent*-**1b**, and their respective DMSO controls, suggesting that neither the core procerone structure nor the stereochemical configuration influences prion propagation under these experimental conditions.

These results indicate that procerones A (**1a**) and B (**1b**), along with their respective enantiomers *ent*-**1a** and *ent*-**1b**, do not exhibit antiprion activity at the tested concentration in cell-free PMSA systems and do not interfere with the propagation mechanisms of either bank vole- or mouse-derived recombinant prion strains. In contrast to the yeast data,^3^ no antiprion activity was observed under the mammalian in vitro conditions tested in this study. While these findings do not contradict the reported activity in yeast prions, the biological differences between the yeast and mammalian experimental systems should be considered when comparing these results. Nevertheless, the present data do not support the suggested broader interference with protein misfolding processes under the tested cell-free PMSA assays.

In addition to the mammalian antiprion assays, we studied the effects of all previously synthesized compounds on gamma-aminobutyric acid (GABA)-induced chloride currents (I_GABA_) at the predominantly expressed GABA_A_ receptor subtype composed of α1, β2, and γ2S subunits (Figure 6).

**Figure 6:**
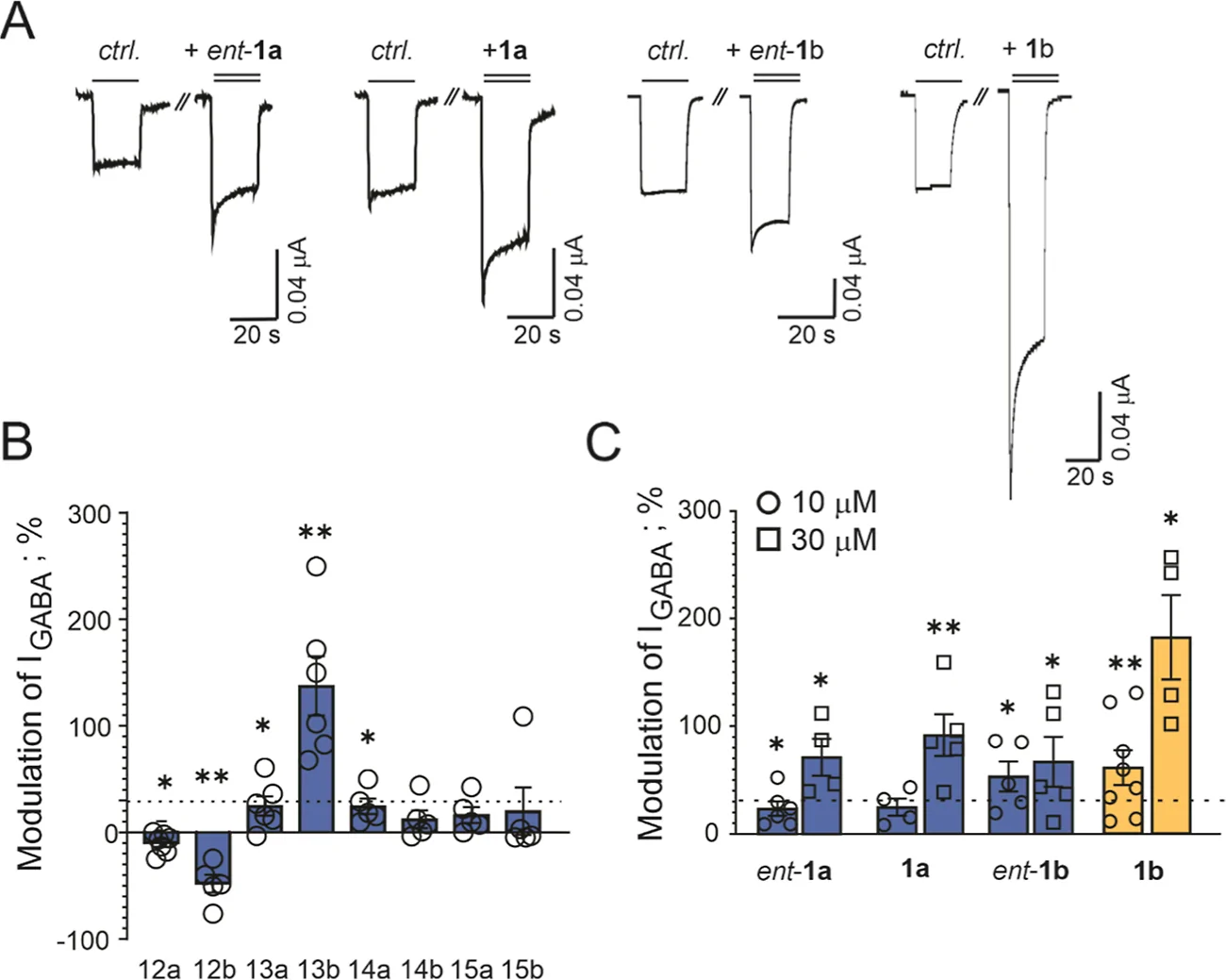
Effects of procerones and their synthetic precursors on GABA-induced chloride currents (I_GABA_) at α1β2γ2S GABA_A_ receptors. (A) Representative current traces showing I_GABA_ control currents (ctrl.; I_GABA_ in the absence of compound) and currents recorded in the presence of the indicated compounds (30 μm). (B) Modulation of I_GABA_ by the precursors (10 μm). (C) Modulation of I_GABA_ by procerones A (**1a**) and B (**1b**) and their corresponding enantiomers (*ent*-**1a** and *ent*-**1b**) at 10 μm (circles) and 30 μm (squares). Bars represent mean ± SEM, and individual experiments are shown as symbols (*n* = 4–5). The dashed line indicates the predefined threshold of 30% modulation above biological variability. Statistical significance was assessed using a one-sample *t*-test against the null hypothesis of no modulation (0%). * indicates *P* < 0.05, ** indicates *P* < 0.01.

Initial screening experiments were performed at 10 μm using a GABA concentration corresponding to approximately 10% of the maximal receptor response (EC_10_), enabling the detection of both positive and negative allosteric modulation. As shown in Figure 6B, several synthetic intermediates significantly altered I_GABA_ at 10 μm. Among these, epoxide **13b** emerged as the most efficacious positive modulator, enhancing I_GABA_ by 137.2 ± 27.7% (*t* = 4.953, *df* = 5, *P* = 0.0043). In contrast, chalcone **12b** significantly reduced I_GABA_ by 48.0 ± 8.4% (*t* = 5.705, *df* = 4, *P* = 0.0047), demonstrating that relatively small structural modifications within this scaffold can influence both the magnitude and direction of receptor modulation. The remaining intermediates produced either weak or no biologically relevant effects under the tested conditions. Given the activity observed during the initial screening, procerones A (**1a**) and B (**1b**) and their corresponding enantiomers (*ent*-**1a** and *ent*-**1b**) were evaluated in greater detail (Figure 6C). At 10 μm, only procerone B (**1b**; 61.0 ± 16.1%, *t* = 3.777, *df* = 7, *P* = 0.0069) and *ent*-**1b** (53.4 ± 13.9%, *t* = 3.842, *df* = 4, *P* = 0.0184) potentiated I_GABA_ above the predefined threshold for biological activity. In contrast, *ent*-**1a** produced only a modest, albeit significant, enhancement of I_GABA_ that was close to the predefined threshold for biological activity, whereas procerone A (**1a**) did not significantly modulate I_GABA_ at this concentration. Although epoxide **13b** displayed the highest efficacy in the initial screening, subsequent experiments focused on procerones A (**1a**) and B (**1b**) and their enantiomers (*ent*-**1a** and *ent*-**1b**), as these compounds represent the final natural products and were therefore the primary focus of the present study.

However, when tested at 30 μm, all four procerones significantly enhanced I_GABA_. Procerone A (**1a**) increased I_GABA_ by 91.7 ± 19.5% (*t* = 4.713, *df* = 4, *P* = 0.0092, *n* = 5), while its enantiomer *ent* **-1a** enhanced I_GABA_ by 71.5 ± 17.2% (*t* = 4.165, *df* = 3, *P* = 0.0252, *n* = 4). The strongest effect was observed for procerone B (**1b**), which enhanced I_GABA_ by 182.0 ± 39.2% (*t* = 4.638, *df* = 3, *P* = 0.0189, *n* = 4), whereas *ent*-**1b** increased I_GABA_ by 67.0 ± 23.2% (*t* = 2.885, *df* = 4, *P* = 0.0448, *n* = 5).

Taken together, these findings demonstrate that all four procerones positively modulate GABA_A_ receptor function. While procerone B (**1b**) displayed the highest efficacy, both enantiomers (**1b** and *ent*-**1b**) retained significant activity, indicating that stereochemistry is not the primary determinant of receptor modulation. The pronounced activity of intermediate **13b** and the concentration-dependent potentiation observed for the final natural products justify further investigation of this brominated marine natural product scaffold. Interestingly, intermediate **13b** displayed greater efficacy than the final natural products. Although the structural basis for this observation remains unclear and is beyond the scope of the present study, it suggests that this intermediate may provide a useful starting point for future medicinal chemistry optimization.

Positive modulation of α1β2γ2S receptors is typically associated with enhanced inhibitory neurotransmission^37^ and is characteristic of clinically used sedative, anxiolytic, and anticonvulsant agents acting at the GABAergic system such as benzodiazepines.^38^ Although the detailed mechanism of action and subtype selectivity remain to be determined, the present findings suggest that the fully brominated procerone scaffold may serve as a starting point for the development of novel GABA_A_ receptor modulators, particularly in light of the lack of antiprion activity observed in mammalian systems.

Finally, the synthesized compounds were also evaluated for their cytotoxic effects on HepG2 cells (a human hepatocellular cancer cell line) using a WST-1 assay (Supporting Information, Table S13 and Figure S47). Several compounds displayed moderate cytotoxicity with CC_50_ values ranging from 22.3 μm to >100 μm, indicating cellular uptake of these compounds in HepG2 cells. These observed cytotoxicities contribute to the overall biological profile of this compound class.

## Conclusion

For the first time, marine natural products procerones A (**1a**) and B (**1b**) were synthesized over three steps in 22% and 10% overall yield, respectively, together with their previously unreported enantiomers *ent*-**1a** and *ent*-**1b**. The core structure was efficiently obtained via Aldol condensation of commercially available acetophenone and benzaldehyde derivatives **10** and **11a**–**b**, respectively. Davis oxidation was employed to introduce the stereogenic center at a late stage and enabled the total synthesis without the use of heavy metals. Furthermore, the obtained SXRD structure of procerone A (**1a**) confirmed the successful synthesis of the reported natural product class, and the previously assigned stereochemistry was verified by optical rotation and ECD analysis. All synthesized compounds were biologically tested, and none of the four obtained enantiopure compounds **1a**, *ent*-**1a**, **1b**, and *ent*-**1b** were active against the tested mammalian prions. However, the previously unknown epoxide **13b** showed pronounced potentiation of GABA_A_ receptor function, together with procerone B (**1b**) and *ent* **-1b**, which also significantly enhanced I_GABA_. These findings suggest that the fully brominated scaffold may serve as an interesting starting point for the development of novel GABA_A_ receptor modulators. Future studies addressing subtype selectivity, mechanism of action, and structure–activity relationships will be required to further evaluate the pharmacological potential of this scaffold.

## Supplementary Material



## Notes

Synthetic procedures with complementing analytical data, screening results, purity analysis, SXRD details, additional ECD spectra with corresponding computational details, and biological testing procedures are provided in the Supporting Information. The authors have cited additional references within the Supporting Information.^39–71^ (PDF)

## Abbreviations

CCDC: Cambridge crystallographic data center
DFT: density functional theory
DMSO: dimethyl sulfoxide
ECD: electronic circular dichroism
*ee*: enantiomeric excess
*e.r.*: enantiomeric ratio
GABA: gamma-aminobutyric acid
HPLC: high-performance liquid chromatography
NMR: nuclear magnetic resonance
PMSA: protein misfolding shaking amplification
PrP: prion protein
PrP^res^: protease-resistant prion protein
SXRD: single-crystal X-ray diffraction
TFA: trifluoroacetic acid
