# Supplementary material for: Concise Total Synthesis and Pharmacological Evaluation of Procerones A and B
Source: ACS Pharmacol Transl Sci. 2026 Aug 21;9(9):2525–34. doi: 10.1021/acsptsci.6c00196 (PMC13632894; doi:10.1021/acsptsci.6c00196)
Supplement: Supplementary file 1 [file pt6c00196_si_001.pdf]

Supporting Information

Concise Total Synthesis and Pharmacological Evaluation of Procerones A and B

Jasmin Janneschütz,[a, b] Pia B. Gartlgruber,[a] Phila Weissenstein,[a] Christine Aczel,[a] Vinzenz M. Herkner,[a] Cornelia Shtepani,[a] Celina Haller,[a] Yuki Bacowsky,[a] Fatima Aslam,[a] Laureta Gashi,[a, b] Hasier Eraña,[c, d, e] Juan C. Fuentes-Monteverde,[g] Sarah Abel,[h] Stefan Simić,[a, b] Judith Wackerlig-Damle,[a] Arnold Grünweller,[h] Christian Griesinger,[g] Joaquín Castilla,[c, e, f] Sophia Khom,[a] and Nina Schützenmeister*[a, h]

[a] Department of Pharmaceutical Sciences, University of Vienna, Josef-Holaubek-Platz 2, 1090 Vienna, Austria

[b] Vienna Doctoral School of Pharmaceutical, Nutritional and Sport Sciences, University of Vienna, Josef-Holaubek-Platz 2, 1090 Vienna, Austria

[c] Center for Cooperative Research in Biosciences (CIC BioGUNE), Basque Research and Technology Alliance (BRTA), Bizkaia Technology Park, 48160 Derio, Spain

[d] ATLAS Molecular Pharma S. L., Bizkaia Technology Park, 48160 Derio, Spain

[e] Centro de Investigación Biomédica en Red de Enfermedades Infecciosas (CIBERINFEC), Carlos III National Health Institute, C/ Monforte de Lemos 3–5, 28029 Madrid, Spain

[f] IKERBASQUE, Basque Foundation for Science, Prion Research Lab, Plaza Euskadi 5, 48009 Bilbao, Spain

[g] Department of NMR-Based Structural Biology, Max Planck Institute for Multidisciplinary Sciences, Am Fassberg 11, 37077 Göttingen, Germany

[h] Institute of Pharmaceutical Chemistry, Philipps-Universität Marburg, Marbacher Weg 6–10, 35032 Marburg, Germany

*Corresponding author: nina.schuetzenmeister@uni-marburg.de

Contents

1 General information **S-3**

2 Synthetic procedures **S-4**

2.1 Aldol condensation S-4
2.2 Epoxidation S-6
2.3 Hydrogenation S-8
2.4 Davis oxidation S-12

3 NMR spectra **S-16**

4 Screening/Optimization **S-28**

4.1 Aldol condensation S-28
4.2 Epoxide ring-opening S-30

5 HPLC **S-31**

5.1 Procerone A **1a**/*ent*-**1a** S-31
5.2 Procerone B **1b**/*ent*-**1b** S-34

6 Purity analysis **S-37**

7 Single crystal X-ray diffraction **S-43**

7.1 Procerone A **1a** S-43

8 Electronic circular dichroism **S-45**

8.1 Experimental S-45
8.2 Calculated S-50

9 Biological evaluation **S-55**

9.1 Anti-prion testing S-55
9.2 $GABA_A$ testing S-56
9.3 Antiproliferation testing S-57

10 References **S-59**

1 General information

All reactions requiring anhydrous conditions were performed under an argon atmosphere in oven-dried glassware, applying *Schlenk* technique. Reactions carried out at 0 °C were performed in an ice bath. Reactions carried out at sub-zero temperatures employed cooling bath mixtures: −78 °C (dry ice/acetone), −60 °C (dry ice/diisopropylether), −40 °C (dry ice/acetonitrile), −20 °C (ice/NaCl 3:1). All commercially purchased chemicals were used without further purification unless otherwise noted. THF was dried by storing it over 4 Å molecular sieves under an argon atmosphere. Infrared spectra were obtained from neat solids or liquids on a Bruker Alpha with a Platinum-ATR unit. Ultraviolet spectra were acquired using a GeneQuant™ 1300 UV/Vis spectrometer by Healthcare Life Sciences, equipped with a quartz cuvette. Measurements were performed between 200–600 nm. Samples were dissolved in methanol or acetonitrile (c = 0.1 mg/mL, or further diluted until the maximum absorbance was ≤ 1). Melting points were determined in open capillary tubes using a Cole-Parmer® Stuart™ SMP11 melting point apparatus and are uncorrected. Optical rotations were measured using an Anton Paar MCP 100 polarimeter. Concentrations (c) of specific rotations are given in g/100 mL. High-resolution mass spectra (HRMS) were measured with a Bruker maXis HD ESI-QTOF or with a Bruker timsTOF flex ESI/MALDI-Qq-TOF with dual-TIMS analyzer. NMR spectra were recorded on a Bruker AVANCE III ASCEND 400 MHz spectrometer with BBFO-PLUS probe or on a Bruker AVANCE III HD Ultrashield 500 MHz spectrometer with TCI H/F-C-N Prodigy Cryoprobe head at 298 K. As internal standard, the residual signals of deuterated DMSO 2.50/39.52 ppm, deuterated chloroform 7.26/77.16 ppm and deuterated methanol 3.31/49.00 ppm (δ ^{1}H/^{13}C ppm) were used.[1] All relevant signals are listed as follows: chemical shift, multiplicity (s = singlet, d = doublet, dd = doublet of doublets, t = triplet, tt = triplet of triplets, m = multiplet), coupling constant(s) and number of protons/carbons. NMR tubes were used from deutero (Boroeco-5-7). Amber glass NMR tubes (5 mm, deutero) were used for light-sensitive samples. Reactions were monitored by thin-layer chromatography (TLC) on silica-coated aluminum plates (Macherey-Nagel, DC Kieselgel Alugram® Xtra SIL G/UV254, layer thickness 0.2 mm). Visualization was performed by Camag UV lamp (λ = 254 nm, 366 nm). Flash column chromatography was performed using silica 60 (particle size 0.040–0.063 nm, 230–400 mesh, Macherey-Nagel) at room temperature using an MPLC-system (Biotage® Selekt).

2 Synthetic procedures

2.1 Aldol condensation

2.1.1 *(E)*-1-(3',5'-dibromo-4'-hydroxyphenyl)-3-(3''-bromo-4''-methoxyphenyl)-prop-2-en-1-one 12a

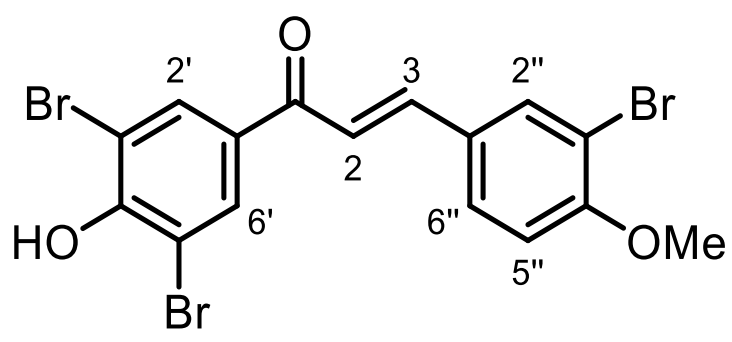

12a

To a solution of 3,5-dibromo-4-hydroxyacetophenone (500 mg, 1.70 mmol, 1.00 equiv.) and 3-bromo-4-methoxybenzaldehyde (366 mg, 1.70 mmol, 1.00 equiv.) in MeOH (3.00 mL) was added lithium hydroxide monohydrate (286 mg, 6.80 mmol, 4.00 equiv.). The reaction mixture was stirred at 55 °C for 3 h under microwave irradiation. Afterwards, the reaction mixture was diluted with EtOAc (20 mL) and was washed with 1 M aqueous HCl (2 × 15 mL) and water (15 mL). The aqueous phase was extracted with EtOAc (3 × 20 mL) and the combined organic phases were washed with brine (30 mL) and the solvent was removed in vacuum. The obtained mixture was recrystallized from acetonitrile to yield chalcone derivative **12a** (694 mg, 1.41 mmol, 83%) as pale-yellow solid.[a]

TLC: R_f = 0.24 (PE/EtOAc 2:1).
Mp.: 222 °C.
IR (ATR): $\tilde{\nu}$ (cm^{-1}) = 3165, 3004, 2924, 2838, 1585, 1535, 1333, 1294, 1262, 1195, 1135, 1047, 1012, 804, 732.
UV (MeCN): λ_{max} (nm) = 207, 339.
^{1}H-NMR (400 MHz, DMSO-d_6): δ (ppm) = 3.92 (s, 3 H, OCH_3), 7.18 (d, *J* = 8.7 Hz, 1 H, 5''-H), 7.68 (d, *J* = 15.4 Hz, 1 H, 2-H), 7.87–7.93 (m, 2 H, 3-H, 6''-H), 8.32 (d, *J* = 2.1 Hz, 1 H, 2''-H), 8.37 (s, 2 H, 2'-H, 6'-H), 10.96 (s, 1 H, OH).
^{13}C-NMR (126 MHz, DMSO-d_6): *δ* (ppm) = 56.5 (OCH_3), 111.4 (C-3''), 111.8 (2 C, C-3', C-5'), 112.7 (C-5''), 120.0 (C-2), 128.8 (C-1''), 131.2 (C-6''), 131.5 (C-1'), 132.7 (C-2''), 132.9 (2 C, C-2', C-6'), 142.9 (C-3), 155.0 (C-4'), 157.2 (C-4''), 185.1 (C-1).
HRMS (ESI^-) = *m/z* calcd: 486.8186 $C_{16}H_{10}Br_3O_3^-$; found: 486.8193.
$C_{16}H_{11}Br_3O_3$ (490.97 g/mol).

[a] Note: chalcone derivative **12a** is light-sensitive; the synthetic procedure was performed under the exclusion of light.

2.1.2 *(E)*-1-(3',5'-dibromo-4'-hydroxyphenyl)-3-(3",5"-dibromo-4'-methoxyphenyl)-prop-2-en-1-one 12b

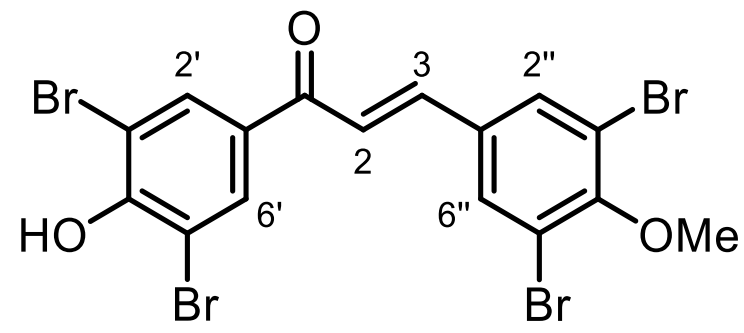

12b

To a solution of 3,5-dibromo-4-hydroxyacetophenone (750 mg, 2.55 mmol, 1.00 equiv.) and 3,5-dibromo-4-methoxybenzaldehyde (750 mg, 2.55 mmol, 1.00 equiv.) in MeOH (3.20 mL) was added lithium hydroxide monohydrate (428 mg, 10.2 mmol, 4.00 equiv.). The reaction mixture was stirred at 55 °C for 3 h under microwave irradiation. Afterwards, the reaction mixture was diluted with EtOAc (30 mL) and washed with 1 M aqueous HCl (2 × 20 mL) and water (20 mL). The aqueous phase was extracted with EtOAc (3 × 30 mL), and the combined organic phases were washed with brine (50 mL). The solvent was removed in vacuum, and the obtained mixture was recrystallized from acetonitrile to yield chalcone derivative **12b** (834 mg, 1.46 mmol, 57%) as a pale-yellow solid.[b]

TLC: R_f = 0.36 (PE/EtOAc 3:2).
Mp.: 231 °C.
IR (ATR): $\tilde{\nu}$ (cm^{-1}) = 3168, 1652, 1588, 1534, 1472, 1398, 1316, 1265, 1200, 1154, 979, 836, 736, 580, 515.
UV (MeCN): λ_{max} (nm) = 209, 316.
^{1}H-NMR (500 MHz, DMSO-d_6): δ (ppm) = 3.85 (s, 3 H, OCH_3), 7.66 (d, J = 15.5 Hz, 1 H, 3-H), 8.02 (d, J = 15.5 Hz, 1 H, 2-H), 8.30 (s, 2 H, 2"-H, 6"-H), 8.38 (s, 2 H, 2'-H, 6'-H), 11.02 (s, 1 H, OH).
^{13}C-NMR (126 MHz, DMSO-d_6): δ (ppm) = 60.6 (OCH_3), 111.8 (2 C, C-3', C-5'), 118.0 (2 C, C-3'', C-5''), 123.0 (C-2), 131.2 (C-1'), 133.0 (2 C, C-2'', C-6''), 133.1 (2 C, C-2', C-6'), 134.1 (C-1''), 140.9 (C-3), 154.7 (C-4''), 155.3 (C-4'), 185.1 (C-1).
HRMS (ESI^-) = m/z calcd: 564.7291 $C_{16}H_9Br_4O_3^-$; found: 564.7293.
$C_{16}H_{10}Br_4O_3$ (569.87 g/mol).

[b] Note: chalcone derivative **12b** is light-sensitive; the synthetic procedure was performed under the exclusion of light.

2.2 Epoxidation

2.2.1 1-(3‘,5‘-Dibromo-4‘-hydroxyphenyl)-3-(3“-bromo-4“-methoxyphenyl)-prop-2-epoxy-1-one 13a

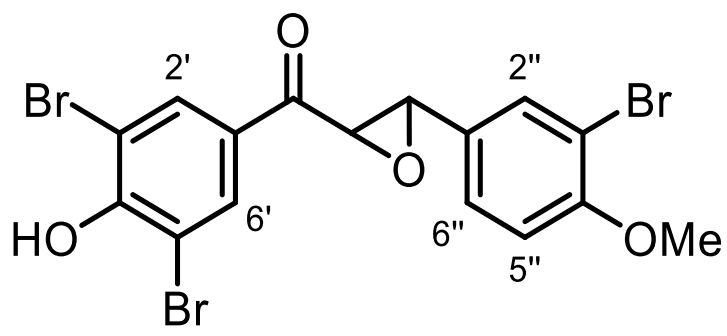

13a

To a solution of chalcone derivative **12a** (50.0 mg, 102 µmol, 1.00 equiv.) in MeOH (3.00 mL) was added an aqueous NaOH-solution (20%, 67.0 µL, 81.5 mg, 407 µmol, 4.00 equiv.). The reaction mixture was cooled to 0 °C and subsequently a H_2O_2-solution (30%, 62.0 µL, 69.3 mg, 611 µmol, 6.00 equiv.) was added dropwise. Afterwards, the reaction mixture was stirred at 0 °C for 4.5 h, allowing it to slowly warm to room temperature. The reaction mixture was diluted with EtOAc (10 mL) and washed with water (10 mL) and brine (10 mL). The aqueous phase was extracted with EtOAc (3 × 10 mL). The combined organic phases were washed with brine (20 mL), dried over MgSO4, filtered and the solvent was removed in vacuum. The obtained mixture was purified by reversed phase column chromatography (RP-C_{18}, 19:1 → 1:1 H_2O/MeCN) to yield chalcone epoxide **13a** (41.9 mg, 82.6 µmol, 81%) as yellow solid.[c]

TLC: R_f = 0.23 (PE/EtOAc 2:3).
Mp.: 202 °C.
IR (ATR): $\tilde{\nu}$ (cm^{-1}) = 3236, 2838, 2114, 1638, 1560, 1485, 1417, 1354, 1229, 1049, 1014, 905, 807, 669, 590.
UV (MeOH): λ_{max} (nm) = 207, 351.
^{1}H-NMR (400 MHz, DMSO-d_6): δ (ppm) = 3.86 (s, 3°H, OCH_3), 3.95 (d, J = 2.0 Hz, 1 H, 3-H), 4.57 (d, J = 2.0 Hz, 1 H, 2-H), 7.12 (d, J = 8.5 Hz, 1 H, 5''-H), 7.43 (dd, J = 8.5 Hz, 2.1 Hz, 1 H, 6''-H), 7.63 (d, J = 2.1 Hz, 1 H, 2''-H), 7.96 (s, 2 H, 2'-H, 6'-H).
^{13}C-NMR (101 MHz, DMSO-d_6): δ (ppm) = 56.3 (OCH_3), 56.8 (C-3), 58.4 (C-2), 110.7 (C-3''), 112.5 (C-5''), 115.1 (C-1'), 116.9 (2 C, C-3', C-5'), 127.5 (C-2'') 130.0 (C-1''), 130.7 (C-2''), 132.4 (2 C, C-2', C-6'), 155.6 (C-4''), 166.6 (C-4'), 185.4 (C-1).
HRMS (ESI^-) = m/z calcd: 502.8135 $C_{16}H_{10}Br_3O_4^-$; found: 502.8138.
$C_{16}H_{11}Br_3O_4$ (506.97 g/mol).

[c] Note: chalcone epoxide **13a** is light-sensitive; the synthetic procedure was performed under the exclusion of light.

2.2.2 1-(3‘,5‘-Dibromo-4‘-hydroxyphenyl)-3-(3“,5”-dibromo-4“-methoxyphenyl)-prop-2-epoxy-1-one 13b

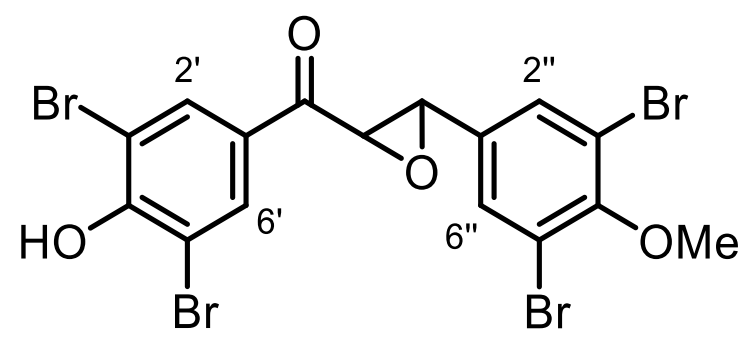

13b

To a solution of chalcone derivative **12b** (410 mg, 719 µmol, 1.00 equiv.) in MeOH (24.6 mL) was added an aqueous NaOH-solution (20%, 472 µL, 576 mg, 2.88 mmol, 4.00 equiv.). The reaction mixture was cooled to 0 °C and subsequently a H_2O_2-solution (30%, 441 µL, 489 mg, 4.32 mmol, 6.00 equiv.) was added dropwise. Afterwards, the reaction mixture was stirred at 0 °C for 4.5 h, allowing it to slowly warm to room temperature. The reaction mixture was diluted with EtOAc (25 mL) and washed with water (30 mL) and brine (30 mL). The aqueous phase was extracted with EtOAc (3 × 30 mL). The combined organic phases were washed with brine (60 mL), dried over MgSO4, filtered and the solvent was removed in vacuum. The obtained mixture was purified by reversed phase column chromatography (RP-C_{18} 19:1 → 2:1 H_2O/MeCN) to yield chalcone epoxide **13b** (216 mg, 369 µmol, 51%) as yellow solid.[d]

TLC: R_f = 0.23 (PE/EtOAc 1:2).
Mp.: 210 °C.
IR (ATR): $\tilde{\nu}$ (cm^{-1}) = 3752, 3235, 1636, 1559, 1478, 1413, 1379, 1354, 1227, 1066, 987, 861, 727, 670, 592.
UV (MeOH): λ_{max} (nm) = 208, 354.
^{1}H-NMR (500 MHz, DMSO-d_6): δ (ppm) = 3.81 (s, 3 H, OCH_3), 4.02 (d, J = 1.8 Hz, 1 H, 3-H), 4.65 (d, J = 1.8 Hz, 1 H, 2-H), 7.72 (s, 2 H, 2’’-H, 6’’-H), 7.99 (s, 2 H, 2’-H, 6’-H).
^{13}C-NMR (126 MHz, DMSO-d_6): δ (ppm) = 55.9 (C-3), 58.0 (C-2), 60.4 (OCH_3), 115.2 (C-1’), 117.0 (2 C, C-3’, C-5’), 117.6 (2 C, C-3’’, C-5’’), 130.8 (2 C, C-2’’, C-6’’), 132.5 (2 C, C-2’, C-6’), 136.0 (C-1’’), 153.4 (C-4’’), 166.7 (C-4’), 184.9 (C-1).
HRMS (ESI^-) = m/z calcd: 580.7240 $C_{16}H_9Br_4O_4^-$; found: 580.7247.
$C_{16}H_{10}Br_4O_4$ (585.87 g/mol).

[d] Note: chalcone epoxide **13b** is light-sensitive; the synthetic procedure was performed under the exclusion of light.

2.3 Hydrogenation

2.3.1 1-(3',5'-Dibromo-4'-hydroxyphenyl)-3-(3''-bromo-4''-methoxyphenyl)-propan-1-one 14a

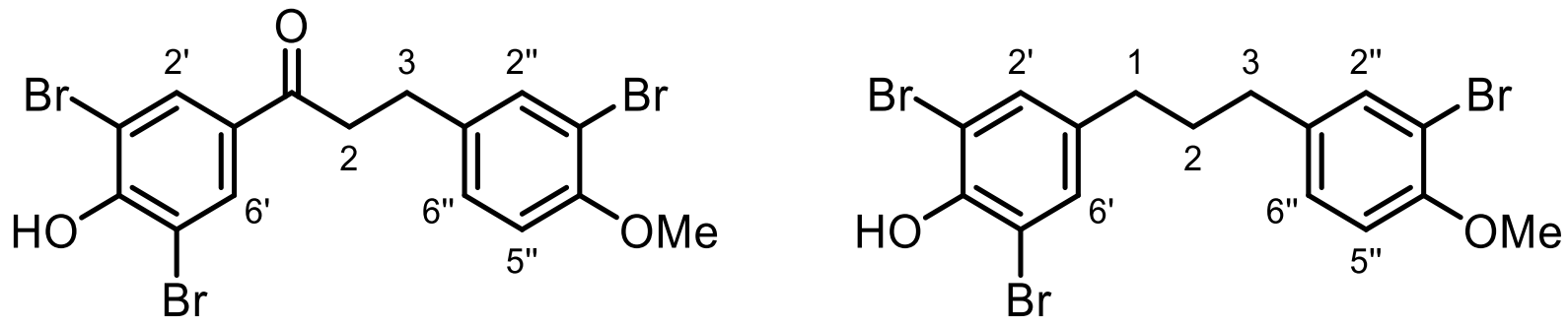

14a **15a**

To a solution of chalcone derivative **12a** (403 mg, 821 μmol, 1.00 equiv.) in anhydrous CH_2Cl_2 (8.00 mL) were added trifluoroacetic acid (632 μL, 936 mg, 8.21 mmol, 10.0 equiv.) and triethylsilane (524 μL, 382 mg, 3.28 mmol, 4.00 equiv.) and stirred at room temperature for 18 h. Afterwards, the reaction mixture was diluted with CH_2Cl_2 (15 mL) and washed with water (2 × 20 mL). The aqueous phase was extracted with CH_2Cl_2 (3 × 15 mL) and the combined organic phases were washed with brine (30 mL). dried over $MgSO_4$ and filtered. Solid SiO_2 (2.50 g) was added to the organic phase and the solvent was carefully removed in vacuum. The obtained mixture was purified by column chromatography (SiO_2, PE/EtOAc 19:1 → 3:1) to yield the hydrogenated by-product **15a** (70.0 mg, 146 μmol, 18%) as colorless oil, which slowly crystallized, followed by the hydrogenated derivative **14a** (328 mg, 664 μmol, 81%) as beige solid.

Analytical data for **14a**:

TLC: R_f = 0.45 (PE/EtOAc 2:1).
Mp.: 117 °C.
IR (ATR): $\tilde{\nu}$ (cm^{-1}) = 3277, 3002, 2919, 1735, 1668, 1543, 1499, 1310, 1280, 1257, 1190, 1050, 803, 731, 574.
UV (MeCN): λ_{max} (nm) = 205, 220, 270.
^{1}H-NMR (500 MHz, DMSO-d_6): δ (ppm) = 2.83 (t, J = 7.4 Hz, 2 H, 3-H_2), 3.32 (t, J = 7.4 Hz, 2 H, 2-H_2), 3.80 (s, 3 H, OCH_3), 7.00 (d, J = 8.4 Hz, 1 H, 5''-H), 7.24 (dd, J = 8.4, 2.2 Hz, 1 H, 6''-H), 7.50 (d, J = 2.2 Hz, 1 H, 2''-H), 8.11 (s, 2 H, 2'-H, 6'-H), 10.90 (s, 1 H, OH).
^{13}C-NMR (126 MHz, DMSO-d_6): δ (ppm) = 28.1 (C-3), 38.9 (C-2), 56.1 (OCH_3), 110.3 (C-3''), 111.6 (2 C, C-3', C-5'), 112.5 (C-5''), 128.9 (C-6''), 130.7 (C-1''), 132.3 (2 C, C-2', C-6'), 132.7 (C-2''), 134.9 (C-1'), 153.6 (C-4''), 154.9 (C-4'), 195.9 (C-1).
HRMS (ESI^-) = m/z calcd: 488.8342 $C_{16}H_{12}Br_3O_3^-$; found: 488.8335.
$C_{16}H_{13}Br_3O_3$ (492.99 g/mol).

<u>Analytical data for **15a**:</u>

TLC: R_f = 0.31 (PE/EtOAc 9:1).

Mp.: 42 °C.

IR (ATR): $\tilde{\nu}$ (cm^{-1}) = 3476, 2936, 2854, 1603, 1494, 1467, 1403, 1251, 1113, 1018, 863, 803, 739, 706, 554.

UV (MeCN): λ_{max} (nm) = 204.

^{1}H-NMR (500 MHz, methanol-d_4): δ (ppm) = = 1.84 (tt, *J* = 7.7, 7.4 Hz, 2 H, 2-H_2), 2.49–2.56 (m, 4 H, 1-H_2, 3-H_2), 3.84 (s, 3 H, OCH_3), 6.93 (d, *J* = 8.4 Hz, 1 H, 5''-H), 7.11 (dd, *J* = 8.4, 2.2 Hz, 1 H, 6''-H), 7.28 (s, 2 H, 2'-H, 6'-H), 7.34 (d, *J* = 2.2 Hz, 1 H, 2''-H).

^{13}C-NMR (126 MHz, methanol-d_4): *δ* (ppm) = 34.2 (C-2), 34.7 (C-1), 34.9 (C-3), 56.7 (OCH_3), 112.1 (2 C, C-3', C-5'), 112.3 (C-3''), 113.3 (C-5''), 129.6 (C-6''), 133.2 (2 C, C-2', C-6'), 134.1 (C-2''), 137.0 (C-1''), 137.8 (C-1'), 150.3 (C-4'), 155.6 (C-4'').

HRMS (ESI^-) = *m/z* calcd: 474.8549 $C_{16}H_{14}Br_3O_2^-$; found: 474.8529.

$C_{16}H_{15}Br_3O_2$ (479.01 g/mol).

2.3.2 1-(3',5'-Dibromo-4'-hydroxyphenyl)-3-(3",5"-dibromo-4"-methoxyphenyl)-propan-1-one 14b

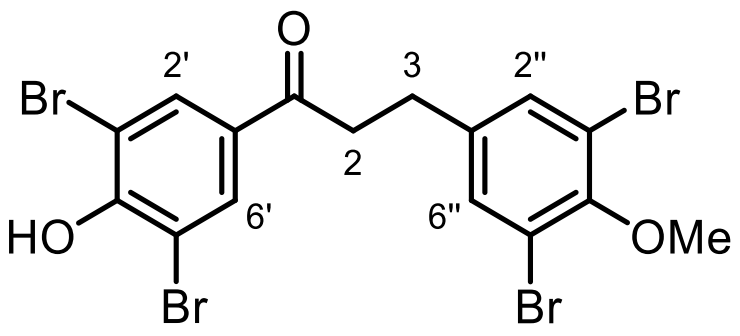

14b

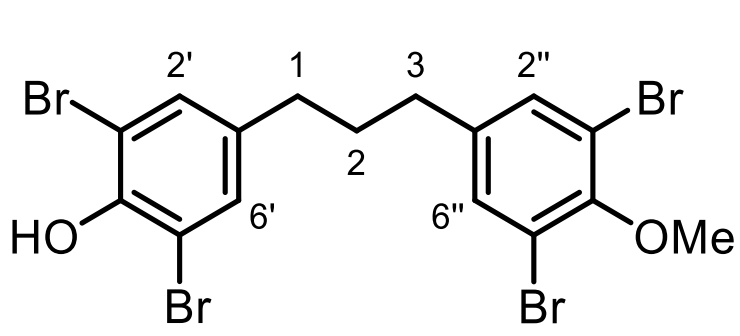

15b

To a solution of chalcone derivative **12b** (500 mg, 877 µmol, 1.00 equiv.) in anhydrous CH_2Cl_2 (8.00 mL) were added trifluoroacetic acid (676 µL, 1.00 g, 8.77 mmol, 10.0 equiv.) and triethylsilane (981 µL, 714 mg, 6.14 mmol, 7.00 equiv.) and the mixture was stirred at 75 °C for 10 h under microwave irradiation. Afterwards, the reaction mixture was diluted with EtOAc (10 mL) and washed with water (15 mL) and brine (15 mL). The aqueous phase was extracted with EtOAc (3 × 20 mL) and the combined organic phases were washed with brine (30 mL), dried over $MgSO_4$ and filtered. Solid SiO_2 (2.50 g) was added to the organic phase and the solvent was removed in vacuum. The obtained mixture was purified by column chromatography (SiO_2, PE/EtOAc 19:1 → 1:1) to yield the hydrogenated by-product **15b** (147 mg, 257 µmol, 29%) as pale-yellow solid, followed by the hydrogenated derivative **14b** (301 mg, 526 µmol, 60%) as pale-yellow solid.

Analytical data for **14b**:

TLC: R_f = 0.49 (PE/EtOAc 1:1).
Mp.: 177 °C.
IR (ATR): $\tilde{\nu}$ (cm^{-1}) = 3169, 2921, 1671, 1539, 1470, 1397, 1301, 1252, 1106, 1150, 990, 732, 578, 552, 520.
UV (MeOH): λ_{max} (nm) = 207, 275, 334.
^{1}H-NMR (500 MHz, DMSO-d_6): δ (ppm) = 2.86 (t, J = 7.4 Hz, 2 H, 3-H_2), 3.37 (t, J = 7.4 Hz, 2 H, 2-H_2), 3.76 (s, 3 H, OCH_3), 7.60 (s, 2 H, 2"-H, 6"-H), 8.12 (s, 2 H, 2'-H, 6'-H), 10.91 (s, 1 H, OH).
^{13}C-NMR (126 MHz, DMSO-d_6): δ (ppm) = 27.9 (C-3), 38.5 (C-2), 60.3 (OCH_3), 111.6 (2 C, C-3', C-5'), 117.0 (2 C, C-3'', C-5''), 130.6 (C-1'), 132.4 (2 C, C-2', C-6'), 132.8 (2 C, C-2'', C-6''), 140.8 (C-1''), 151.4 (C-4''), 154.9 (C-4'), 195.7 (C-1).
HRMS (ESI^-) = m/z calcd: 566.7447 $C_{16}H_{11}Br_4O_3^-$; found: 566.7440.
$C_{16}H_{12}Br_4O_3$ (571.88 g/mol).

Analytical data for **15b**:

TLC: R_f = 0.44 (PE/EtOAc 9:1).

Mp.: 93 °C.

IR (ATR): $\tilde{\nu}$ (cm^{-1}) = 3480, 2941, 2857, 2074, 1548, 1468, 1399, 1318, 1256, 1161, 990, 858, 736, 606, 553.

UV (MeCN): λ_{max} (nm) = 206.

^{1}H-NMR (500 MHz, methanol-d_4): δ (ppm) = 1.87 (tt, J = 7.8, 7.5 Hz, 2 H, 2-H_2), 2.52–2.59 (m, 4 H, 1-H_2, 3-H_2), 3.82 (s, 3 H, OCH_3), 7.29 (s, 2 H, 2'-H, 6'-H), 7.38 (s, 2 H, 2''-H, 6''-H).

^{13}C-NMR (126 MHz, methanol-d_4): δ (ppm) = 33.6 (C-2), 34.7 (C-1), 34.9 (C-3), 61.0 (OCH_3), 112.2 (2 C, C-3', C-5'), 118.7 (2 C, C-3'', C-5''), 133.3 (2 C, C-2', C-6'), 133.9 (2 C, C-2'', C-6''), 137.4 (C-1'), 142.6 (C-1''), 150.4 (C-4'), 153.4 (C-4'').

HRMS (ESI$^-$) = m/z calcd: 552.7655 $C_{16}H_{13}Br_4O_2^-$; found: 552.7634.

$C_{16}H_{14}Br_4O_2$ (557.90 g/mol).

2.4 Davis oxidation

2.4.1 Procerone A 1a

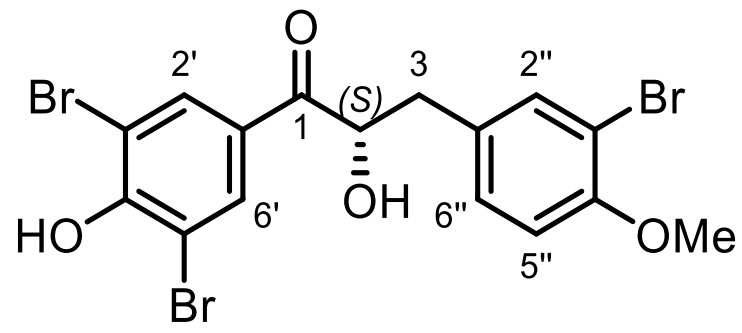

1a

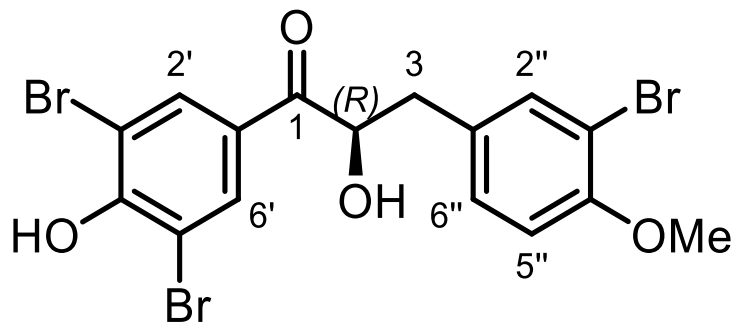

ent-**1a**

A solution of derivative **14a** (200 mg, 406 µmol, 1.00 equiv.) in anhydrous THF (12.0 mL) was cooled to −78 °C, a solution of NaHMDS (2 M in THF, 609 µL, 223 mg, 1.22 mmol, 3.00 equiv.) was added dropwise, and the mixture was stirred at −78 °C for 30 min. Afterwards, a separate solution of (1*S*)-(+)-(10-camphorsulfonyl)oxaziridine (140 mg, 609 µmol, 1.50 equiv.) in anhydrous THF (6.0 mL) was cooled to −78 °C, added dropwise to the reaction mixture and stirred at −78 °C for 30 min. Then, saturated aqueous NH_4Cl-solution (6.0 mL) and EtOAc (12 mL) were added at −78 °C and the mixture was warmed to room temperature. The phases were separated and the organic phase was washed with aqueous $Na_2S_2O_3$-solution (2 × 15 mL). The combined aqueous phases were extracted with EtOAc (3 × 20 mL) and the combined organic phases were washed with brine (30 mL), dried over $MgSO_4$, filtered and the solvent was removed in vacuum (*e.r.* 59:41). The obtained mixture was purified by column chromatography (SiO_2, dry load, PE/EtOAc 9:1 → 1:1) followed by recrystallization from MeOH to yield an enantiomeric mixture of procerone A **1a** and *ent*-**1a** (68.0 mg, 130 µmol, 33%, *e.r.* 54:46) as white solid. The enantiomers were separated by semi-preparative HPLC.

<u>Analytical data for procerone A **1a**:</u>

TLC: R_f = 0.18 (PE/EtOAc 1:2).
Mp.: 135 °C.
$[\boldsymbol{\alpha}]_{\mathbf{D}}^{\mathbf{25}}$: +13.0 (c = 0.10, MeOH).
IR (ATR): $\tilde{\nu}$ (cm^{-1}) = 3368, 2917, 2842, 2360, 2183, 2050, 1661, 1578, 1544, 1490, 1253, 1162, 1087, 800, 669.
UV (MeCN): λ_{max} (nm) = 220, 275, 358.
^{1}H-NMR (400 MHz, DMSO-d_6): δ (ppm) = 2.77 (dd, *J* = 14.0, 8.1 Hz, 1 H, 3-H_A), 2.93 (dd, *J* = 14.0, 4.9 Hz, 1 H, 3-H_B), 3.80 (s, 3 H, OCH_3), 4.98–5.01 (m, 1 H, 2-H), 5.65 (s, 1 H, 2-OH), 6.99 (d, *J* = 8.4 Hz, 1 H, 5''-H), 7.17 (dd, *J* = 8.4, 2.1 Hz, 1 H, 6''-H), 7.44 (d, *J* = 2.1 Hz, 1 H, 2''-H), 8.10 (s, 2 H, 2'-H, 6'-H), 10.97 (s, 1 H, 4'-OH).
^{13}C-NMR (101 MHz, DMSO-d_6): *δ* (ppm) = 38.2 (C-3), 56.1 (OCH_3), 73.1 (C-2), 110.1 (C-3''), 111.4 (2 C, C-3', C-5'), 112.3 (C-5''), 129.1 (C-1') 130.0 (C-6''), 131.7 (C-1''), 133.2 (2 C, C-2', C-6'), 133.7 (C-2''), 153.8 (C-4''), 155.0 (C-4'), 197.1 (C-1).
HRMS (ESI$^-$) = *m/z* calcd: 504.8291 $C_{16}H_{12}Br_3O_4^-$; found: 504.8300.
$C_{16}H_{13}Br_3O_4$ (508.99 g/mol).
Analytical data reported in the literature.[2]

Analytical data for *ent*-**1a**:

TLC: R_f = 0.18 (PE/EtOAc 1:2).

Mp.: 128 °C.

$[\alpha]_D^{25}$: −16.5 (c = 0.10, MeOH).

IR (ATR): $\tilde{\nu}$ (cm^{-1}) = 3378, 2922, 2844, 2361, 2181, 2083, 1660, 1578, 1543, 1489, 1253, 1160, 1087, 799, 667.

UV (MeCN): λ_{max} (nm) = 221, 275, 358.

^{1}H-NMR (400 MHz, DMSO-d_6): δ (ppm) = 2.77 (dd, *J* = 14.0, 8.1 Hz, 1 H, 3-H_A), 2.93 (dd, *J* = 14.0, 4.9 Hz, 1 H, 3-H_B), 3.80 (s, 3 H, OCH_3), 4.98–5.01 (m, 1 H, 2-H), 5.66 (s, 1 H, 2-OH), 6.99 (d, *J* = 8.4 Hz, 1 H, 5''-H), 7.17 (dd, *J* = 8.4, 2.1 Hz, 1 H, 6''-H), 7.44 (d, *J* = 2.1 Hz, 1 H, 2''-H), 8.10 (s, 2 H, 2'-H, 6'-H), 10.96 (s, 1 H, 4'-OH).

^{13}C-NMR (101 MHz, DMSO-d_6): *δ* (ppm) = 38.2 (C-3), 56.1 (OCH_3), 73.1 (C-2), 110.1 (C-3''), 111.4 (2 C, C-3', C-5'), 112.3 (C-5''), 129.1 (C-1') 130.0 (C-6''), 131.7 (C-1''), 133.2 (2 C, C-2', C-6'), 133.7 (C-2''), 153.9 (C-4''), 155.0 (C-4'), 197.1 (C-1).

HRMS (ESI^-) = *m/z* calcd: 504.8291 $C_{16}H_{12}Br_3O_4^-$ found: 504.8308.

$C_{16}H_{13}Br_3O_4$ (508.99 g/mol).

2.4.2 Procerone B 1b

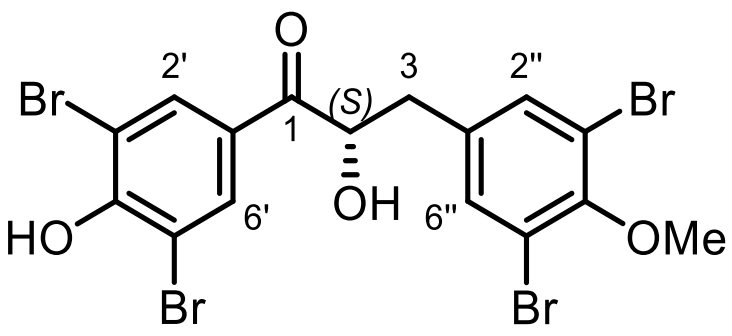

1b

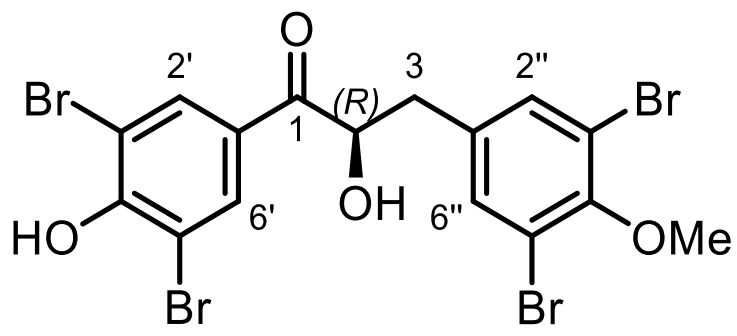

ent-**1b**

A solution of derivative **14b** (218 mg, 381 μmol, 1.00 equiv.) in anhydrous THF (13.6 mL) was cooled to −78 °C, a solution of NaHMDS (2 M in THF, 572 μL, 210 mg, 1.14 mmol, 3.00 equiv.) was added dropwise, and the mixture was stirred at −78 °C for 30 min. Afterwards, a separate solution of (1*S*)-(+)-(10-camphorsulfonyl)oxaziridine (131 mg, 572 μmol, 1.50 equiv.) in anhydrous THF (6.0 mL) was cooled to −78 °C, added dropwise to the reaction mixture, and stirred at −78 °C for 30 min. Then, saturated aqueous NH_4Cl-solution (6.0 mL) and EtOAc (12 mL) were added at −78 °C and the mixture was warmed to room temperature. The phases were separated and the organic phase was washed with $Na_2S_2O_3$-solution (2 × 15 mL). The combined aqueous phases were extracted with EtOAc (3 × 20 mL) and the combined organic phases were washed with brine (30 mL), dried over $MgSO_4$, filtered and the solvent was removed in vacuum (*e.r.* 64:36). The obtained mixture was purified by trituration with $CHCl_3$ followed by column chromatography (SiO_2, dry load, PE/EtOAc 9:1 → 1:1) to yield an enantiomeric mixture of procerone B **1b** and *ent*-**1b** (67.4 mg, 115 μmol, 30%, *e.r.* 62:38) as white solid. The enantiomers were separated by semi-preparative HPLC.

<u>Analytical data for procerone B **1b**:</u>

TLC: R_f = 0.22 (PE/EtOAc 1:2).
Mp.: 154 °C.
$[\alpha]_D^{25}$: +24.0 (c = 0.10, MeOH).
IR (ATR): $\tilde{\nu}$ (cm^{-1}) = 3401, 3197, 2923, 2858, 2128, 1667, 1542, 1472, 1334, 1252, 1208, 1163, 1093, 997, 736.
UV (MeCN): λ_{max} (nm) = 207, 274, 359.
^{1}H-NMR (400 MHz, DMSO-d_6): δ (ppm) = 2.77 (dd, *J* = 14.0, 8.7 Hz, 1 H, 3-H_A), 2.94 (dd, *J* = 14.0, 4.6 Hz, 1 H, 3-H_B), 3.76 (s, 3 H, OCH_3), 5.07 (dd, *J* = 8.7, 4.6 Hz, 1 H, 2-H), 5.69 (s, 1 H, 2-OH), 7.54 (s, 2 H, 2''-H, 6''-H), 8.11 (s, 2 H, 2'-H, 6'-H), 10.97 (s, 1 H, 4'-OH).
^{13}C-NMR (101 MHz, DMSO-d_6): *δ* (ppm) = 37.9 (C-3), 60.3 (OCH_3), 72.4 (C-2), 111.4 (2 C, C-3', C-5'), 116.8 (2 C, C-3'', C-5''), 129.1 (C-1'), 133.2 (2 C, C-2', C-6'), 133.8 (2 C, C-2'', C-6''), 137.8 (C-1''), 151.6 (C-4''), 155.0 (C-4'), 196.9 (C-1).
HRMS (ESI$^-$) = *m/z* calcd: 582.7396 $C_{16}H_{11}Br_4O_4^-$ found: 582.7417.
$C_{16}H_{12}Br_4O_4$ (587.88 g/mol).
Analytical data reported in the literature.[2]

Analytical data for *ent*-**1b**:

TLC: R_f = 0.22 (PE/EtOAc 1:2).

Mp.: 151 °C.

$[\alpha]_D^{25}$: −23.5 (c = 0.10, MeOH).

IR (ATR): $\tilde{\nu}$ (cm^{-1}) = 3999, 3204, 2923, 2857, 2117, 1668, 1543, 1469, 1336, 1252, 1209, 1164, 1093, 1005, 736.

UV (MeCN): λ_{max} (nm) = 207, 274, 359.

^{1}H-NMR (400 MHz, DMSO-d_6): δ (ppm) = 2.77 (dd, *J* = 8.7, 14.0 Hz, 1 H, 3-H_A), 2.94 (dd, *J* = 4.6, 14.0 Hz, 1 H, 3-H_B), 3.76 (s, 3 H, OCH_3), 5.07 (dd, *J* = 4.6, 8.7 Hz, 1 H, 2-H), 5.71 (s, 1 H, 2-OH), 7.54 (s, 2 H, 2''-H, 6''-H), 8.12 (s, 2 H, 2'-H, 6'-H), 10.96 (s, 1 H, 4'-OH).

^{13}C-NMR (101 MHz, DMSO-d_6): *δ* (ppm) = 37.9 (C-3), 60.3 (OCH_3), 72.4 (C-2), 111.4 (2 C, C-3', C-5'), 116.8 (2 C, C-3'', C-5''), 129.1 (C-1'), 133.2 (2 C, C-2', C-6'), 133.8 (2 C, C-2'', C-6''), 137.8 (C-1''), 151.6 (C-4''), 155.0 (C-4'), 196.9 (C-1).

HRMS (ESI^-) = *m/z* calcd: 582.7396 $C_{16}H_{11}Br_4O_4^-$ found: 582.7421.

$C_{16}H_{12}Br_4O_4$ (587.88 g/mol).

3 NMR spectra

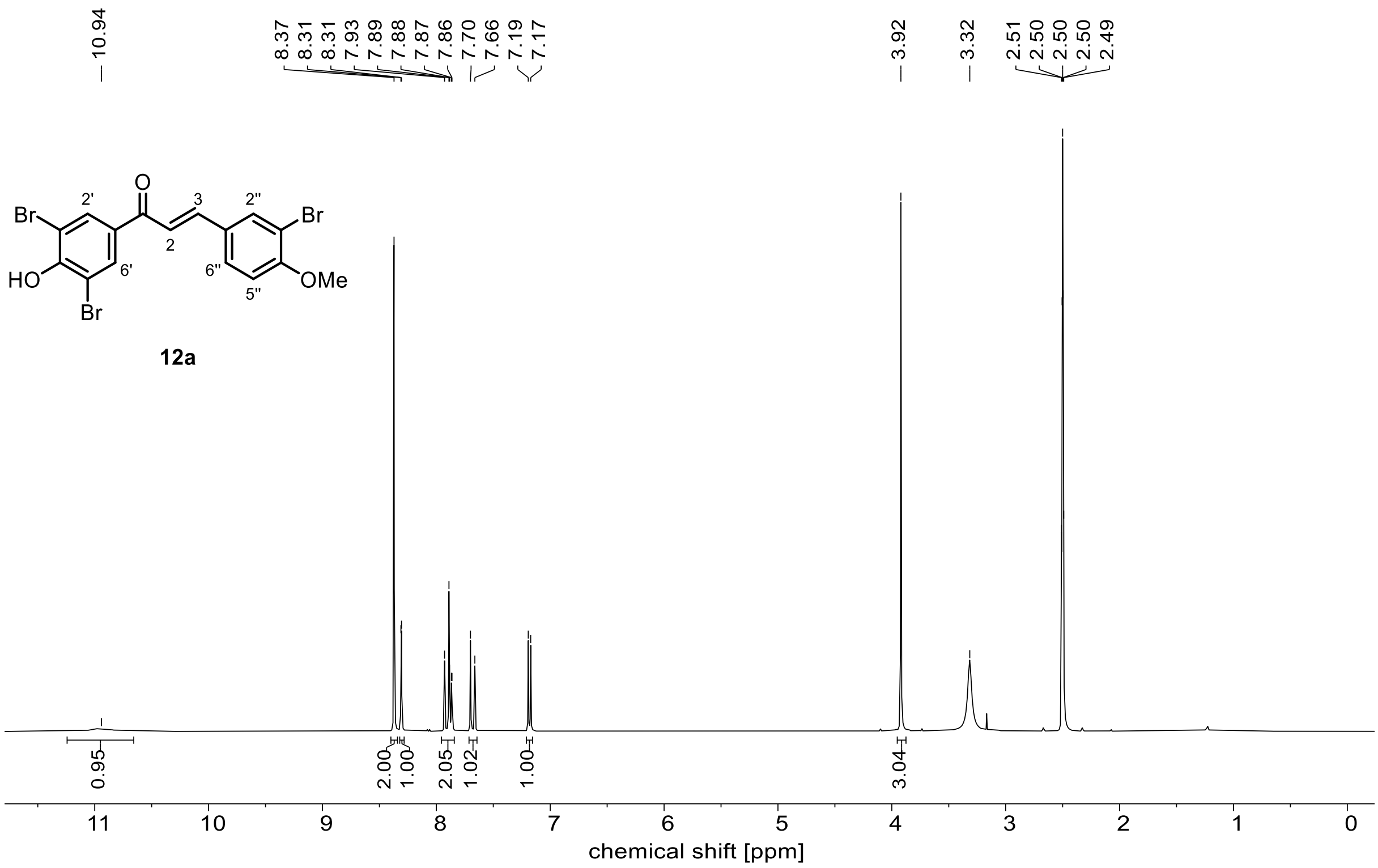

Figure S1. ^{1}H-NMR spectrum (400 MHz, DMSO-d_6) of chalcone derivative **12a**.

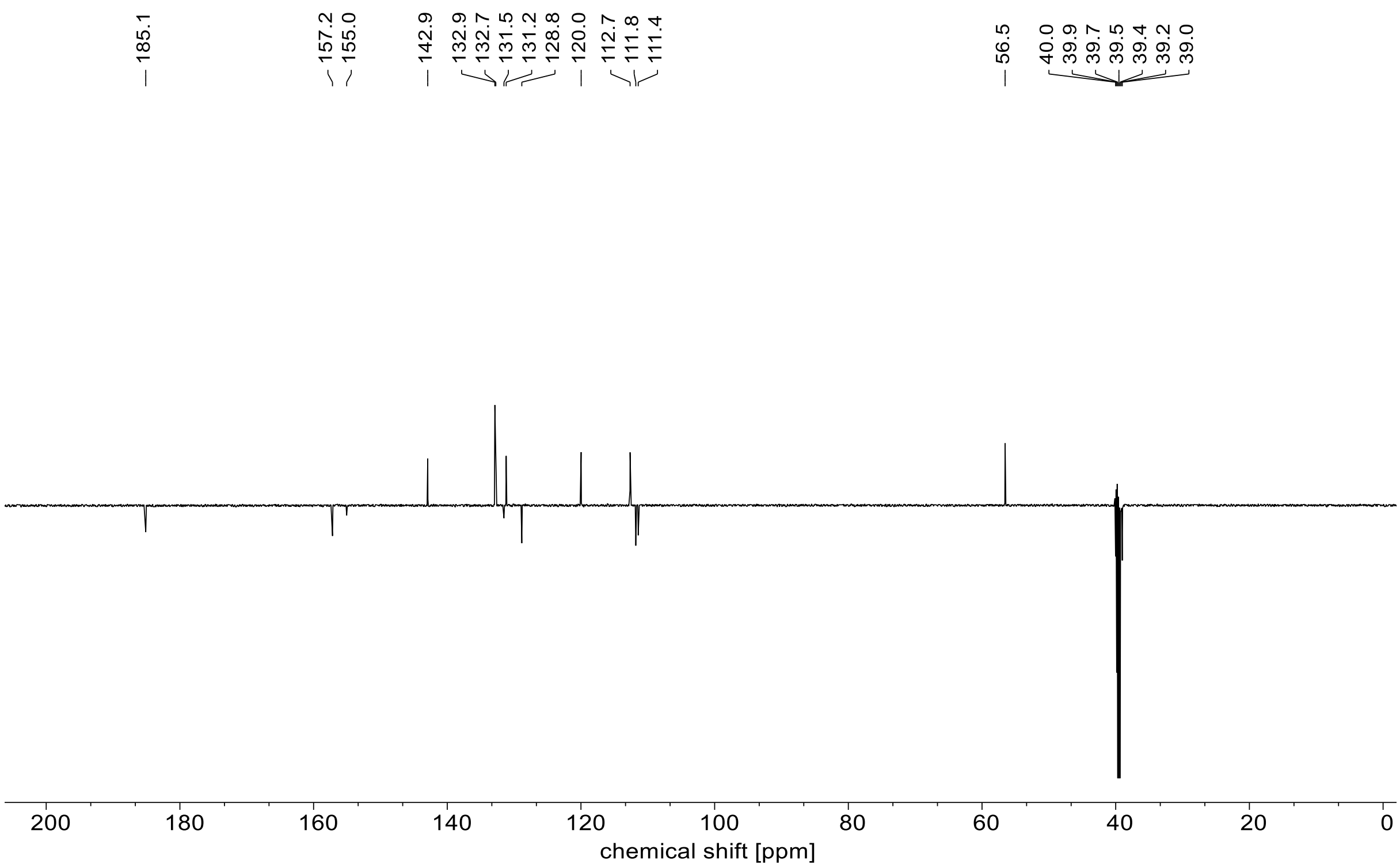

Figure S2. ^{13}C-NMR (APT) spectrum (126 MHz, DMSO-d_6) of chalcone derivative **12a**.

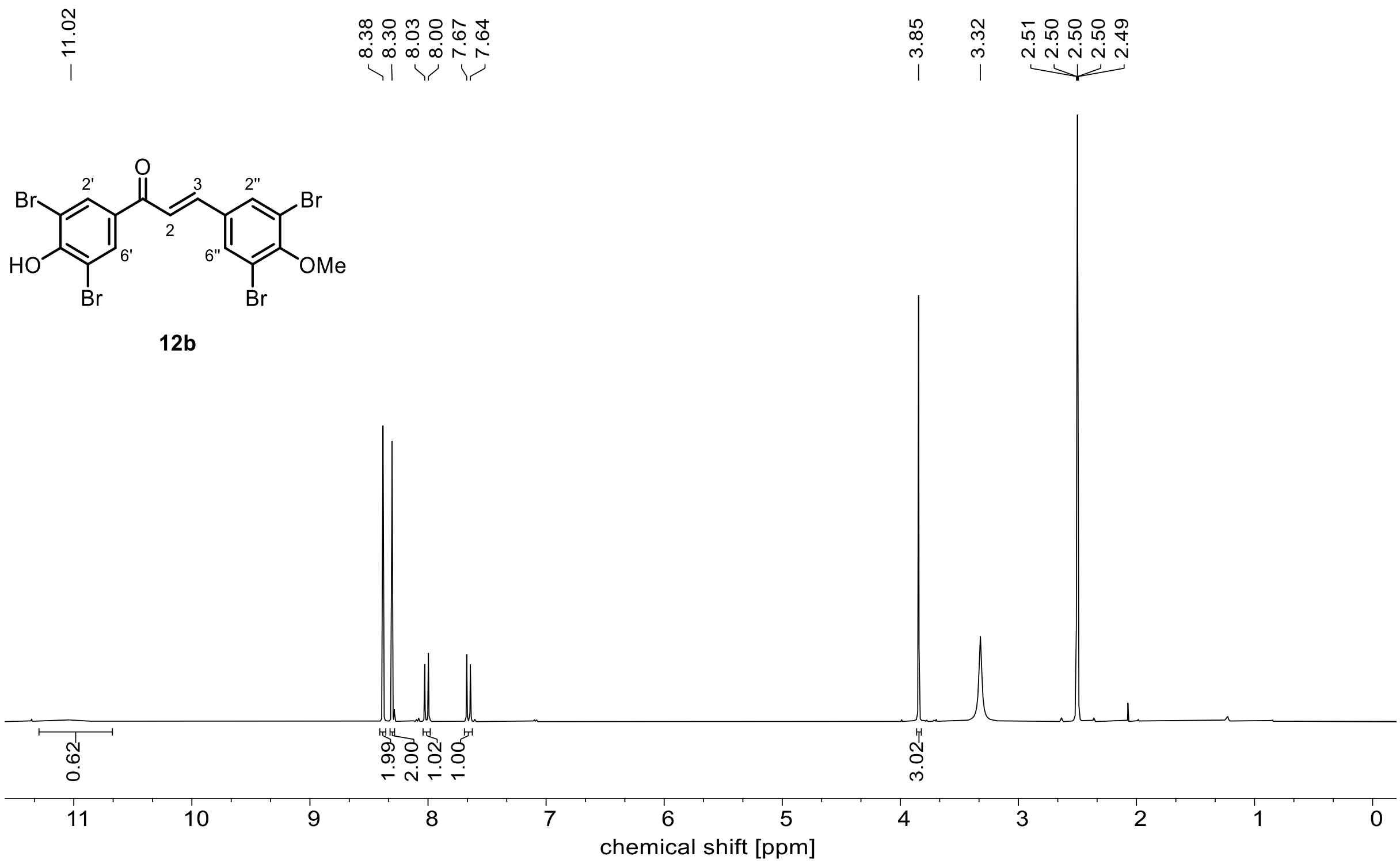

Figure S3. ^{1}H-NMR spectrum (500 MHz, DMSO-d_6) of chalcone derivative **12b**.

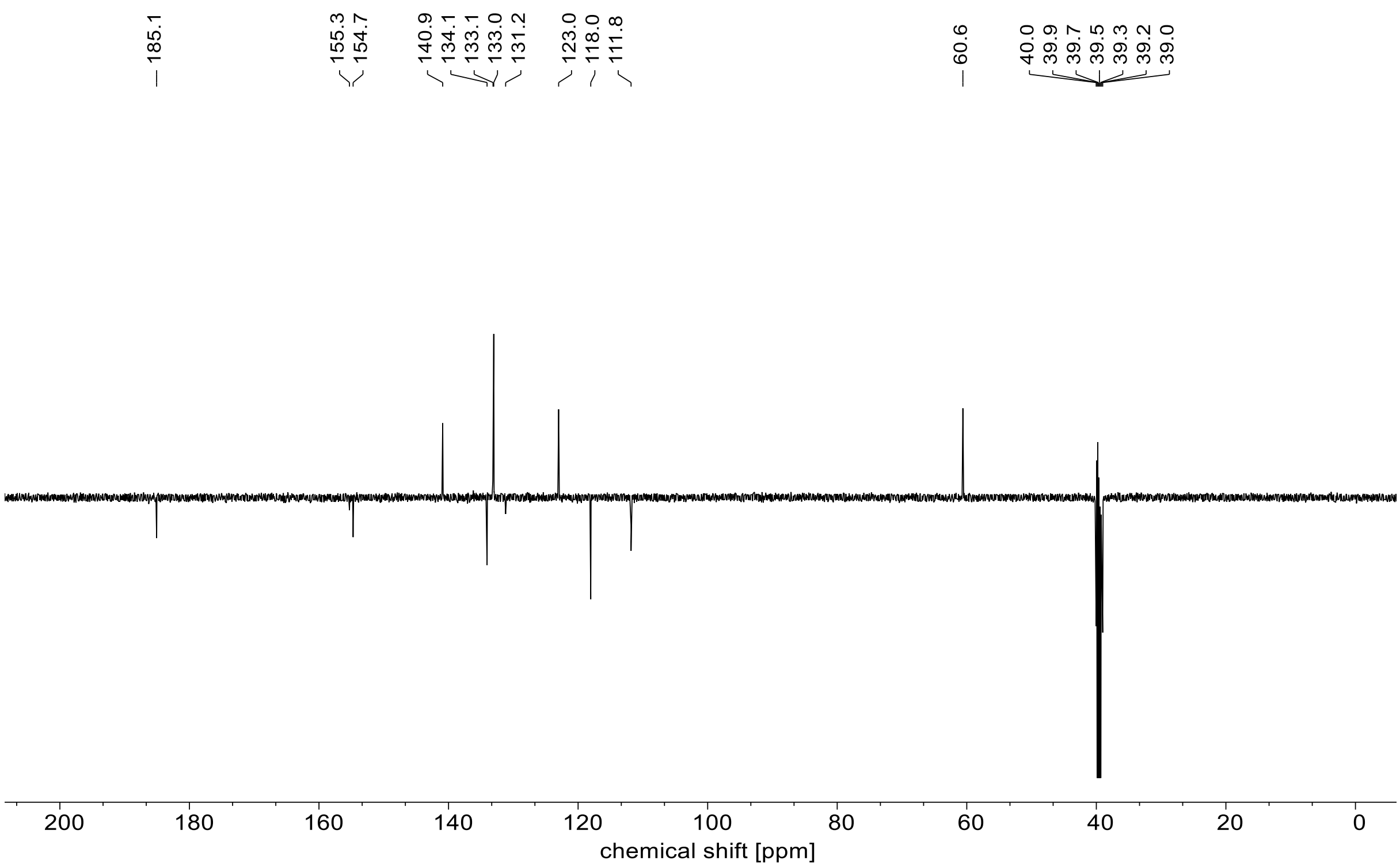

Figure S4. ^{13}C-NMR (APT) spectrum (126 MHz, DMSO-d_6) of chalcone derivative **12b**.

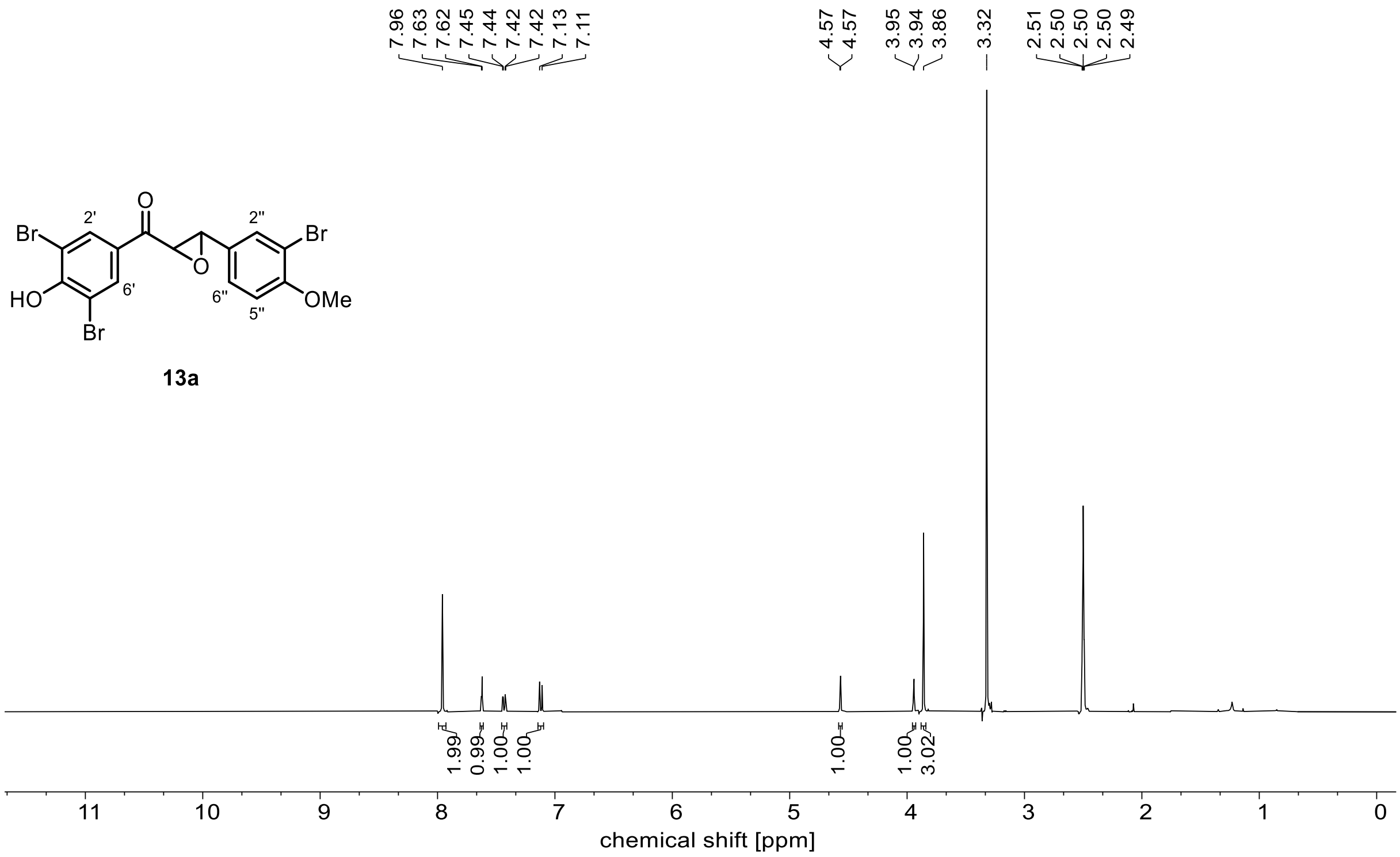

Figure S5. ^{1}H-NMR spectrum (400 MHz, DMSO-d_6) of epoxide **13a**.

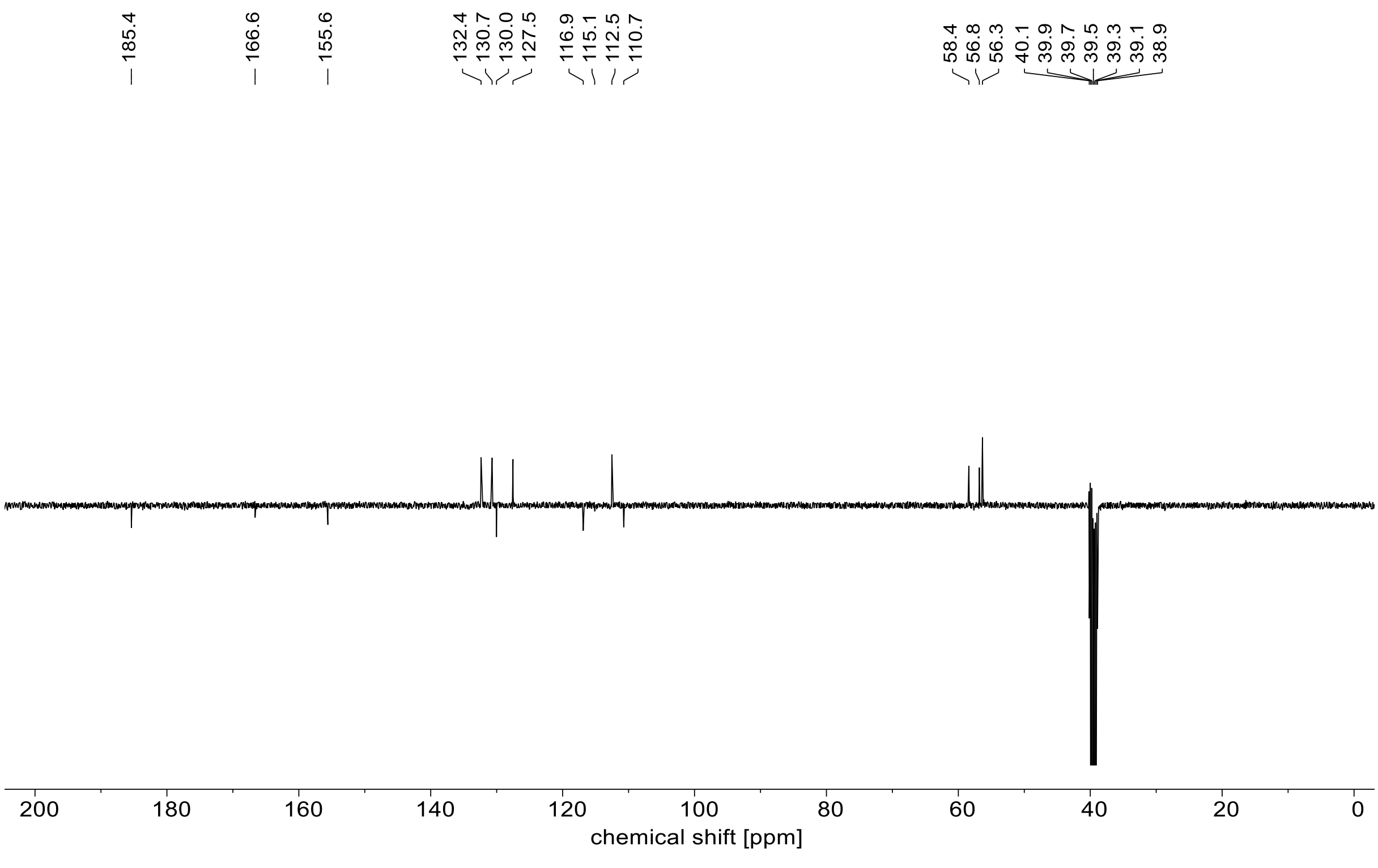

Figure S6. ^{13}C-NMR (APT) spectrum (101 MHz, DMSO-d_6) of epoxide **13a**.

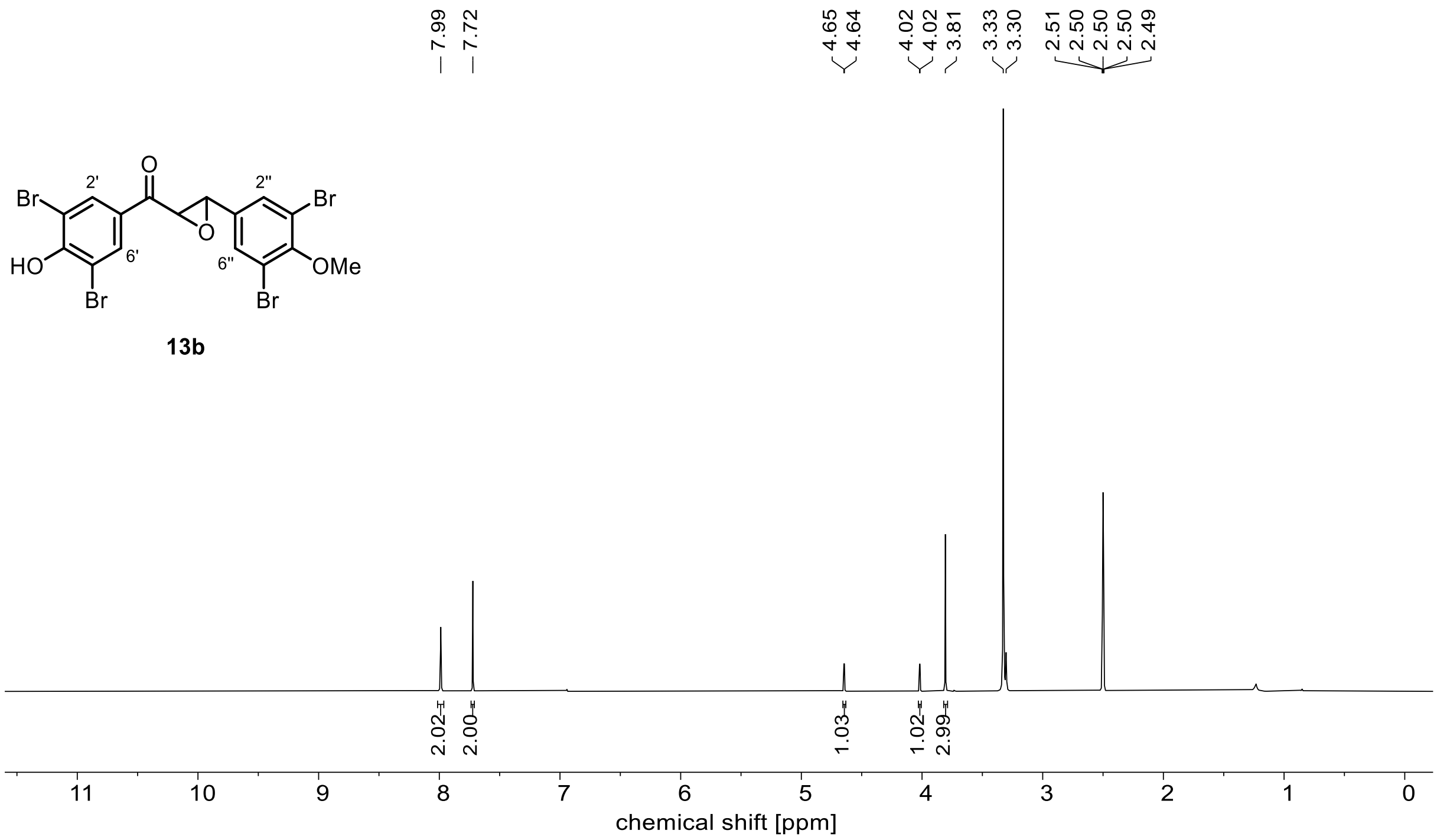

Figure S7. ^{1}H-NMR spectrum (500 MHz, DMSO-d_6) of epoxide **13b**.

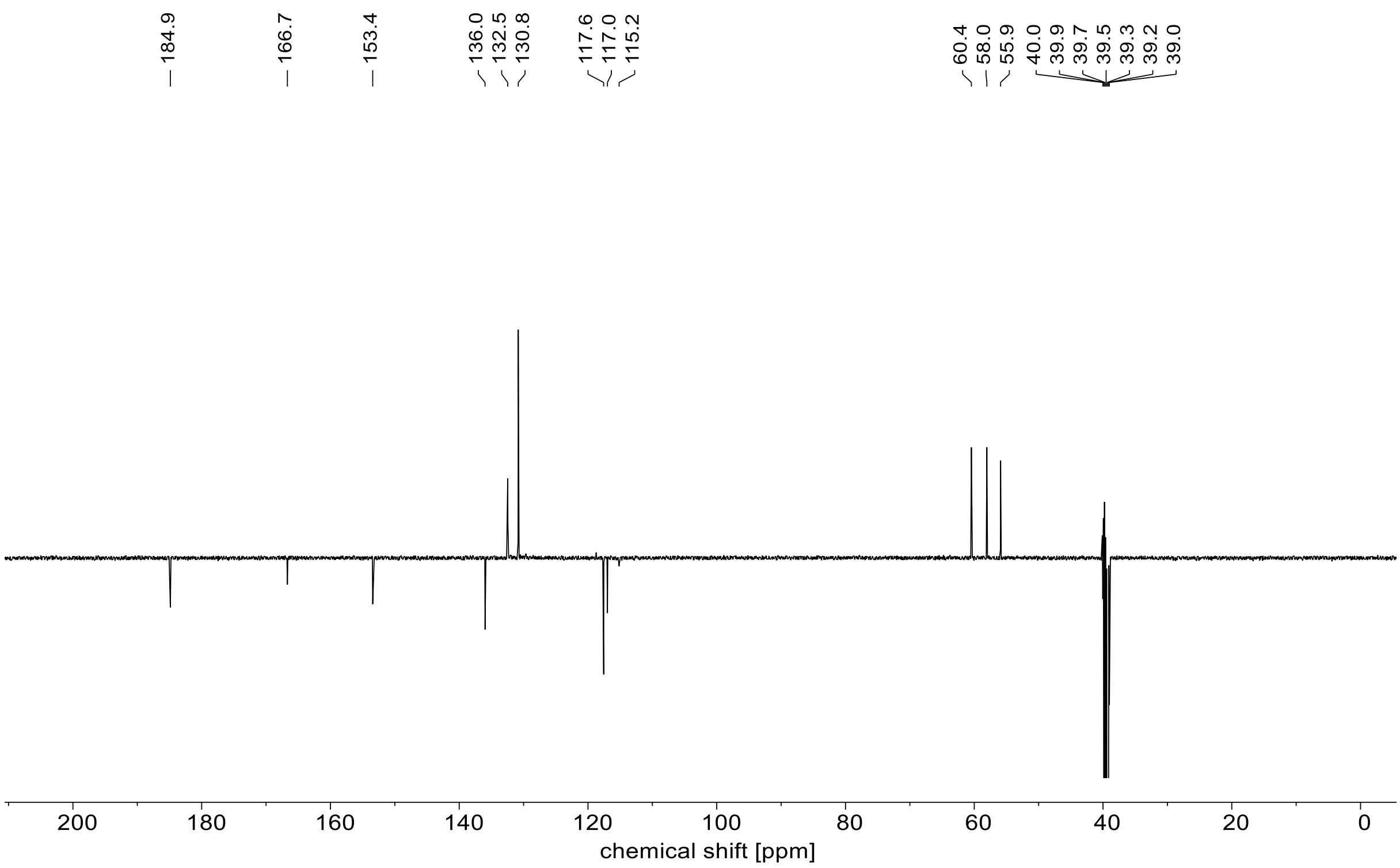

Figure S8. ^{13}C-NMR (APT) spectrum (126 MHz, DMSO-d_6) of epoxide **13b**.

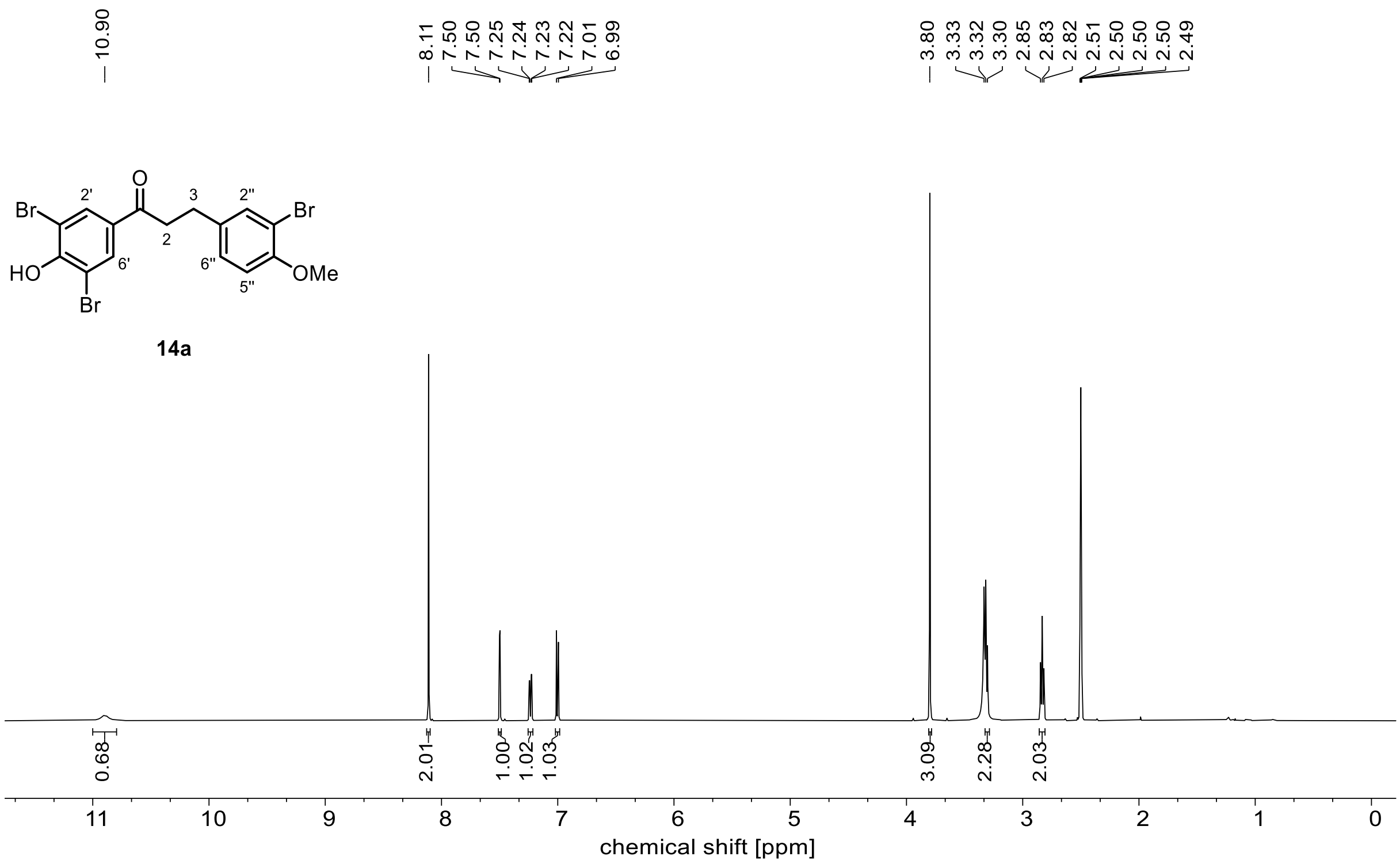

Figure S9. ^{1}H-NMR spectrum (500 MHz, DMSO-d_6) of hydrogenated derivative **14a**.

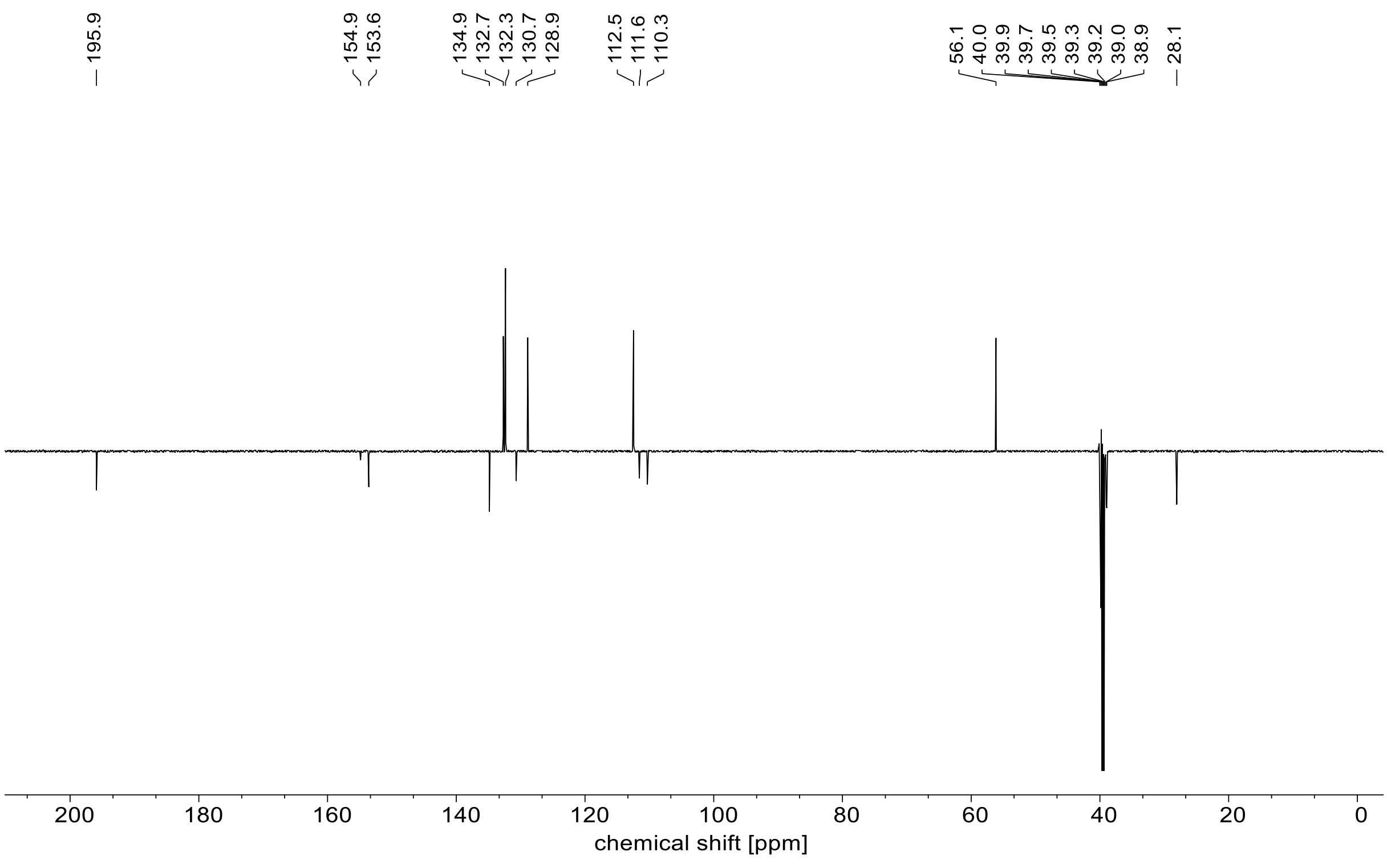

Figure S10. ^{13}C-NMR (APT) spectrum (126 MHz, DMSO-d_6) of hydrogenated derivative **14a**.

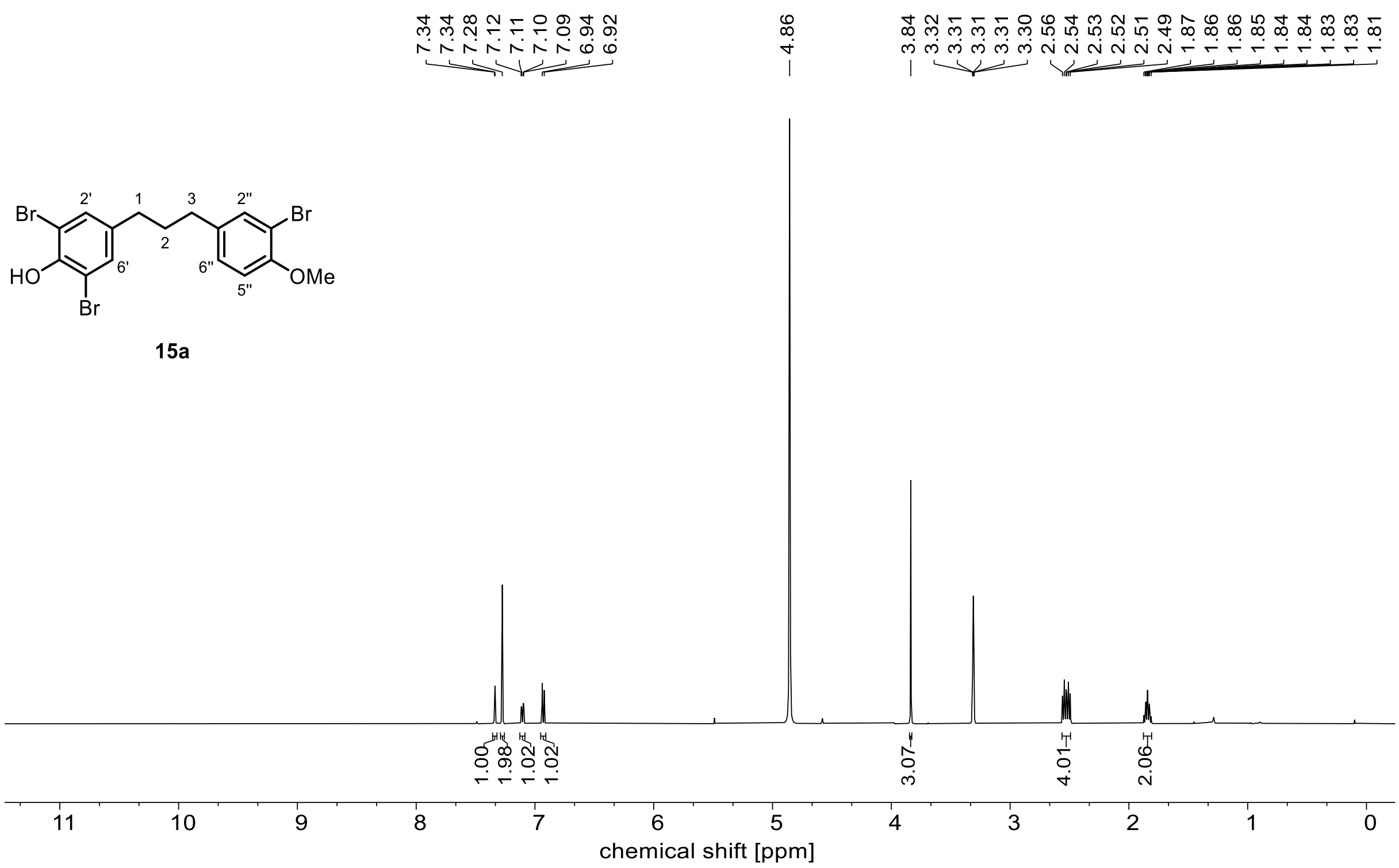

Figure S11. ^{1}H-NMR spectrum (500 MHz, methanol-d_4) of hydrogenated by-product **15a**.

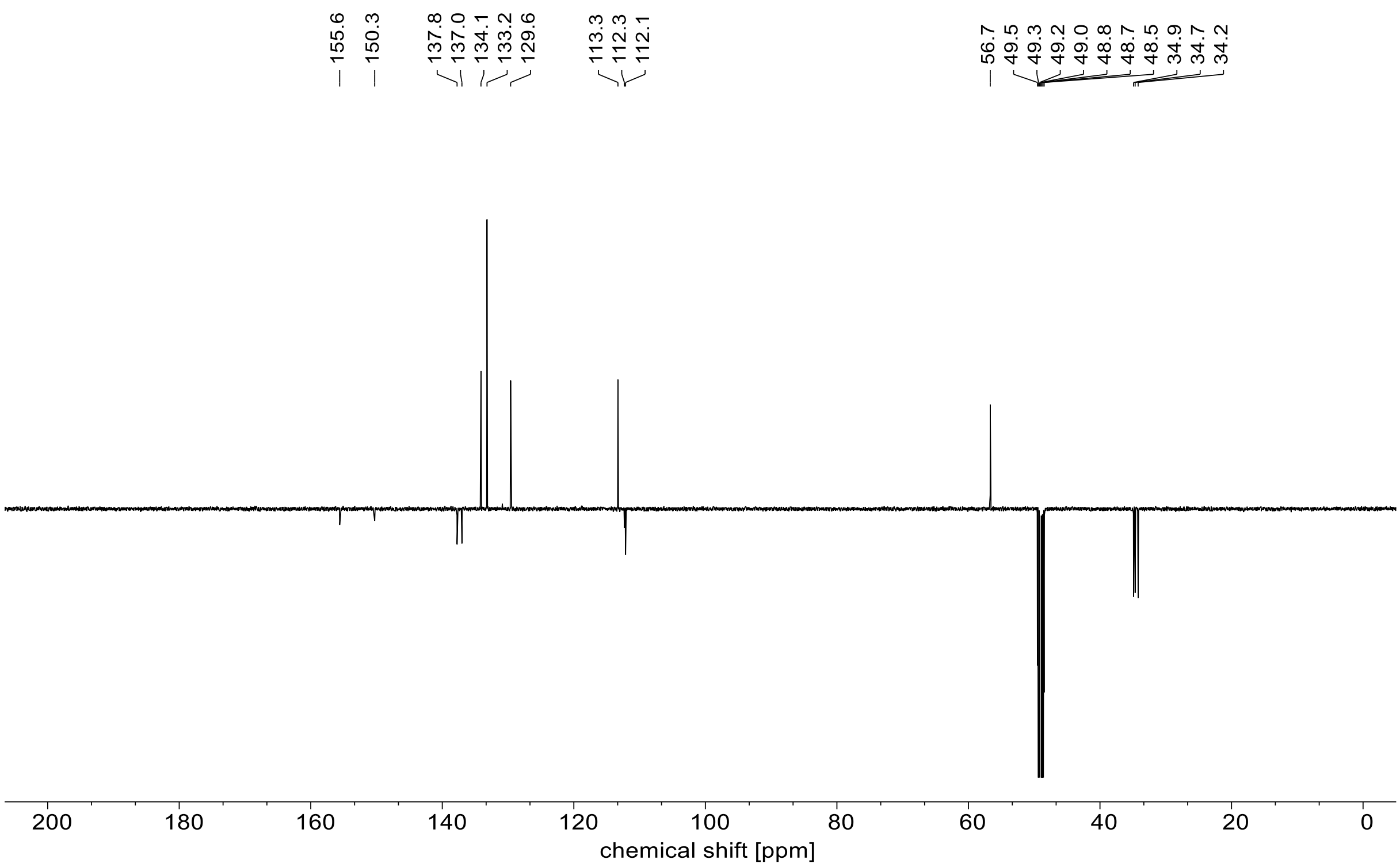

Figure S12. ^{13}C-NMR (APT) spectrum (126 MHz, methanol-d_4) of hydrogenated by-product **15a**.

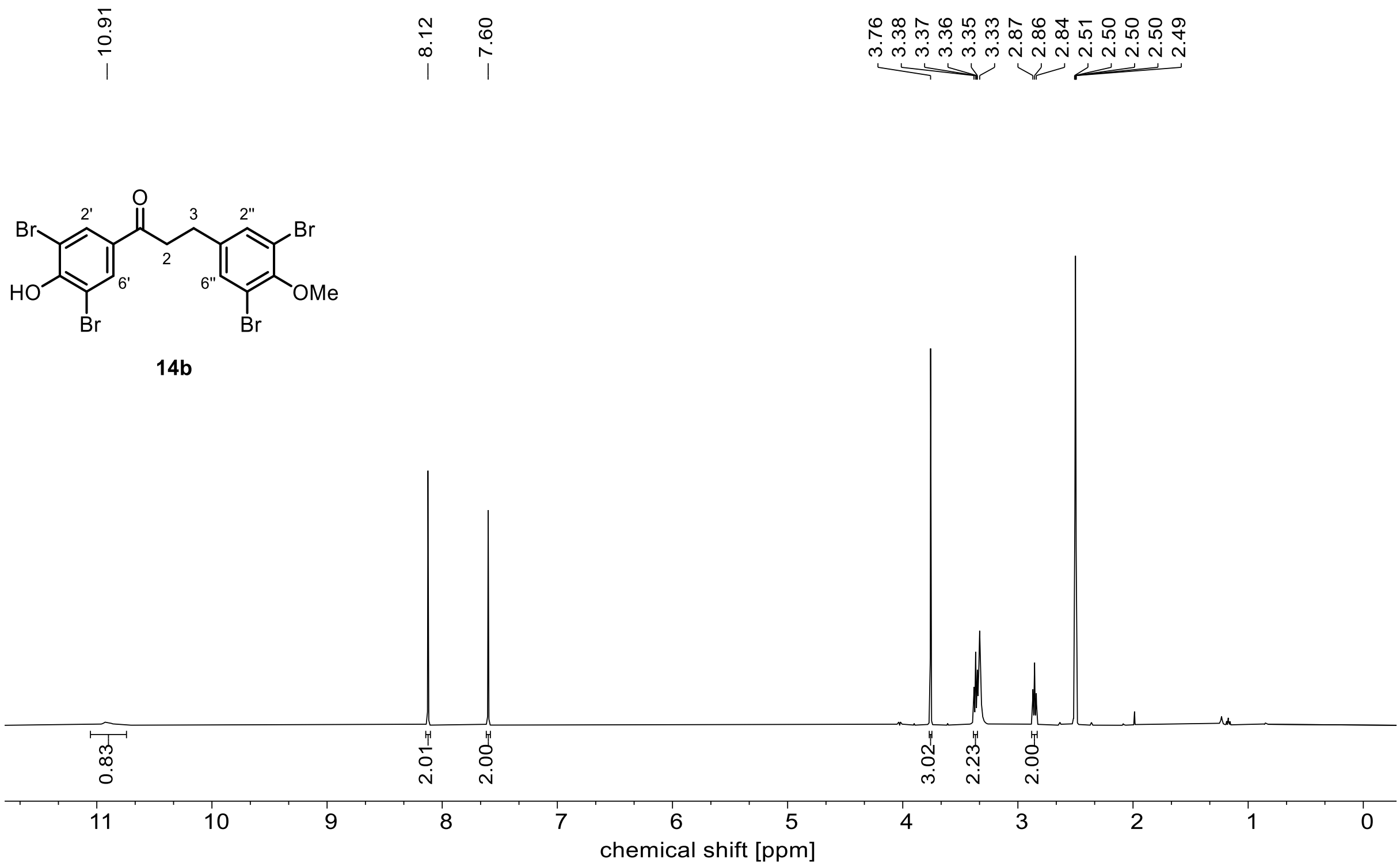

Figure S13. ^{1}H-NMR spectrum (500 MHz, DMSO-d_6) of hydrogenated derivative **14b**.

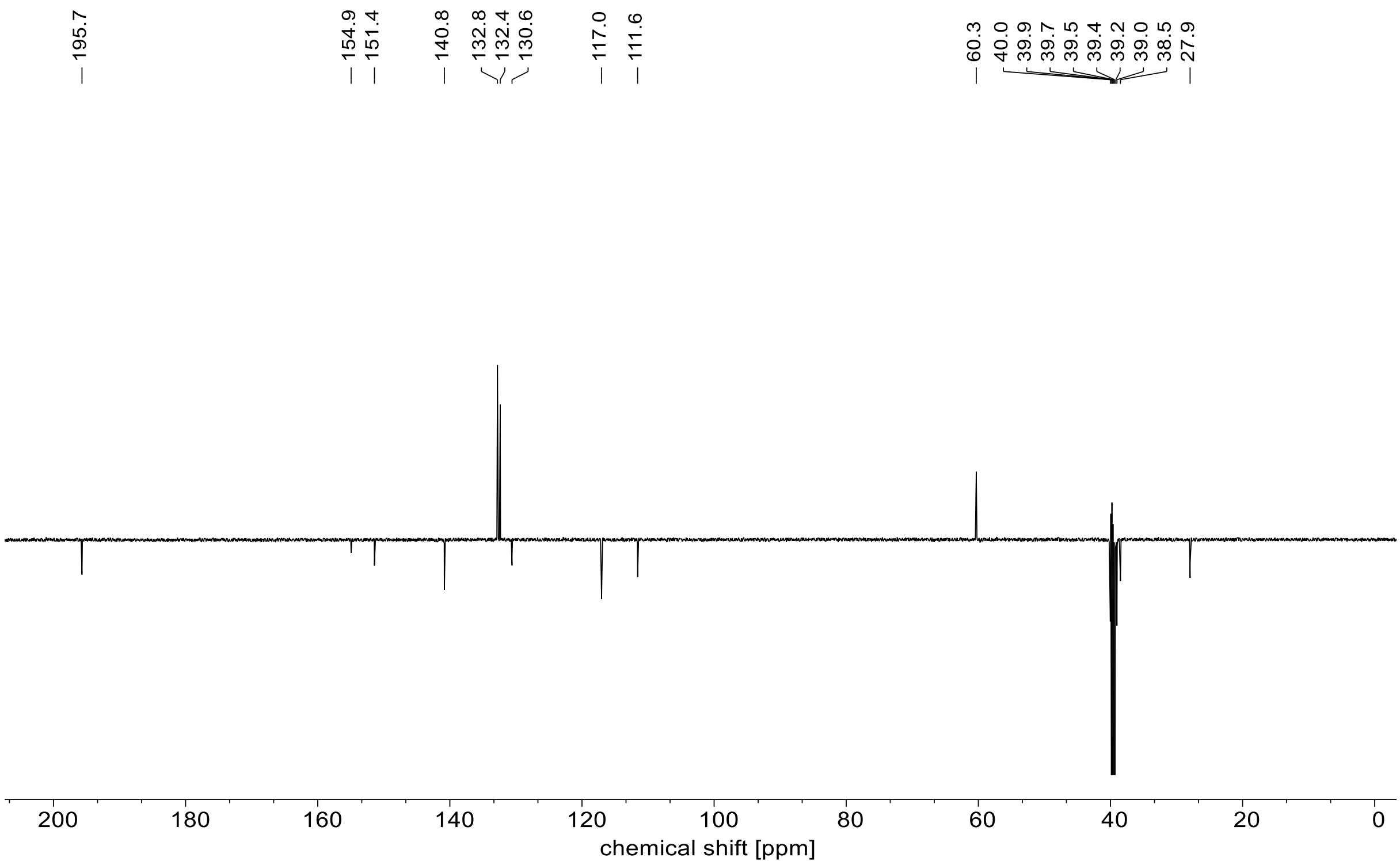

Figure S14. ^{13}C-NMR (APT) spectrum (126 MHz, DMSO-d_6) of hydrogenated derivative **14b**.

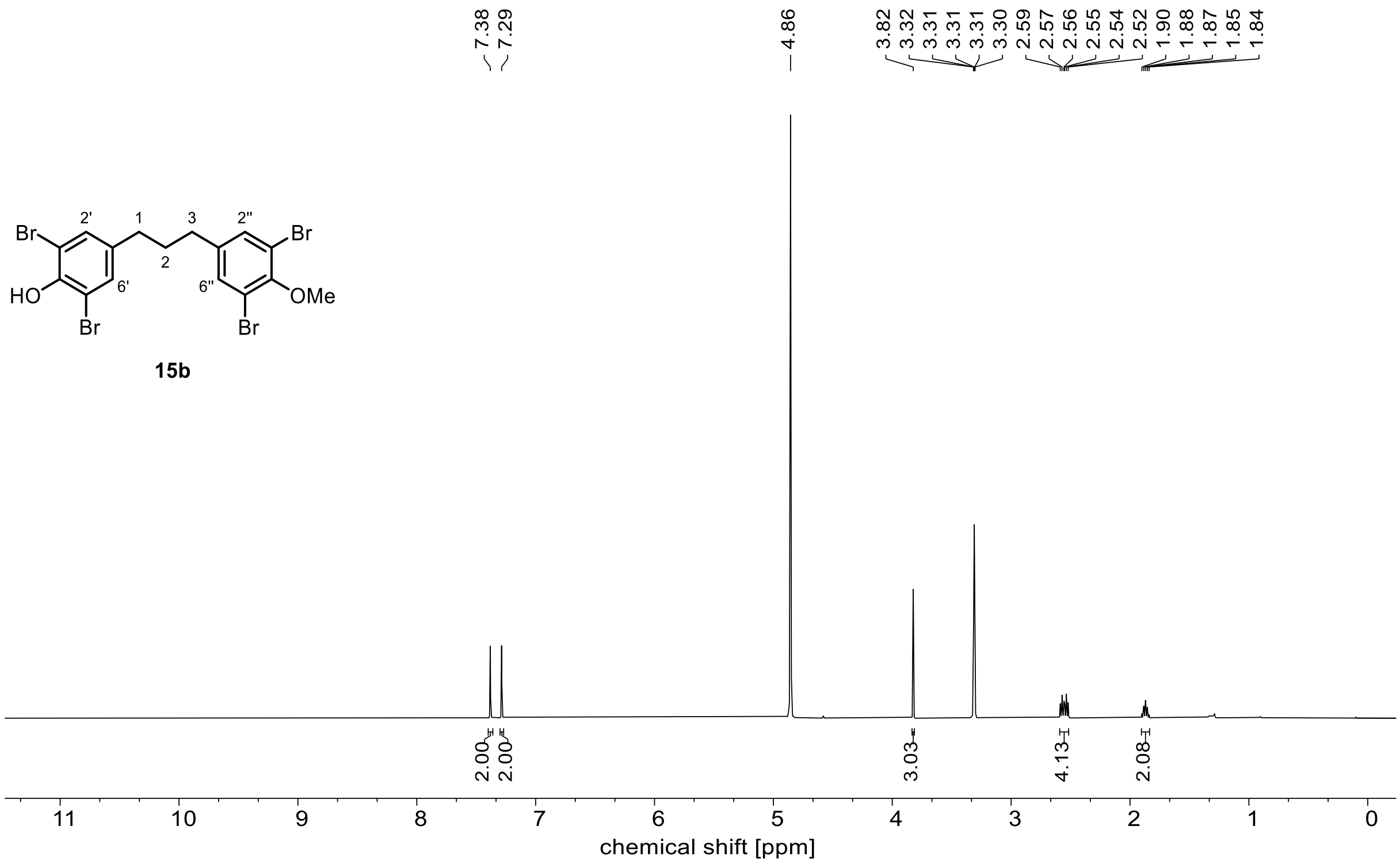

Figure S15. ^{1}H-NMR spectrum (500 MHz, methanol-d_4) of hydrogenated by-product **15b**.

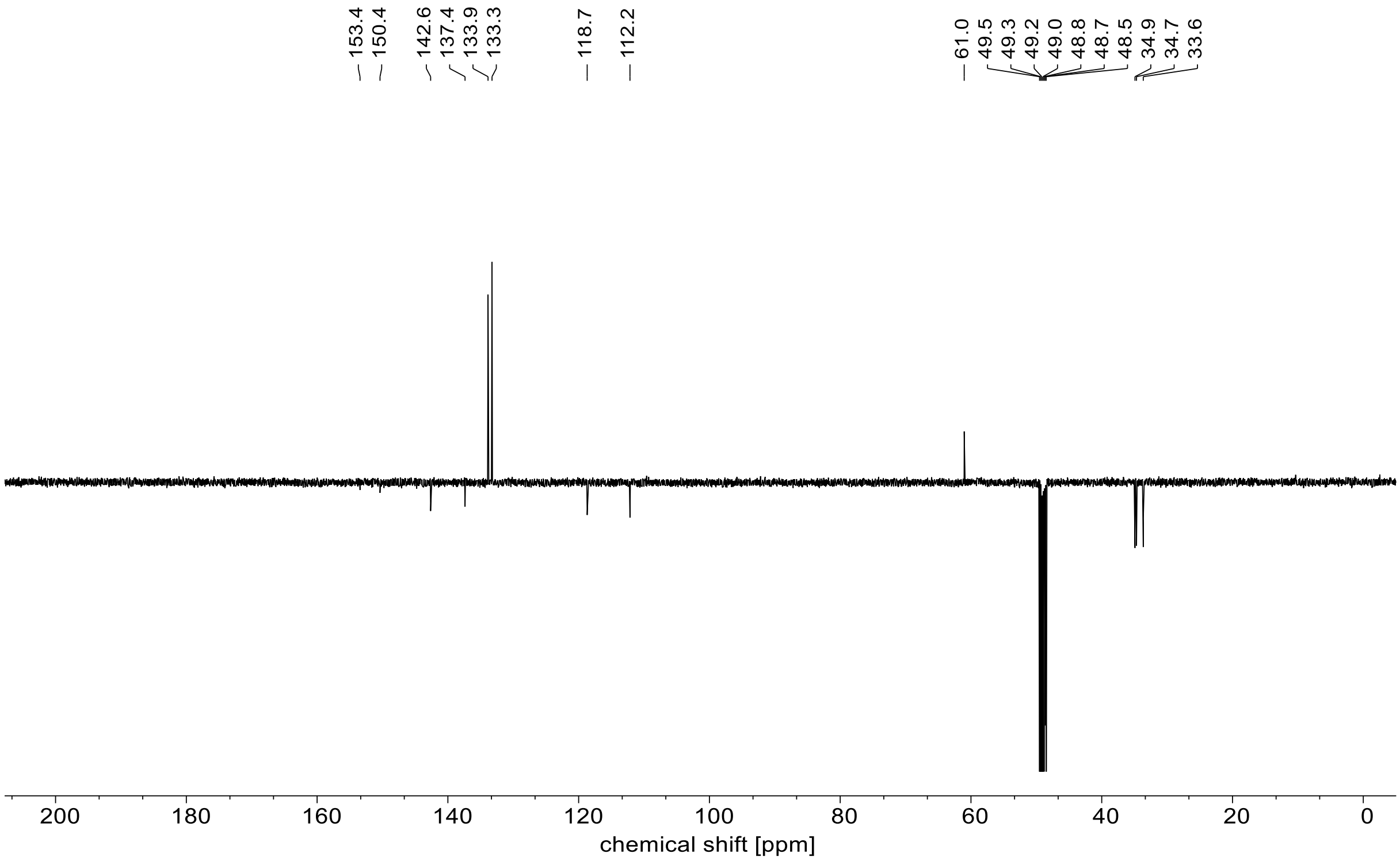

Figure S16. ^{13}C-NMR (APT) spectrum (126 MHz, methanol-d_4) of hydrogenated by-product **15b**.

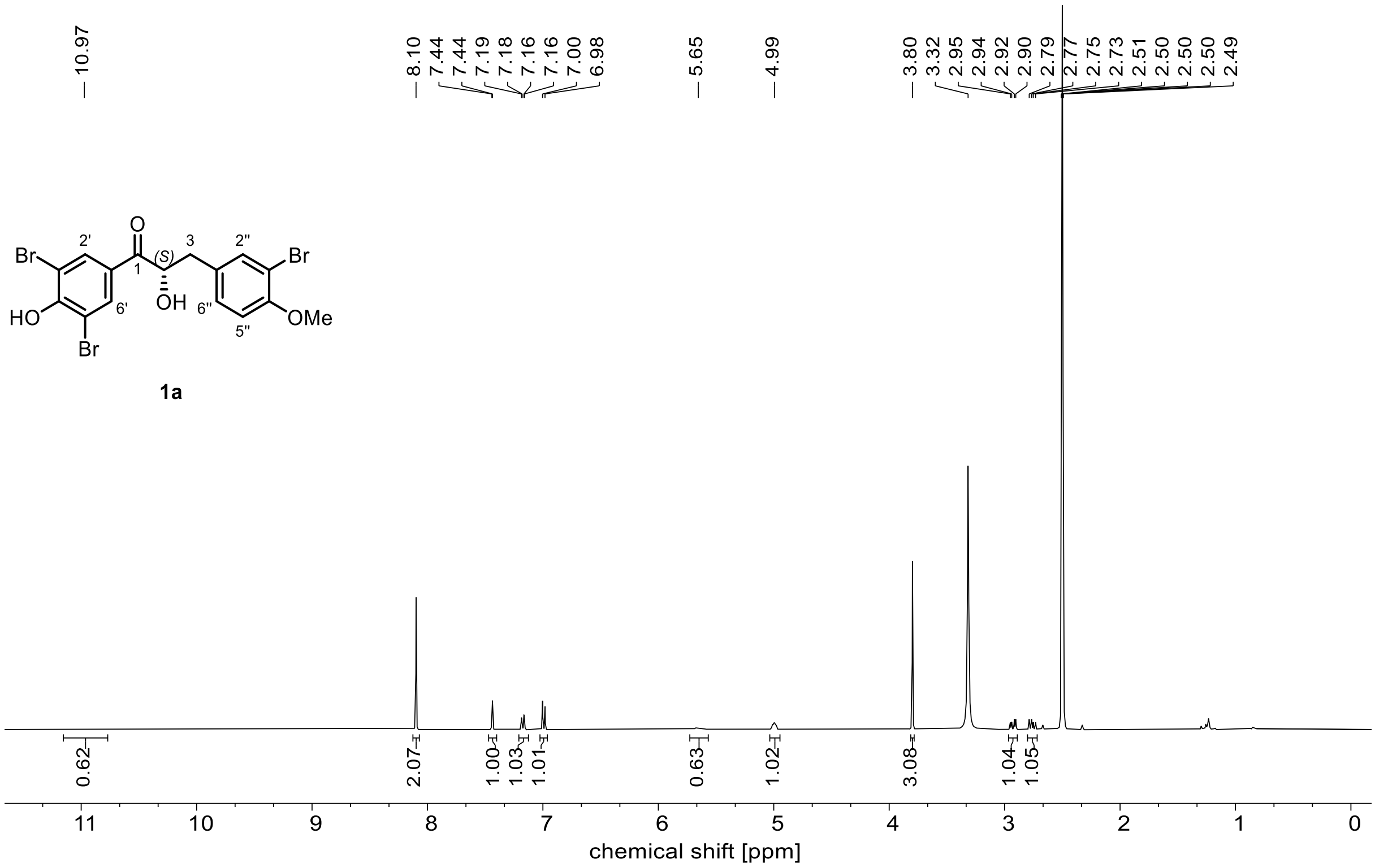

Figure S17. ¹H-NMR spectrum (400 MHz, DMSO-d_6) of procerone A **1a**.

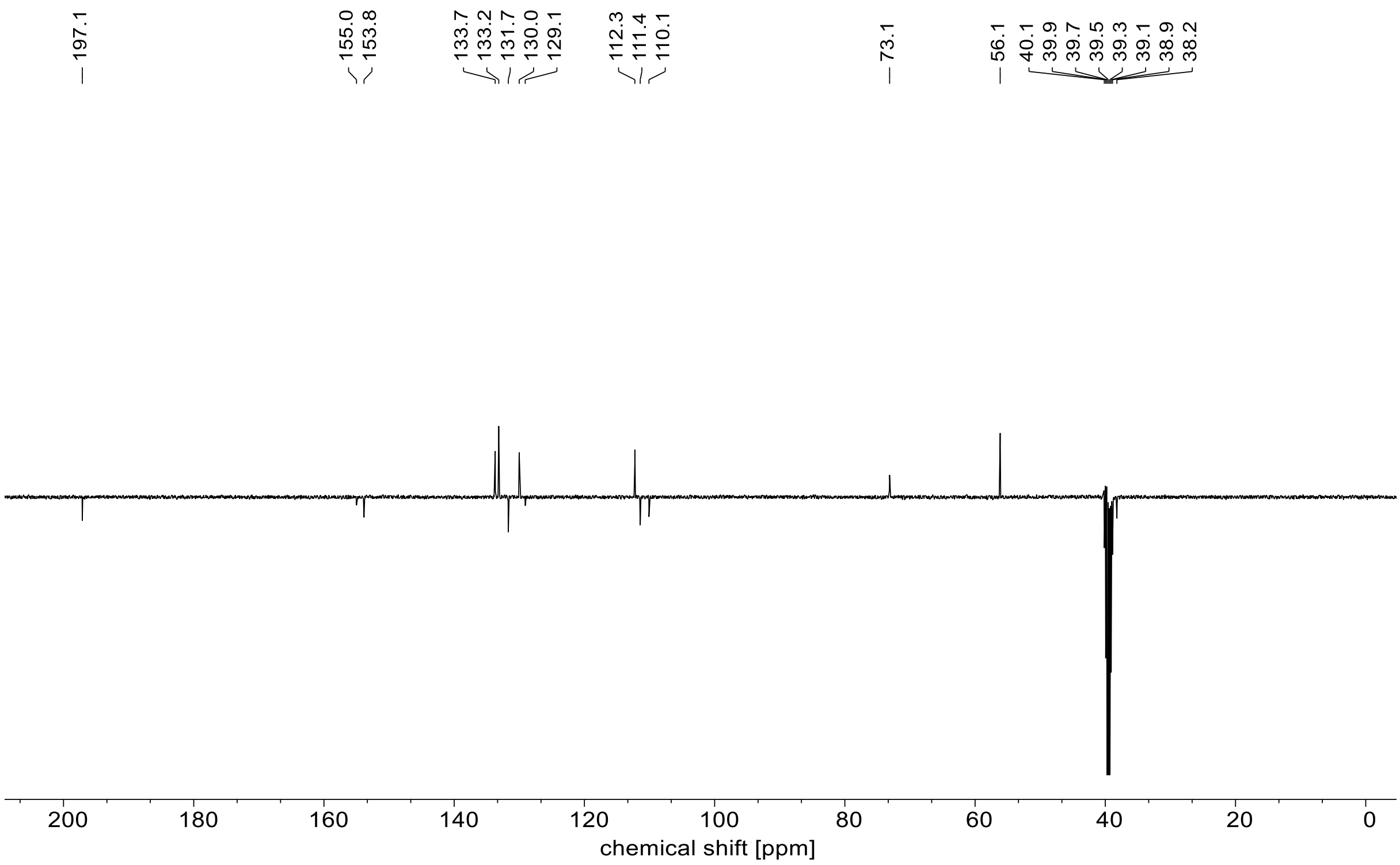

Figure S18. ¹³C-NMR (APT) spectrum (101 MHz, DMSO-d_6) of procerone A **1a**.

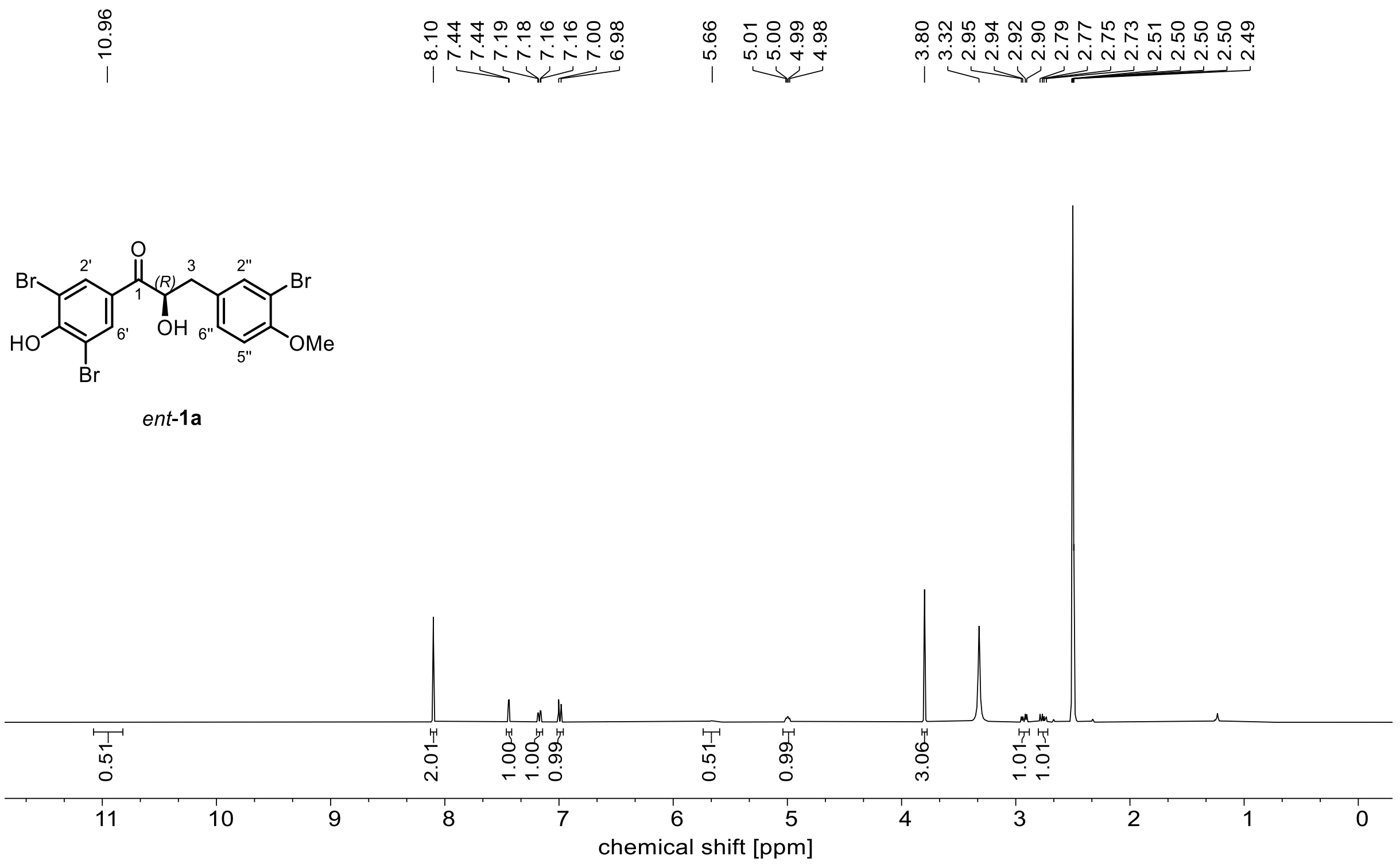

Figure S19. ^{1}H-NMR spectrum (400 MHz, DMSO-d_6) of procerone *ent*-**1a**.

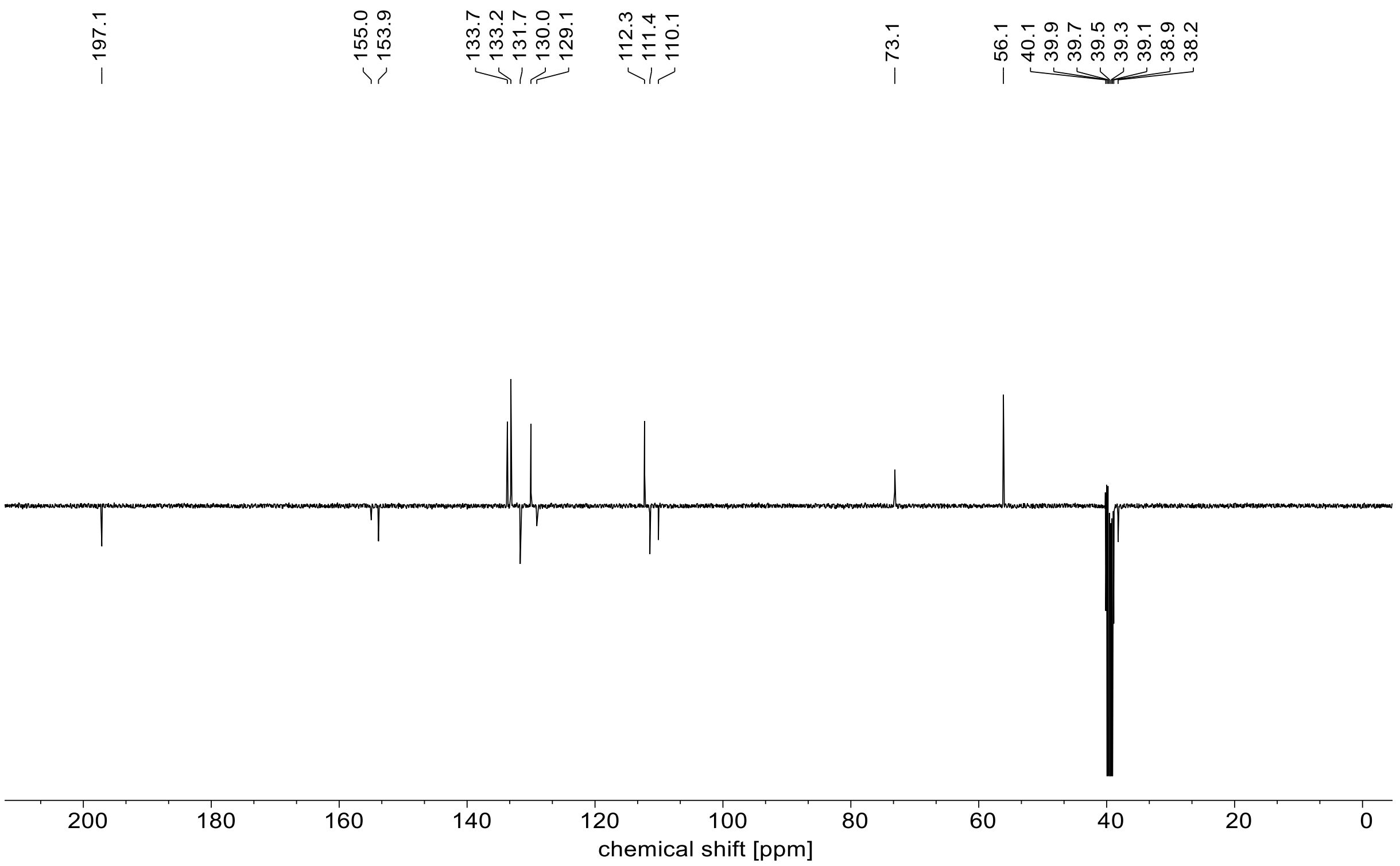

Figure S20. ^{13}C-NMR (APT) spectrum (101 MHz, DMSO-d_6) of procerone *ent*-**1a**.

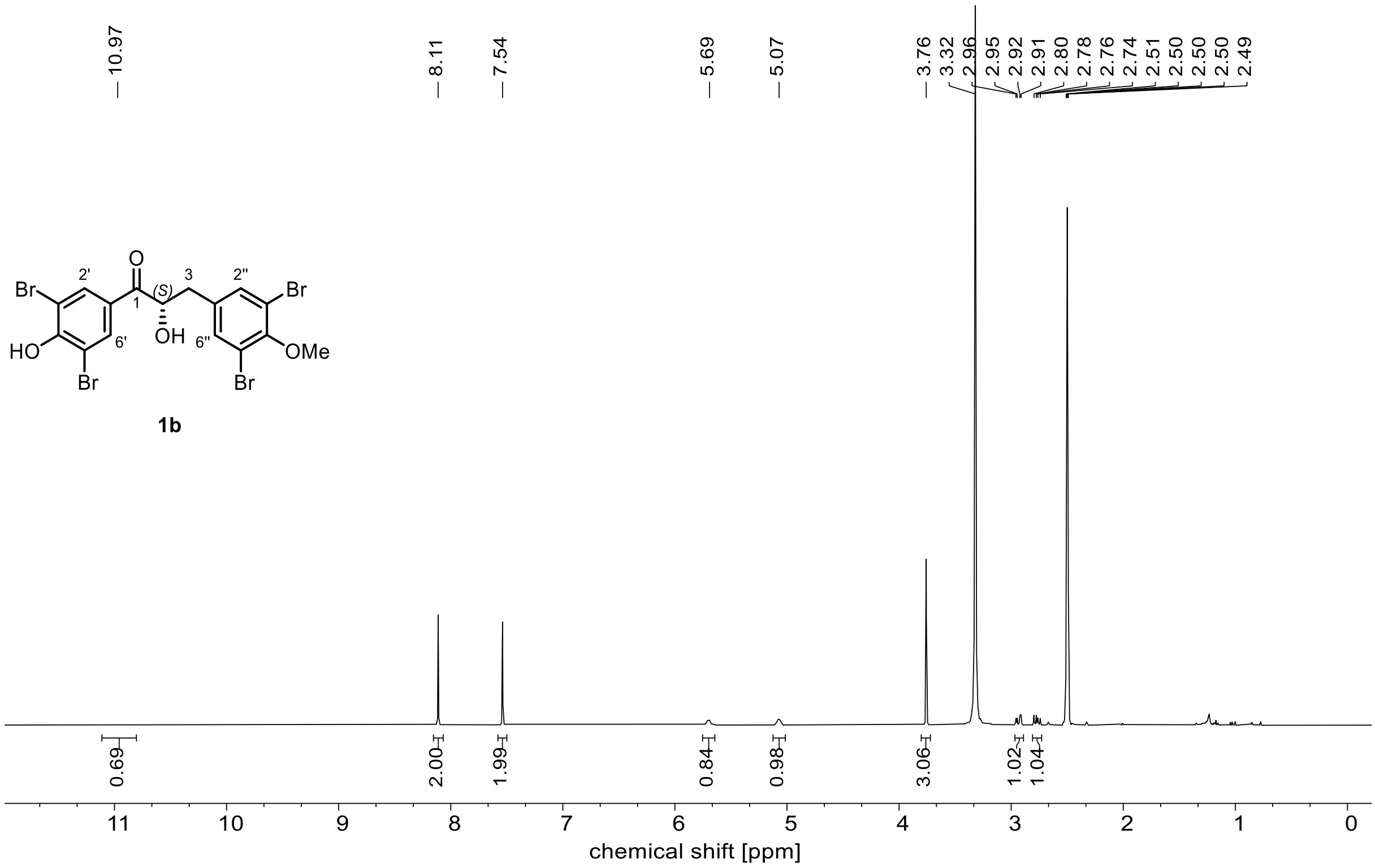

Figure S21. ^{1}H-NMR spectrum (400 MHz, DMSO-d_6) of procerone B **1b**.

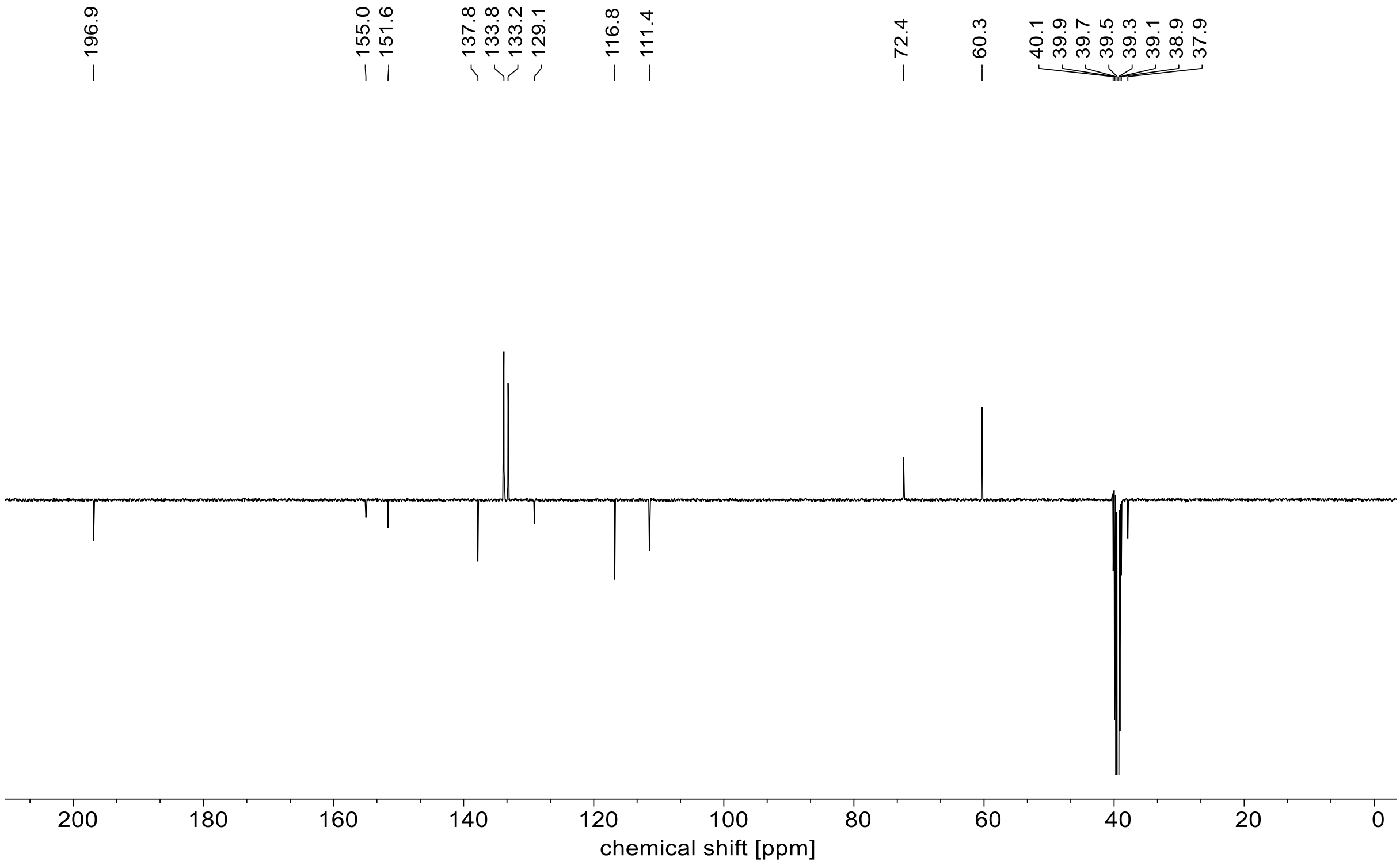

Figure S22. ^{13}C-NMR (APT) spectrum (101 MHz, DMSO-d_6) of procerone B **1b**.

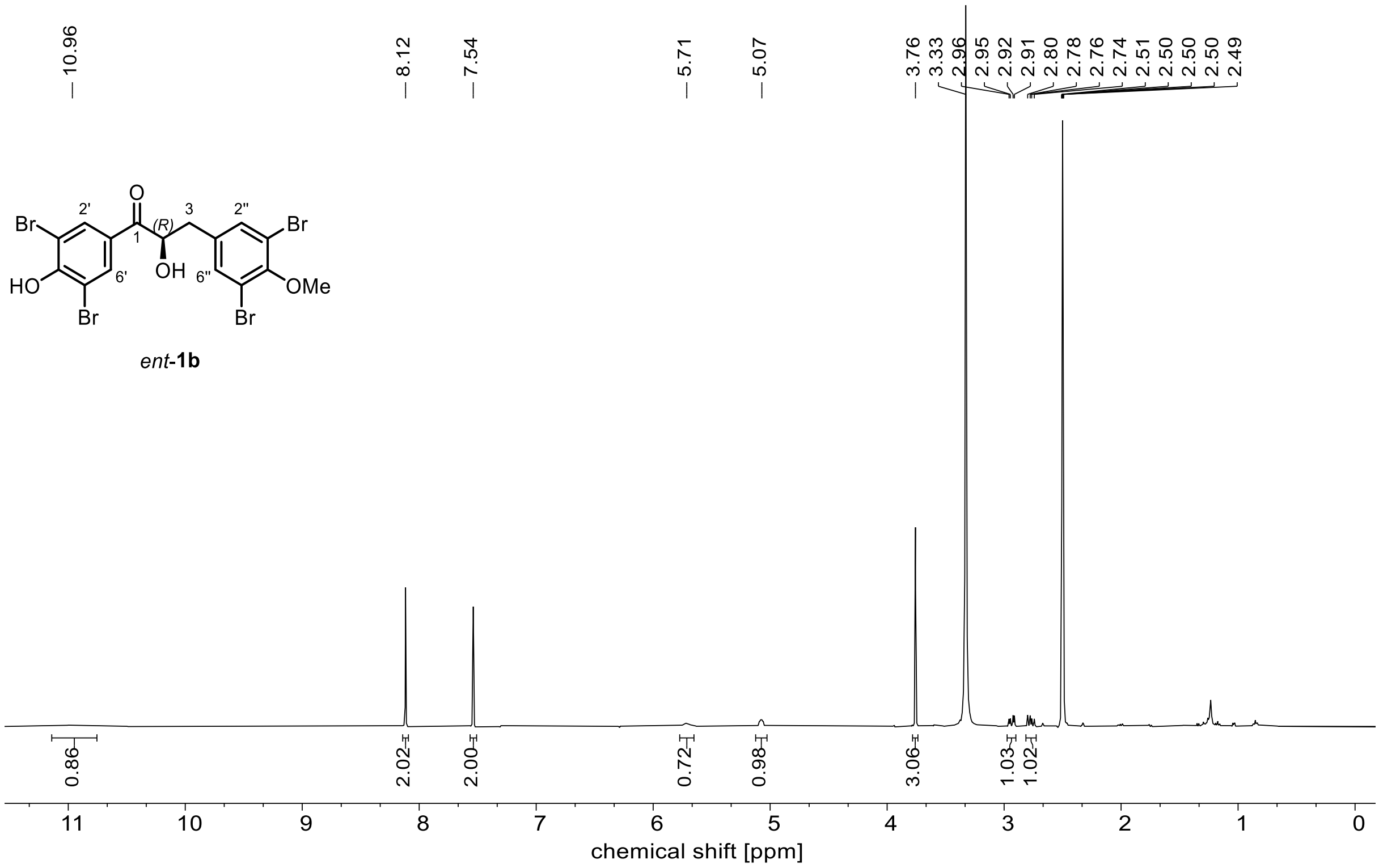

Figure S23. ^{1}H-NMR spectrum (400 MHz, DMSO-d_6) of procerone *ent*-**1b**.

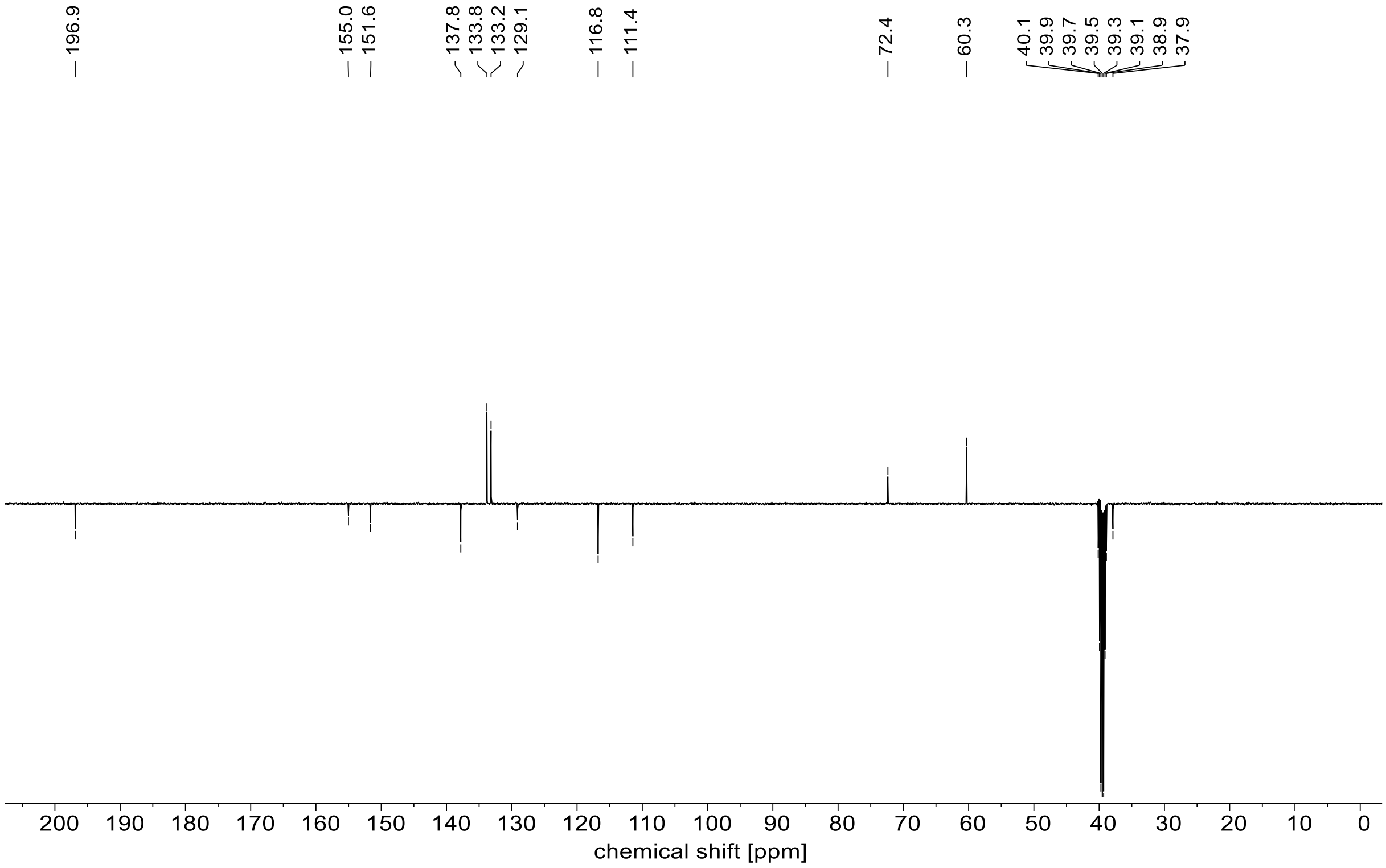

Figure S24. ^{13}C-NMR (APT) spectrum (101 MHz, DMSO-d_6) of procerone *ent*-**1b**.

4 Screening/Optimization

4.1 Aldol condensation

Screening experiments were performed with 50 mg of 3,5-dibromo-4-hydroxyacetophenone **10** under microwave irradiation.

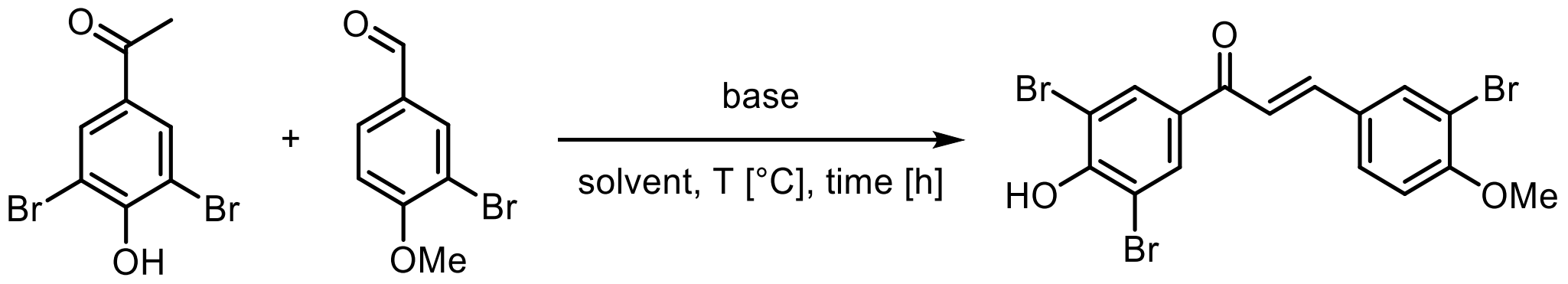

10 **11a** **12a**

Table S1. Screening results of the Aldol condensation between ketone **10** (1.0 equiv.) and aldehyde **11a** (1.0 equiv.).

Entry	Solvent	Base (equiv.)	Additives	*c* [mol/L][a]	T [°C]	Time [h]	Ratio of **10**/**12a**[a]
1	MeOH	20% aq. NaOH (4.0)	-	0.085	80	3	1.5:1
2	EtOH	20% aq. NaOH (4.0)	-	0.085	80	3	1:1
3	*i*-PrOH	20% aq. NaOH (4.0)	-	0.085	80	3	10:1
4	DMF	20% aq. NaOH (4.0)	-	0.085	80	3	5:1
5	THF	20% aq. NaOH (4.0)	-	0.085	80	3	1.3:1
6	2-MeTHF	20% aq. NaOH (4.0)	-	0.085	80	3	4:1
7	1,4-Dioxane	20% aq. NaOH (4.0)	-	0.085	80	3	1:1.3
8	MeCN	20% aq. NaOH (4.0)	-	0.085	80	3	8:1
9	CH_2Cl_2	20% aq. NaOH (4.0)	-	0.085	80	3	1:0
10	DMSO	20% aq. NaOH (4.0)	-	0.085	80	3	24:1
11	-	20% aq. NaOH (4.0)	TBABr	0.085	110	3	17:1
12	MeOH	$LiOH\cdot H_2O$ (4.0)	-	0.085	80	3	1:2.3
13	MeOH	NaOH (4.0)	-	0.085	80	3	1:1.3
14	MeOH	KOH (4.0)	-	0.085	80	3	1.4:1
15	MeOH	$CsOH\cdot H_2O$ (4.0)	-	0.085	80	3	1:1.3
16	MeOH	$Ca(OH)_2$ (4.0)	-	0.085	80	3	1:1.3
17	MeOH	$Mg(OH)_2$ (4.0)	-	0.085	80	3	1:0
18	EtOH	$LiOH\cdot H_2O$ (4.0)	-	0.085	80	3	1:2.5
19	EtOH	NaOH (4.0)	-	0.085	80	3	1:1
20	EtOH	KOH (4.0)	-	0.085	80	3	1:1.6
21	Pyridine	-	-	0.567	55	3	1:0
22[c]	THF	LDA[d] (4.0)	-	0.085	−78	1	-
23	MeOH	$LiOH\cdot H_2O$ (4.0)	-	0.085	40	3	6:1
24	MeOH	$LiOH\cdot H_2O$ (4.0)	-	0.085	60	3	1.3:1
25	MeOH	$LiOH\cdot H_2O$ (4.0)	-	0.085	90	3	1:3.2
26	MeOH	$LiOH\cdot H_2O$ (4.0)	-	0.085	100	3	1:3.6
27	MeOH	$LiOH\cdot H_2O$ (4.0)	-	0.085	100	6	1:3
28	MeOH	$LiOH\cdot H_2O$ (4.0)	-	0.085	110	3	1:3.6
29	MeOH	$LiOH\cdot H_2O$ (4.0)	-	0.085	120	3	1:2
30	MeOH	$LiOH\cdot H_2O$ (2.0)	-	0.085	100	3	1:1.4

Entry	Solvent	Base (equiv.)	Additives	*c* [mol/L]	T [°C]	Time [h]	Ratio of **10**/**12a**[b]
31	MeOH	$LiOH{\cdot}H_2O$ (3.0)	-	0.085	100	3	1:2
32	MeOH	$LiOH{\cdot}H_2O$ (5.0)	-	0.085	100	3	1:3
33	MeOH	$LiOH{\cdot}H_2O$ (6.0)	-	0.085	100	3	1:2.5
34	MeOH	$LiOH{\cdot}H_2O$ (7.0)	-	0.085	100	3	1:2.1
35[c]	MeOH	$LiOH{\cdot}H_2O$ (4.0)	-	0.085	55	6	1.2:1
36[c]	MeOH	$LiOH{\cdot}H_2O$ (4.0)	$MgSO_4$	0.085	55	6	1.3:1
37[c]	MeOH	$LiOH{\cdot}H_2O$ (4.0)	Molecular sieves (4 Å)	0.085	55	6	1.4:1
38	MeOH	$LiOH{\cdot}H_2O$ (4.0)	-	0.284	55	6	1:7.7
39	MeOH	$LiOH{\cdot}H_2O$ (4.0)	-	0.284	100	6	1:2
40	MeOH	$LiOH{\cdot}H_2O$ (4.0)	-	0.284	55	1	1:2.5
41	MeOH	$LiOH{\cdot}H_2O$ (4.0)	-	0.284	55	3	1:7.1
42	MeOH	$LiOH{\cdot}H_2O$ (4.0)	-	0.284	55	8	1:7.7
43	MeOH	$LiOH{\cdot}H_2O$ (4.0)	-	0.567	55	3	1:16

(a) The concentration of the reaction was also an important parameter and is therefore listed. (b) Determined by ^{1}H-NMR analysis of the crude mixture. (c) Reaction was not performed under microwave irradiation. (d) LDA was prepared *in situ* with *n*-butyllithium (1.6 M) and diisopropylamine.

4.2 Epoxide ring-opening

All ring-opening reactions were performed with epoxide **13a** as starting material.

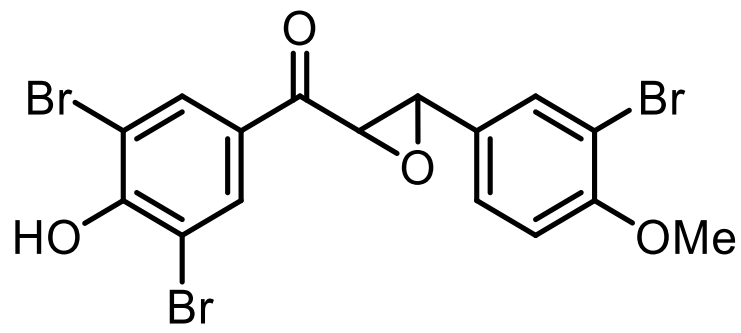

13a

Table S2: Screening results of epoxide ring-opening attempts with **13a** (1.0 equiv.).

Entry	Reaction conditions	Result
1[3,4]	H_2, Pd/C (10%)	Decomposition + dehalogenation
2[3,4]	H_2, Pd/C (5%)	Decomposition + dehalogenation
3[3]	H_2, Raney-Ni	Decomposition + ketone reduction
4[5]	$NaBH_4$	Ketone reduction
5[6]	$BH_3 \cdot SMe_2$	Ketone reduction to the alcohol + epoxide intact
6[7]	Light	β-Hydroxy ketone
7	1 M HCl	Decomposition
8[8]	Camphorsulfonic acid (MeOH/THF 1:1)	Additional methoxy-group in β-position (mixture *anti:syn* 1:1.1)
9[8]	*p*-TsOH (MeOH/THF 1:1)	Additional methoxy-group in β-position (mixture *anti:syn* 1:1.1)
10	Camphorsulfonic acid (MeCN)	Decomposition

5 HPLC

Semi-preparative separation of enantiomers was performed with a Shimadzu LC-8A preparative liquid chromatograph (Shimadzu Corporation, Tokyo, Japan) on a Chiralpak IA column (5 µm particle size; 20 mm ⌀ × 250 mm L) (Daicel Inc., Tokyo, Japan). Samples with concentrations about 10 mg/mL in tetrahydrofuran were manually injected (1 mL sample loop) at a flow rate of 18 mL/min (procerone A **1a**) or 17 mL/min (procerone B **1b**) at room temperature. As mobile phase 93.0% *n*-hexane mixed with 7.0% isopropanol acidified with 0.1% trifluoroacetic acid (TFA) was applied for procerone A **1a**/*ent*-**1a** and 91.0% *n*-hexane mixed with 9.0% isopropanol acidified with 0.1% TFA for procerone B **1b**/*ent*-**1b**. Solvents were used in HPLC grade. Fractions were collected with an FRC-10A fraction collector depending on the signal level at a wavelength of 254 nm.

Enantiopurity of the enantiomers was determined by high-performance liquid chromatography (HPLC) on a Shimadzu prominence LC-20AD liquid chromatograph device (Shimadzu Corporation, Tokyo, Japan), equipped with a Chiralpak IA column (5 µm particle size, 4.6 mm ⌀ × 250 mm L) (Daicel Inc., Tokyo, Japan). The same conditions as for the semi-preparative separation was used. Solvents were used in HPLC grade. Flow rate was set to 1.0 mL/min, injection volume was 10 µL (between 0.5 and 1 mg/mL solutions in tetrahydrofuran) and the temperature was kept at 28 °C. The purity was determined from the UV chromatogram at 254 nm as the ratio of the peak area of the enantiomer in excess minus the peak area of the second enantiomer to the total peak area of the two enantiomers. All measurements were performed in triplicate with a coefficient of variation ≤ 0.1%.

5.1 Procerone A 1a/*ent*-1a

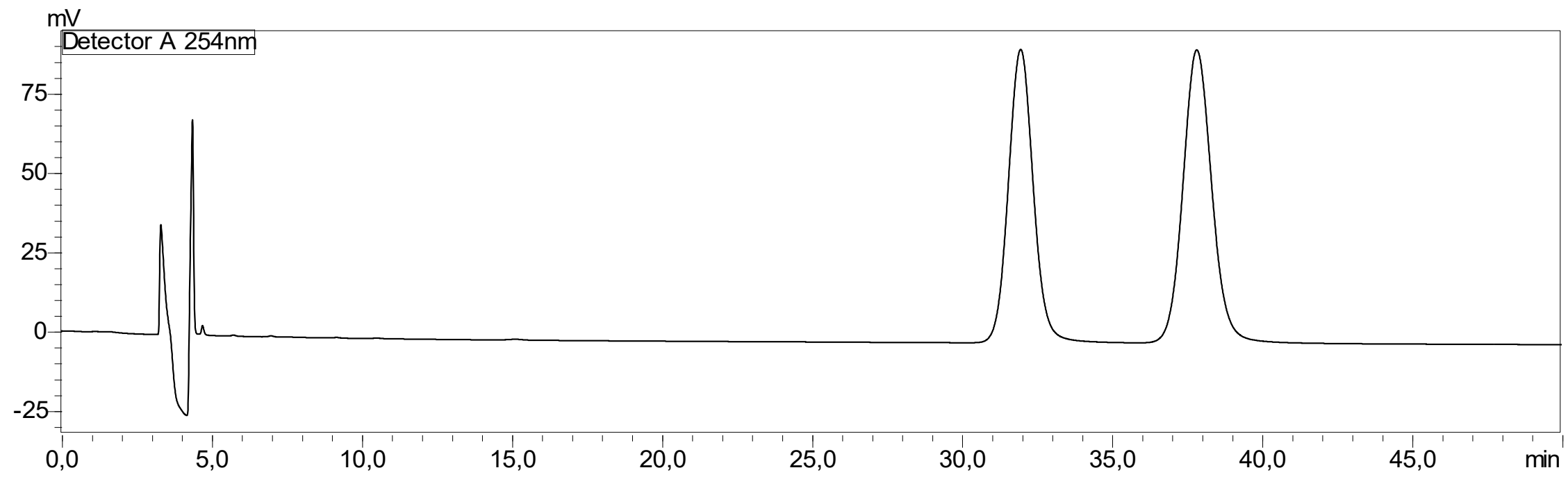

Figure S25. HPLC chromatogram of the reference of procerone A **1a**/*ent*-**1a**.

Table S3. HPLC reference data for enantio-purity measurements of procerone A **1a** and *ent*-**1a**.

Compound	Ret. time [min]	Peak area	Area [%]
Reference	32.0	5316347	46.241
	37.8	6180627	53.759

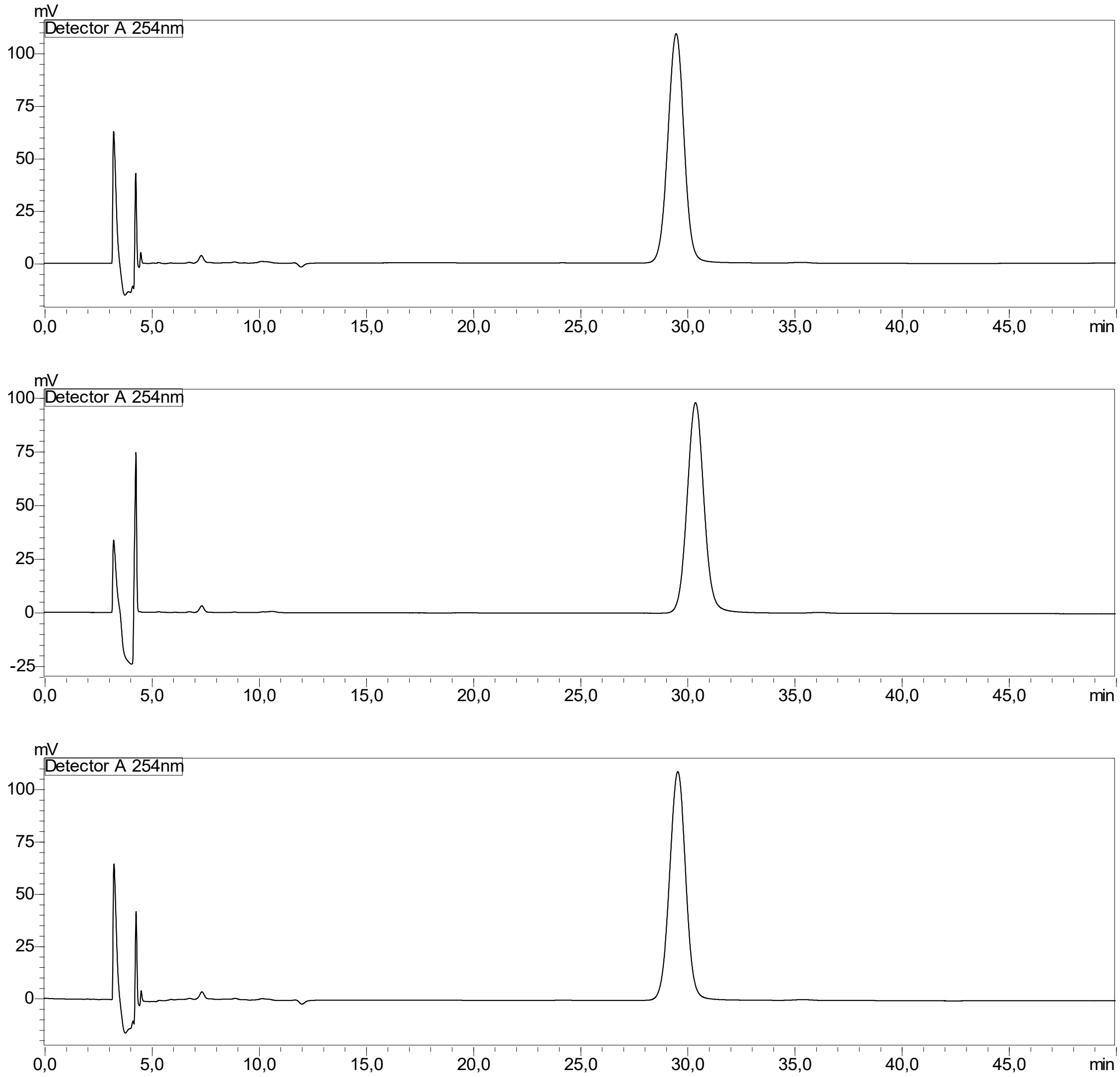

Figure S26. HPLC chromatograms of procerone A **1a**.

Table S4. Enantio-purity results of procerone A **1a**.

Compound	Run	Ret. time [min]	Peak area	Area [%]	*ee* [%]	*ee* [%] mean	St. dev.	Var. [%]
**1a**	1	29.5	5766732	99.635	99.270	**99.3**	0.07	0.07
		35.3	21135	0.365				
	2	30.4	5395772	99.646	99.291			
		36.2	19195	0.354				
	3	29.6	5755919	99.702	99.404			
		35.4	17197	0.298				

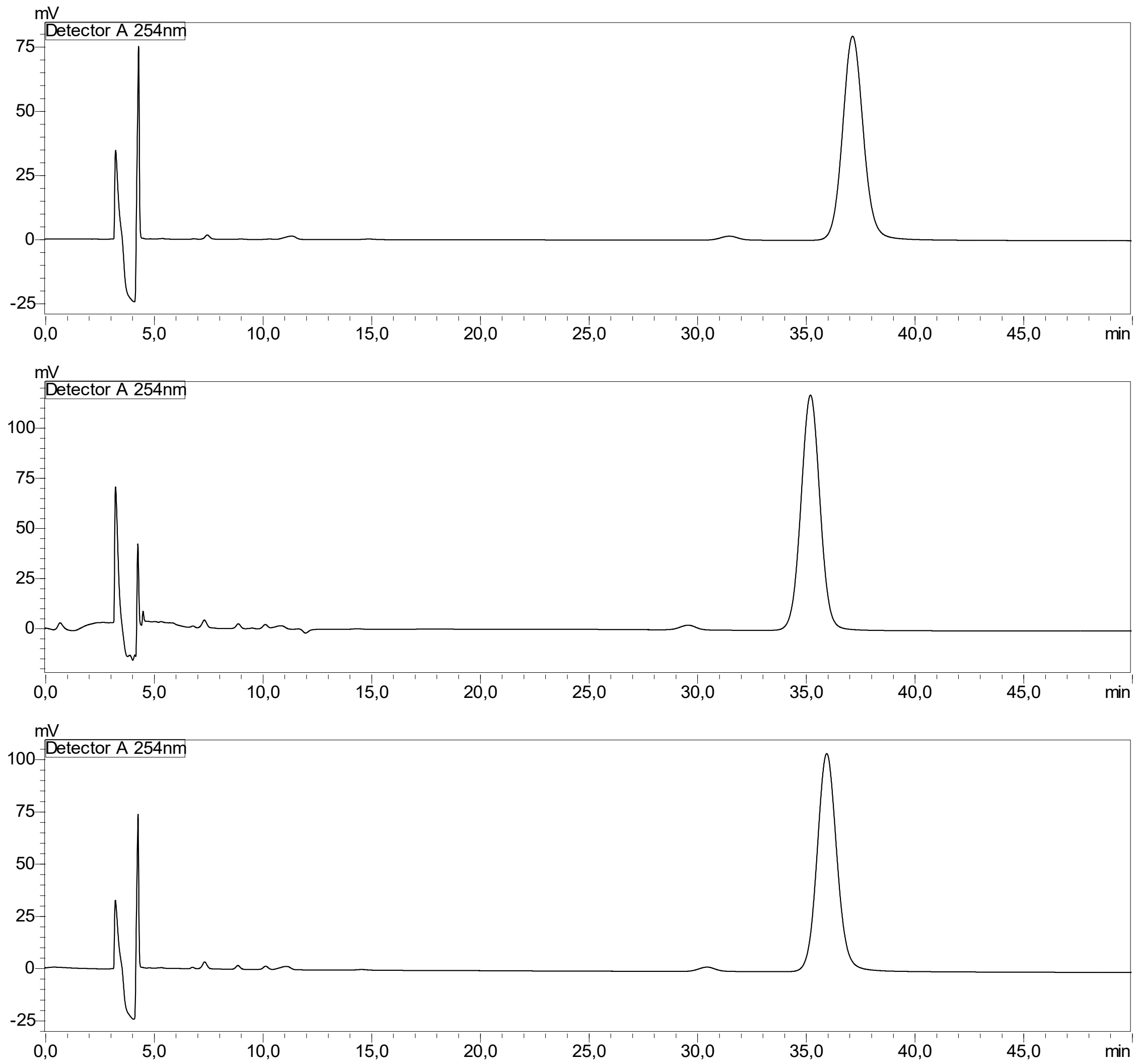

Figure S27. HPLC chromatograms of *ent*-**1a**.

Table S5. Enantio-purity results of *ent*-**1a**.

Compound	Run	Ret. time [min]	Peak area	Area [%]	*ee* [%]	*ee* [%] mean	St. dev.	Var. [%]
ent-**1a**	1	31.5	90159	1.670	96.659	**96.6**	0.03	0.03
		37.2	5307287	98.330				
	2	29.6	123454	1.697	96.605			
		35.2	7149867	98.303				
	3	30.5	113792	1.683	96.634			
		36.0	6648191	98.317				

5.2 Procerone B 1b/*ent*-1b

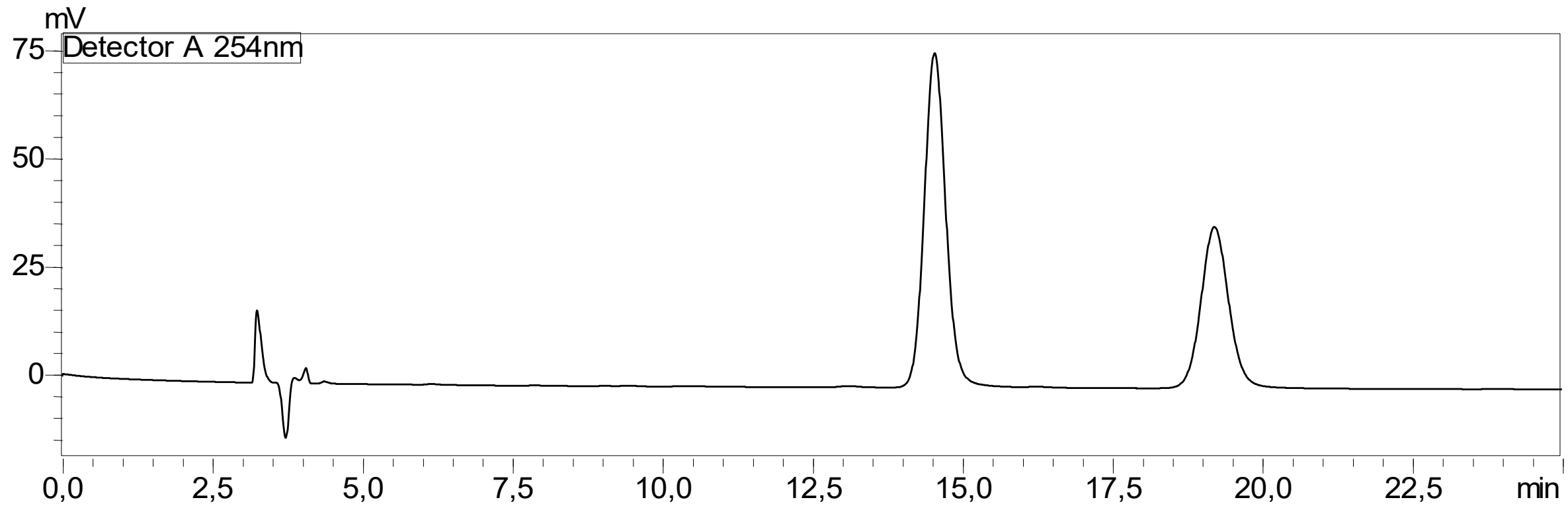

Figure S28. HPLC chromatogram of the reference of procerone B **1b**/*ent*-**1b**.

Table S6. HPLC reference data for enantio-purity measurements of procerone B **1b** and *ent*-**1b**.

Compound	Ret. time [min]	Peak area	Area [%]
Reference	14.5	1955398	61.839
	19.2	1206677	38.161

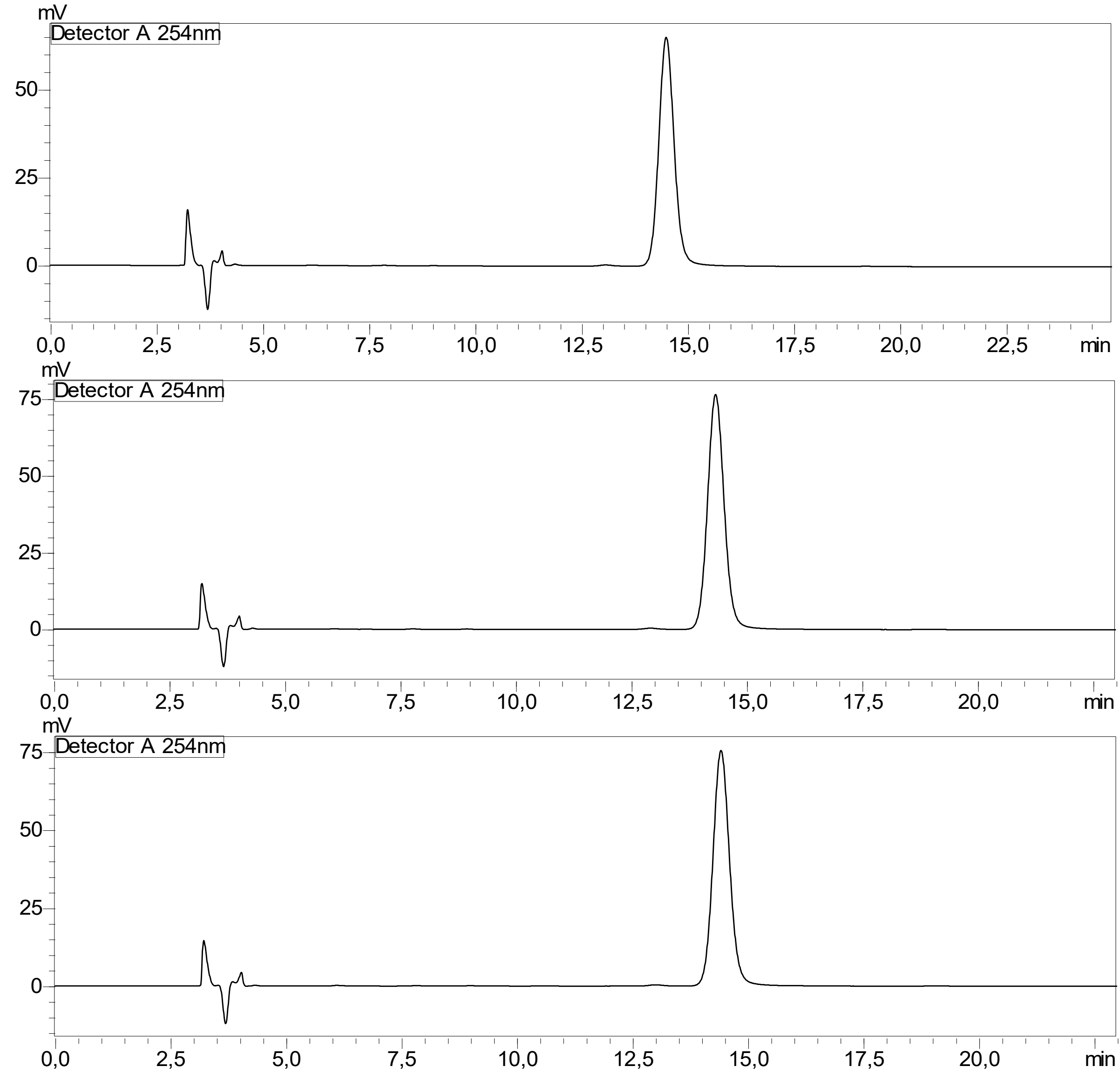

Figure S29. HPLC chromatograms of procerone B **1b**.

Table S7. Enantiopurity results of procerone B **1b**.

Compound	Run	Ret. time [min]	Peak area	Area [%]	*ee* [%]	*ee* [%] mean	St. dev.	Var. [%]
**1b**	1	14.5	1661918	99.856	99.712	**99.7**	0.01	0.01
		19.2	2397	0.144				
	2	14.4	1912819	99.863	99.726			
		19.1	2621	0.137				
	3	14.3	1933609	99.861	99.723			
		19.0	2683	0.139				

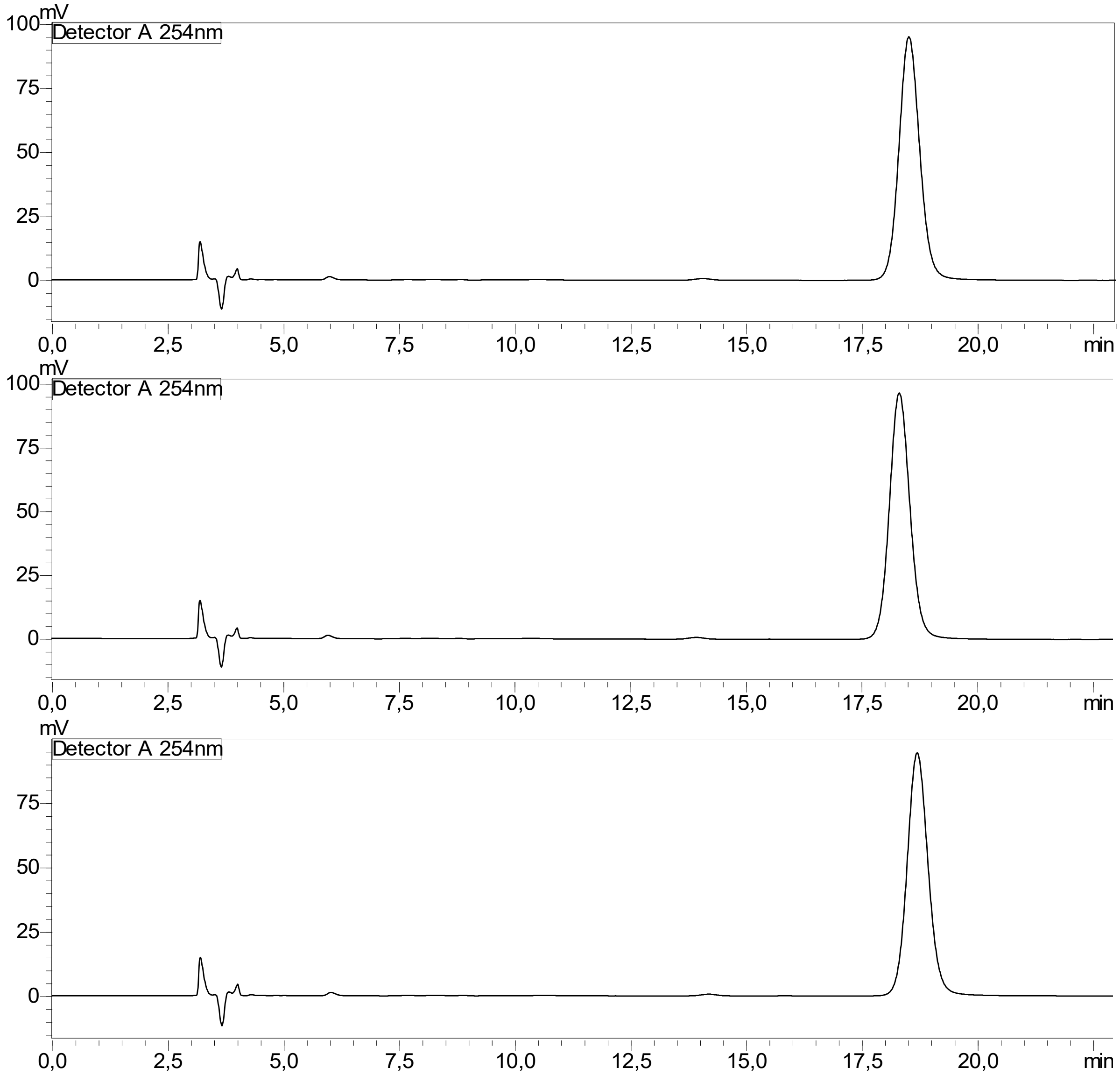

Figure S30. HPLC chromatograms of *ent*-**1b**.

Table S8. Enantiopurity results of *ent*-**1b**.

Compound	Run	Ret. time [min]	Peak area	Area [%]	*ee* [%]	*ee* [%] mean	St. dev.	Var. [%]
ent-**1b**	1	13.9	16502	0.544	98.912	**98.9**	0.02	0.02
		18.3	3017823	99.456				
	2	14.1	16371	0.541	98.918			
		18.5	3009214	99.459				
	3	14.2	15903	0.527	98.947			
		18.7	3003428	99.473				

6 Purity analysis

The purity of the tested compounds was determined via ultra-high-performance liquid chromatography (UHPLC) equipped with four detectors: ultraviolet (UV), charged aerosol detector (CAD), diode array detector (DAD) and mass spectrometry (MS). The Ultimate 3000 UHPLC system (Thermo Fisher Scientific - San Jose, CA) was equipped with a reversed-phase C18 column (Waters Acquity UPLC BEH 1.7 µm 2.1 mm × 50 mm, 130 Å). Mobile phase A (H_2O + 0.1% FA) and mobile phase B (MeCN + 0.1% FA) were degassed before their usage. A 15 min binary gradient with flow rate set to 350 µL/min was applied as follows: 0.00–10.0 min, 5% → 98% mobile phase B; 10.0–12.0 min, 98% mobile phase B (purge); 12.0–12.1 min, 5% mobile phase B; 12.1–15.0 min re-equilibration with 5% mobile phase B. The column temperature was 35 °C. Five microliters of each sample were injected, followed by a blank injection to ensure proper column washing and equilibration. Mass spectrometric detection was performed with an LTQ-XL linear ion trap mass spectrometer (Thermo Fisher Scientific) using the HESI source (300 °C heater temperature, 34.93/14.61/1.63 arb. units for the sheath, aux, and sweep gases, respectively, and 3.5 kV spray voltage at 275 °C capillary temperature) to achieve negative/positive ion mode ionization. MS scans were performed with an *m/z* range from 100 to 1500. The purity was determined from the CAD chromatogram as the relative peak area of each compound.

Table S9. LC-MS and NMR purity results of the biological tested compounds.

Compound	LC-MS purity	LC-MS purity(b)	NMR purity
Chalcone derivative **12a**	77.1%	99.3%	99.6%
Chalcone derivative **12b**	71.0%	98.3%	99.7%
Chalcone epoxide **13a**	(a)	(a)	99.1%
Chalcone epoxide **13b**	(a)	(a)	99.3%
Hydrogenated derivative **14a**	82.8%	99.9%	99.7%
Hydrogenated by-product **15a**	89.3%	98.7%	99.0%
Hydrogenated derivative **14b**	67.2%	100%	99.2%
Hydrogenated by-product **15b**	74.5%	100%	99.6%
Procerone A **1a**	96.0%	99.7%	99.3%
ent-**1a**	93.6%	98.8%	99.4%
Procerone B **1b**	97.7%	99.6%	99.5%
ent-**1b**	99.2%	99.8%	99.3%

(a) The purity check by LC-MS is not suitable under these conditions due to decomposition of the compound. (b) purity results were determined without the peaks after retention times of 10.79 min.

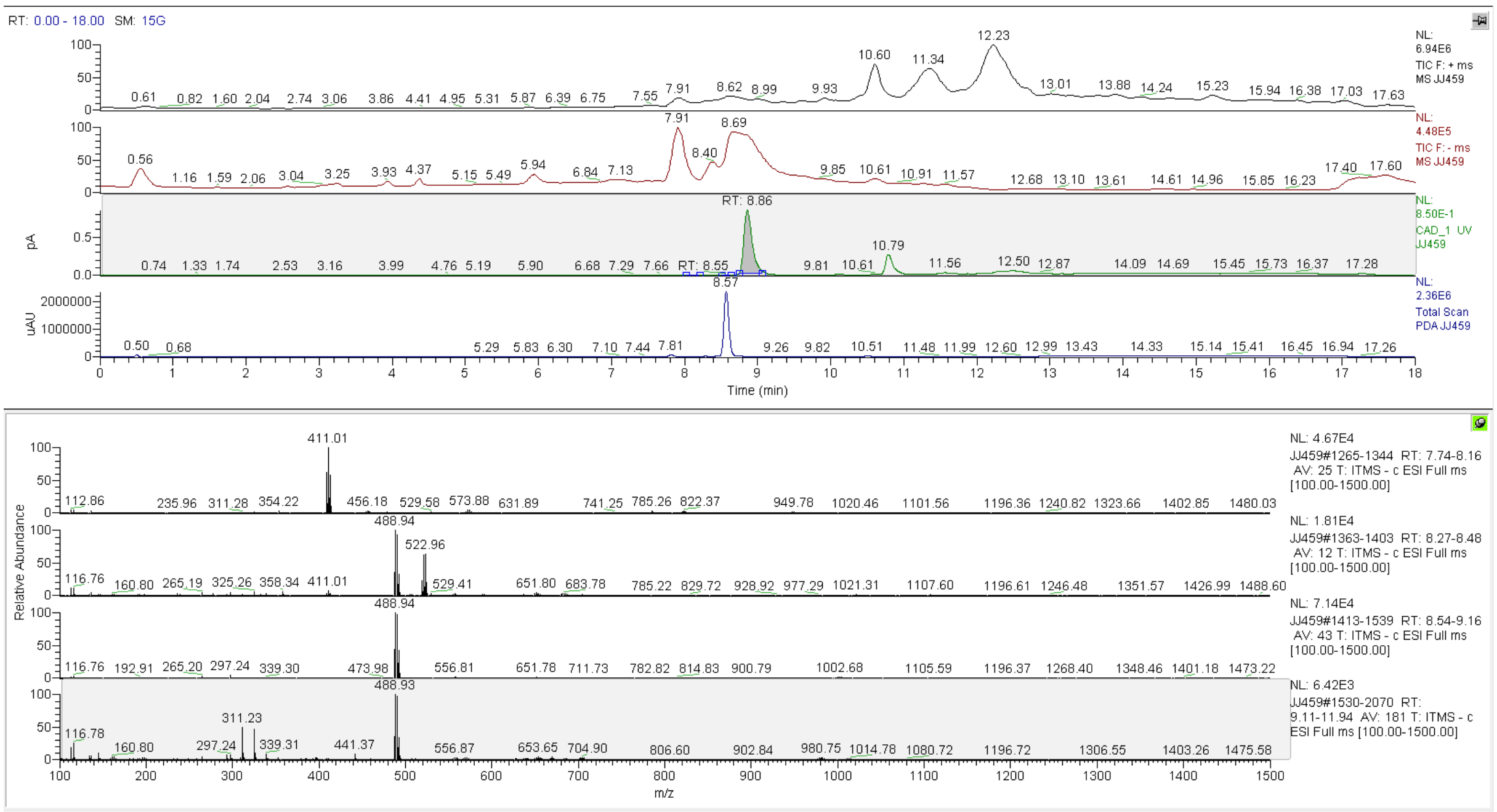

Figure S31. LC-MS spectra of chalcone derivative **12a** (from top to bottom: positive MS, negative MS, CAD, DAD, full ESI⁻ view at retention times of 7.74–8.16 min; 8.27–8.48 min; 8.54–9.16 min; 9.11–11.94 min).

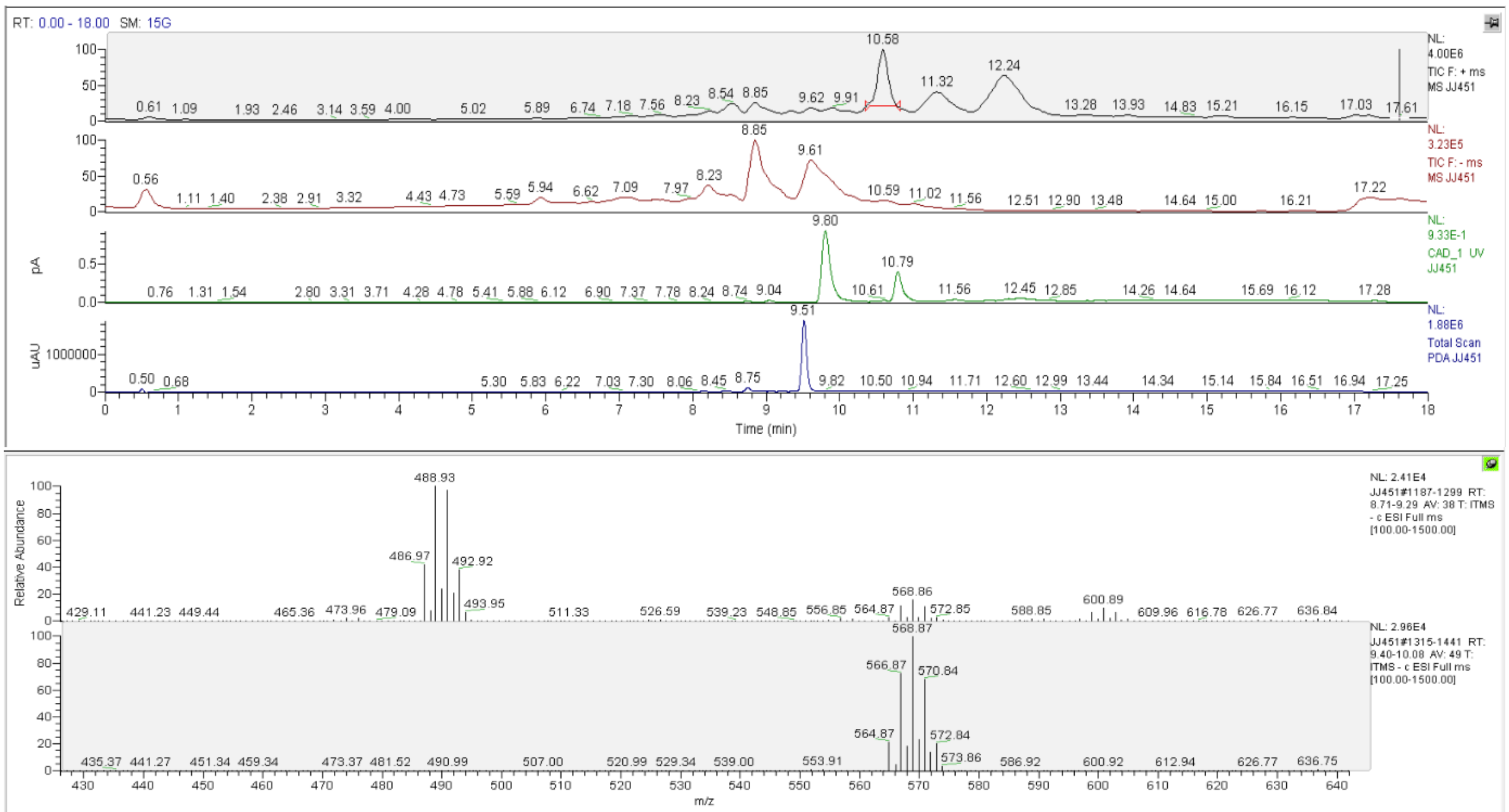

Figure S32. LC-MS spectra of chalcone derivative **12b** (from top to bottom: positive MS, negative MS, CAD, DAD, ESI⁻ view at retention times of 8.71–9.29 min; 9.40–10.08 min).

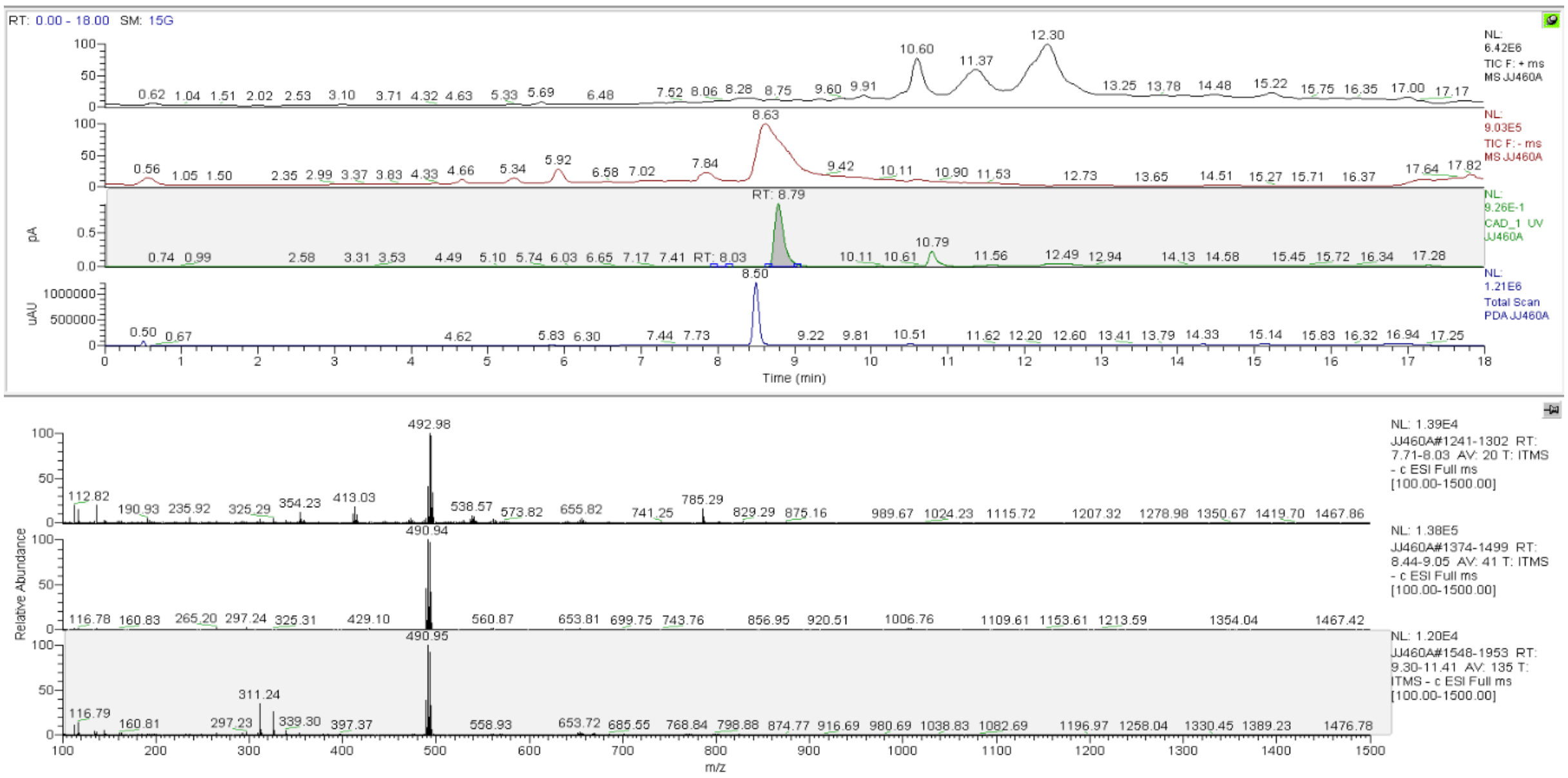

Figure S33. LC-MS spectra of hydrogenated derivative **14a** (from top to bottom: positive MS, negative MS, CAD, DAD, full ESI⁻ view at retention times of 7.71–8.03 min; 8.44–9.05 min; 9.30–11.41 min).

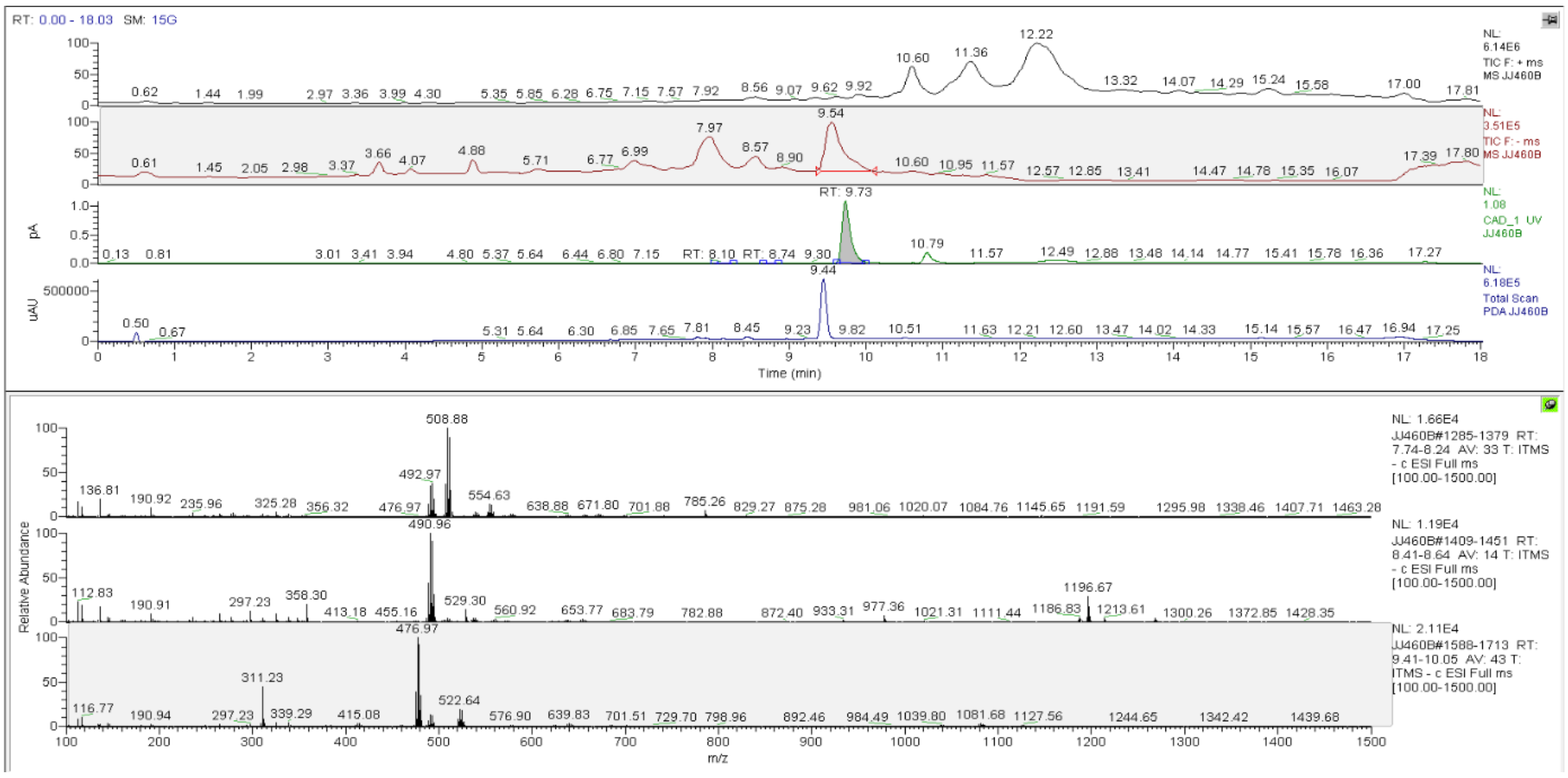

Figure S34. LC-MS spectra of hydrogenated by-product **15a** (from top to bottom: positive MS, negative MS, CAD, DAD, full ESI⁻ view at retention times of 7.74–8.24 min; 8.41–8.64 min; 9.41–10.05 min).

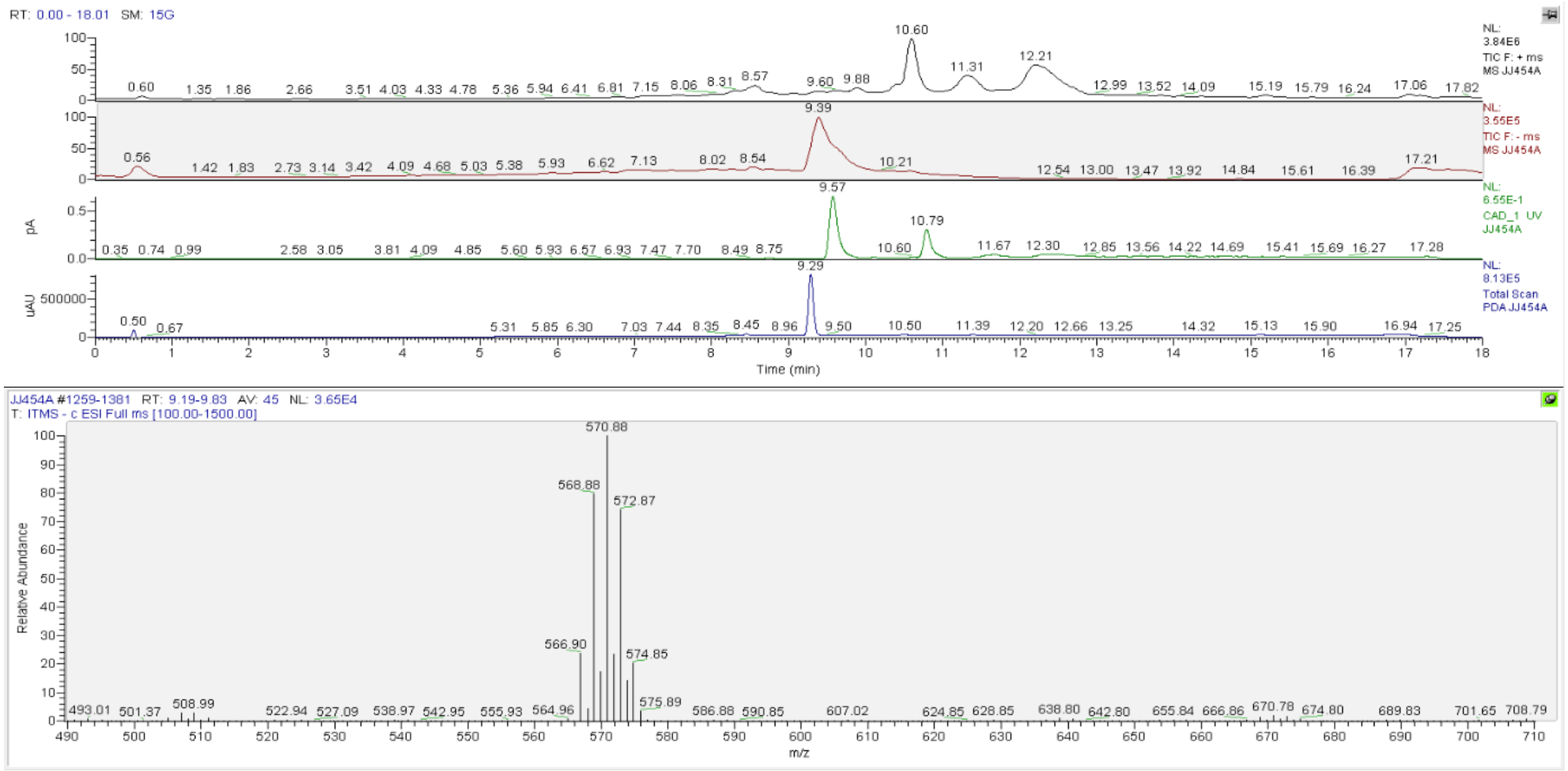

Figure S35. LC-MS spectra of hydrogenated derivative **14b** (from top to bottom: positive MS, negative MS, CAD, DAD, ESI⁻ view at retention times of 9.19–9.83 min).

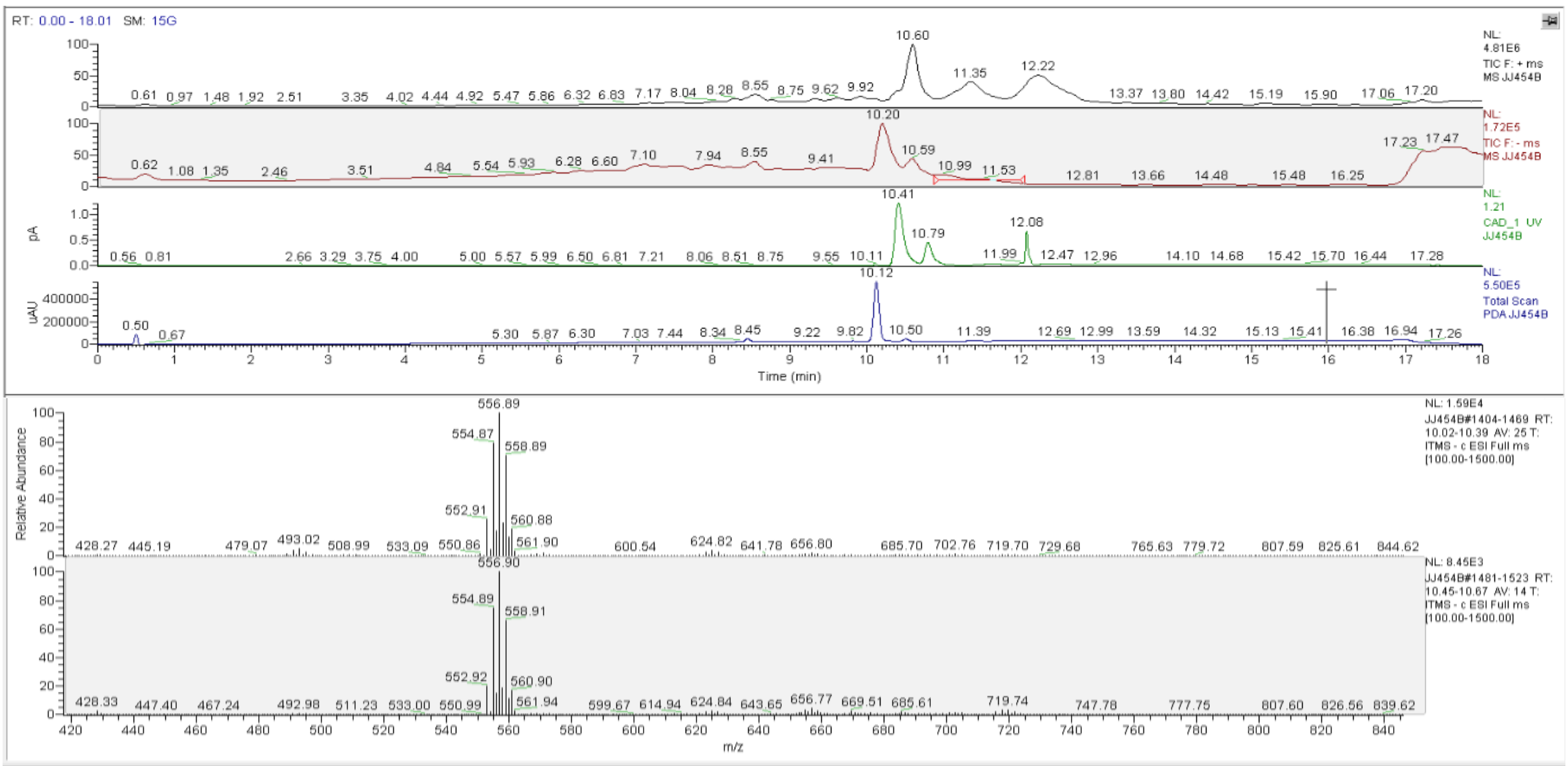

Figure S36. LC-MS spectra of hydrogenated by-product **15b** (from top to bottom: positive MS, negative MS, CAD, DAD, ESI⁻ view at retention times of 10.02–10.39 min; 10.45–10.67 min).

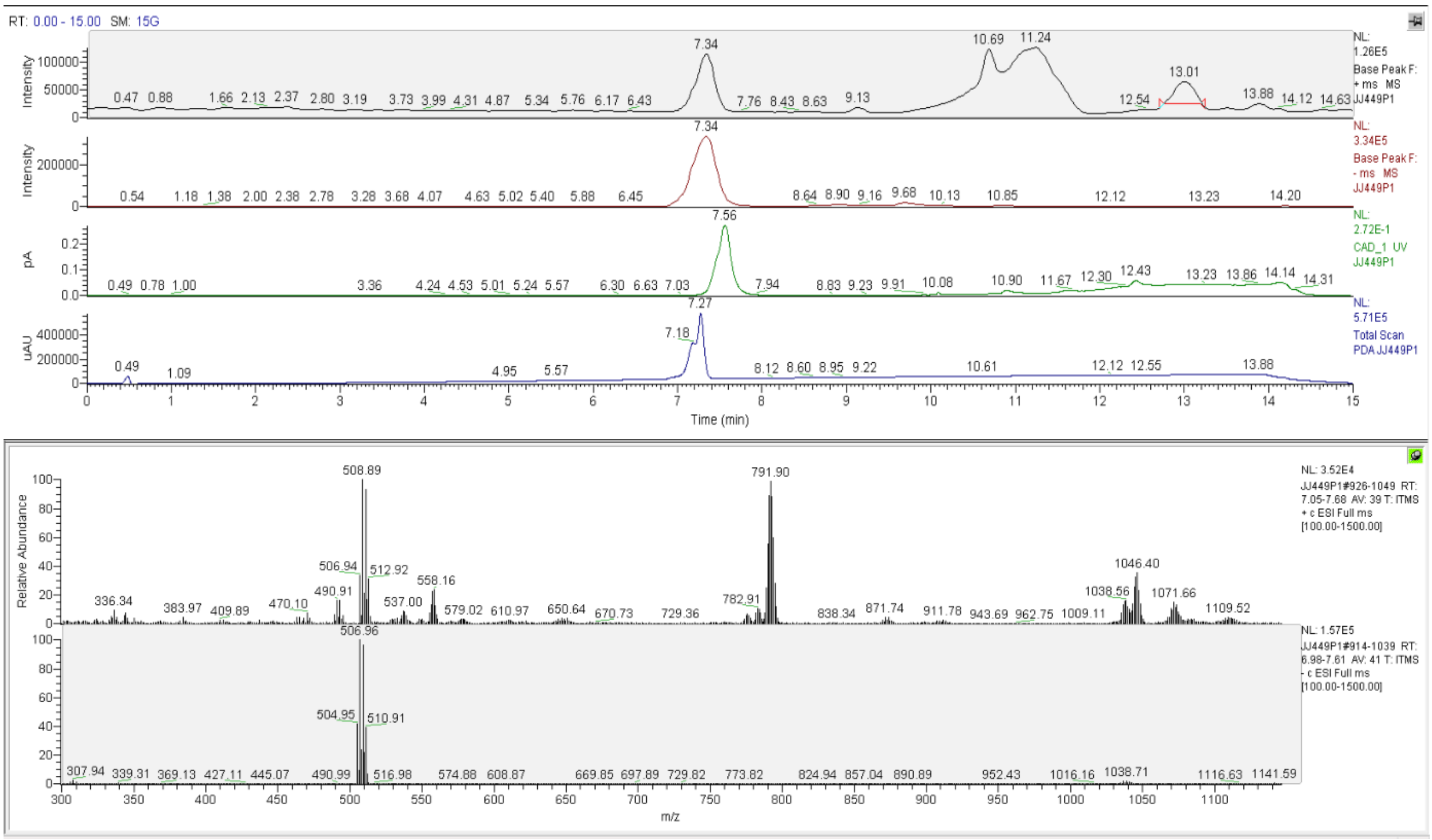

Figure S37. LC-MS spectra of procerone A **1a** (from top to bottom: positive MS, negative MS, CAD, DAD, ESI$^+$ view at retention time of 7.05–7.68 min; ESI$^-$ view at retention time of 6.98–7.61 min).

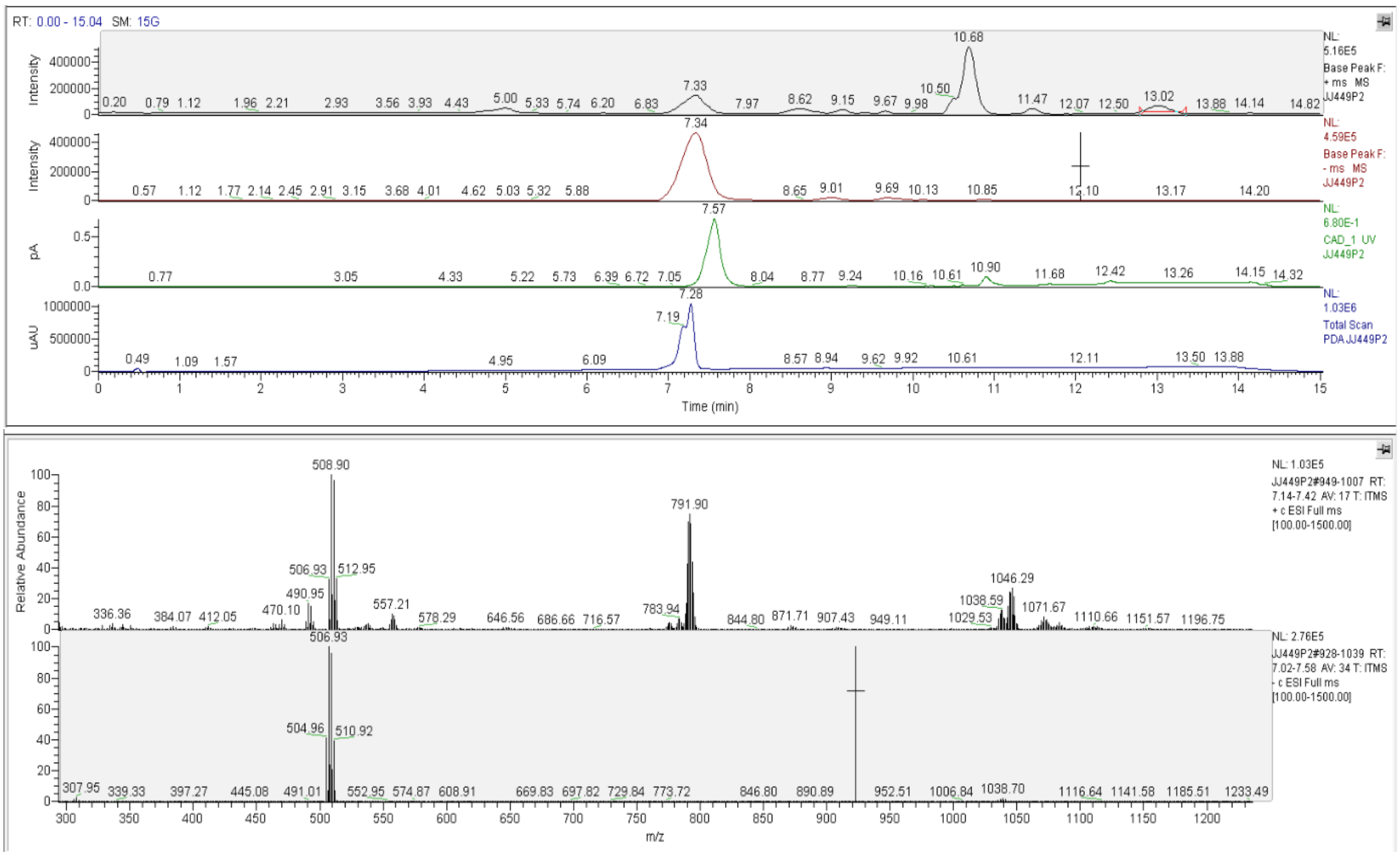

Figure S38. LC-MS spectra of *ent*-**1a** (from top to bottom: positive MS, negative MS, CAD, DAD, ESI$^+$ view at retention time of 7.14–7.42 min; ESI$^-$ view at retention time of 7.02–7.58 min).

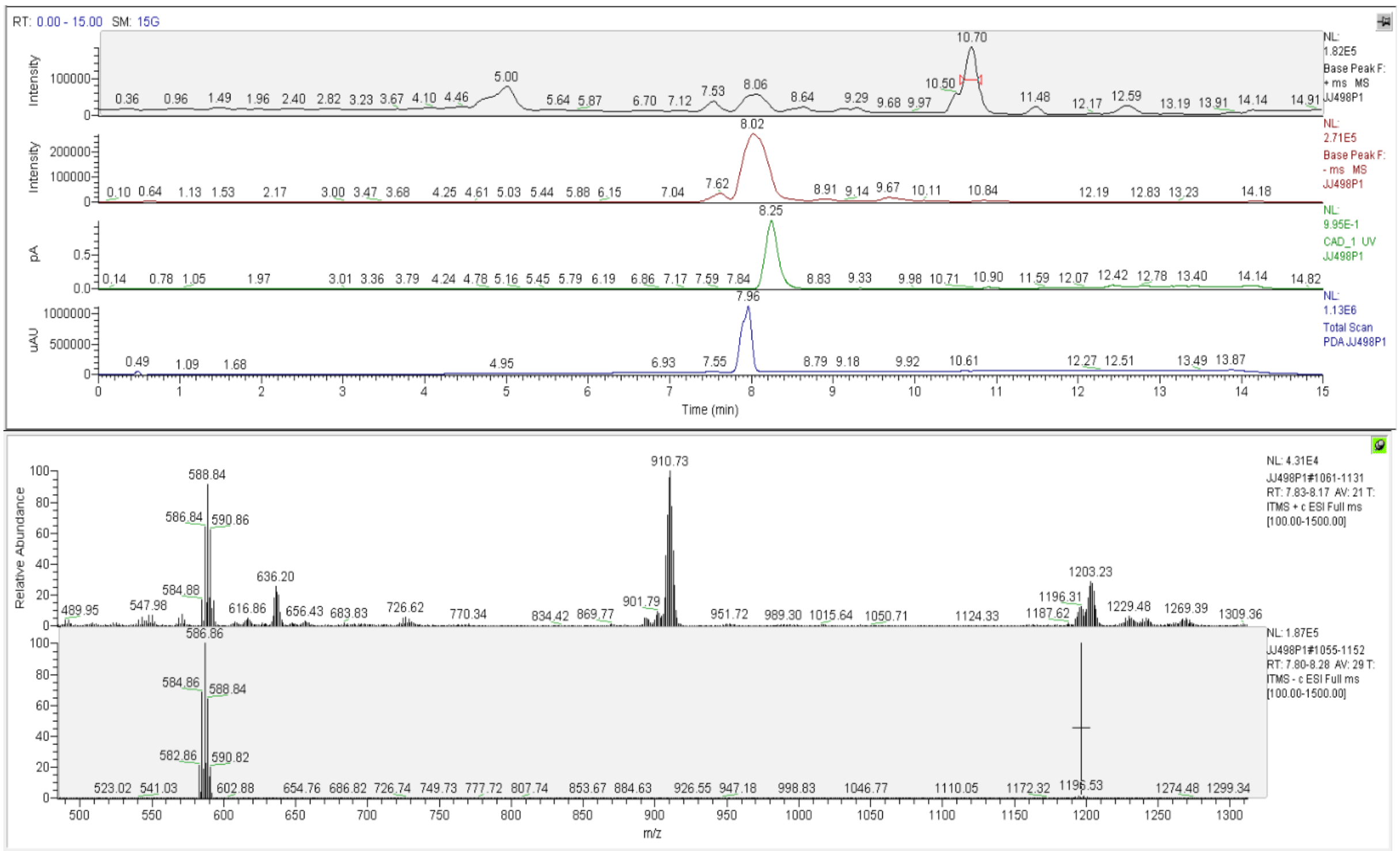

Figure S39. LC-MS spectra of procerone B **1b** (from top to bottom: positive MS, negative MS, CAD, DAD, ESI$^+$ view at retention time of 7.83–8.17 min; ESI$^-$ view at retention time of 7.80–8.28 min).

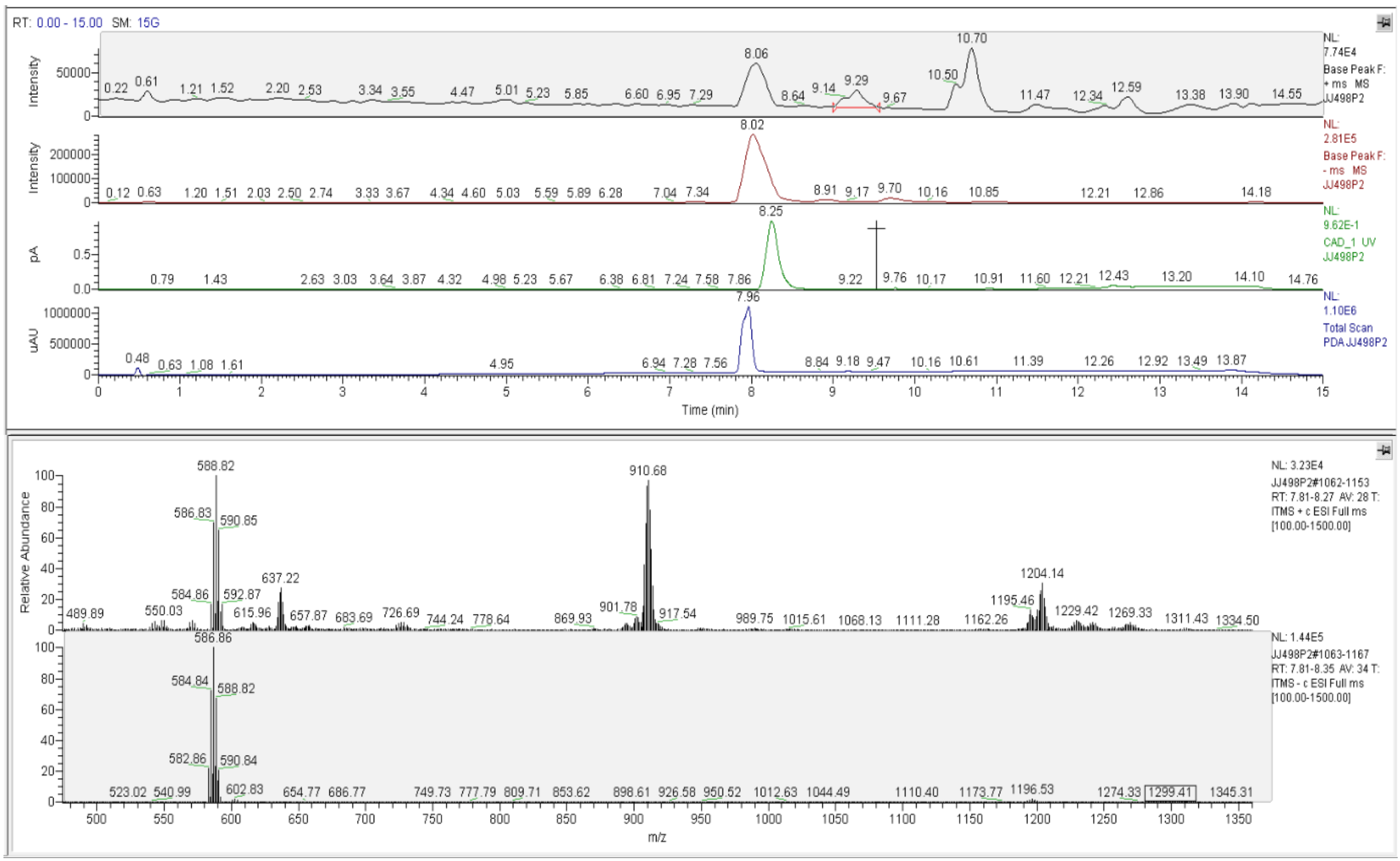

Figure S40. LC-MS spectra of *ent*-**1b** (from top to bottom: positive MS, negative MS, CAD, DAD, ESI$^+$ view at retention time of 7.81–8.27 min; ESI$^-$ view at retention time of 7.81–8.35 min).

7 Single crystal X-ray diffraction

Intensity data for procerone A **1a** were collected at 100(2) K on a Bruker D8 goniometer (Bruker AXS Inc., Madison, Wisconsin, USA) equipped with a Dectris EIGER2R 500K area detector (Dectris Ltd., Baden, Switzerland), an Incoatec IµS Diamond Cu microsource (λ = 1.54178 Å) and an Oxford Cryostream 800 instrument (Oxfordshire, UK). Data were integrated using the XDS program suite[9] and corrected for absorption by the multi-scan method with SADABS-2016/2[10]. The structures were solved by intrinsic phasing[11] and refined by full matrix least squares procedures based on F^2 as implemented in SHELXL-19[12] utilizing ShelXle[13] as a GUI. Hydrogens were treated as riding with C-H = 0.98 Å for CH3, C-H = 0.95 Å for aromatic H, C-H = 0.99 Å for methylene H and C-H = 1.00 Å for methine H. The hydroxyl hydrogens were found from the difference Fourier map and refined as riding with a distance restraint of O-H = 0.9 Å. The hydrogens were assigned isotropic displacement parameters constrained to Uiso(H) = 1.5 Ueq(C,O) for methyl groups and hydroxy hydrogens or Uiso(H) = 1.2 Ueq(C) of their parent atoms otherwise. Structure was validated with CHECKCIF (https://checkcif.iucr.org/). See the respective CIF file for exact versions and more details.

7.1 Procerone A 1a

These data are deposited at the Cambridge Crystallographic Data Centre and can be obtained via www.ccdc.cam.ac.uk/data_request/cif (CCDC 2471100).

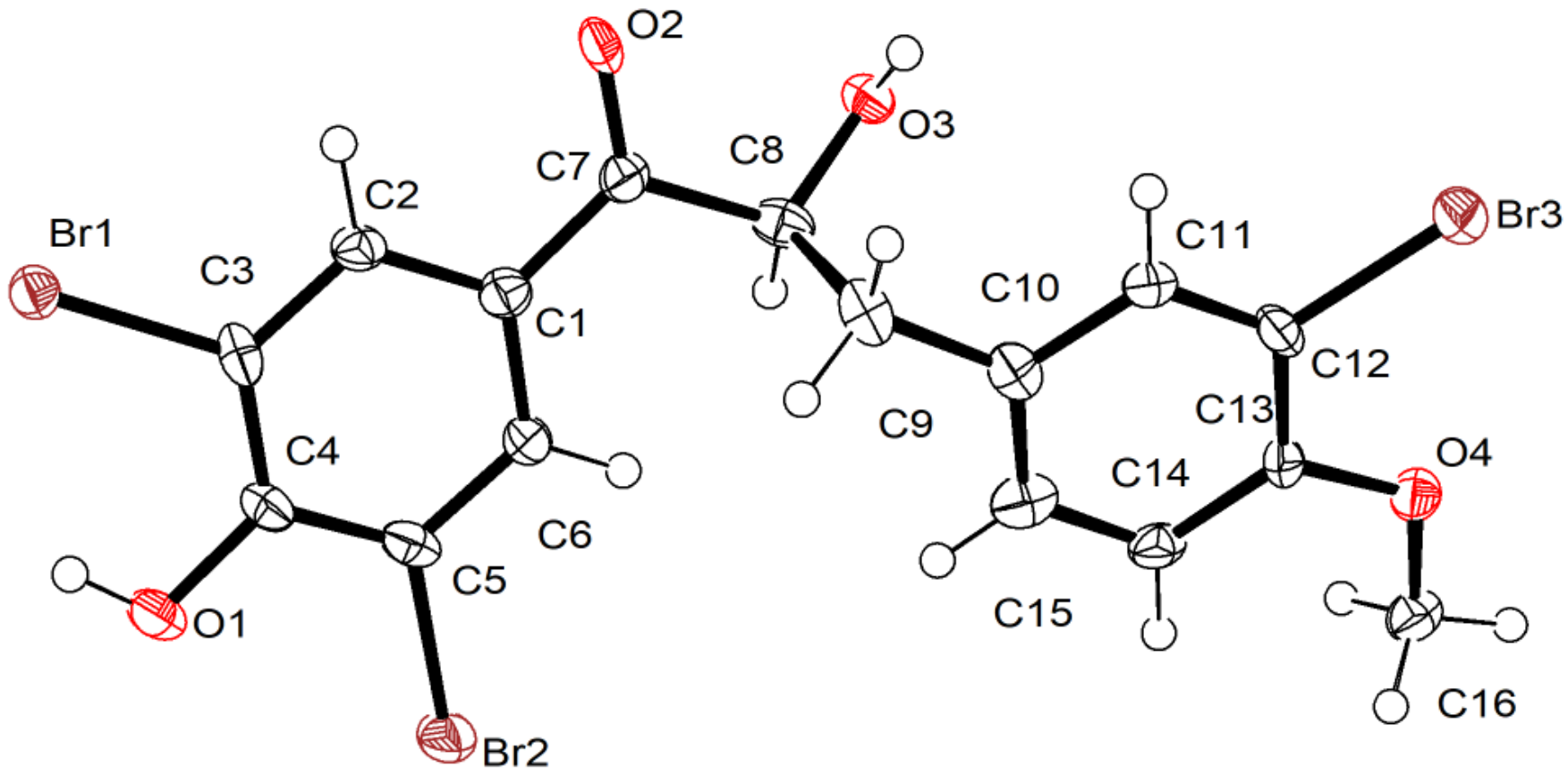

Figure S41. Displacement ellipsoid plot[14] (50% probability) of the asymmetric residue in the crystal structure of procerone A **1a** with atom labels.

Table S10. SXRD data for procerone A **1a**.

**Datablock: procerone_a**			
Bond precision:	C-C = 0.0153 A		Wavelength = 1.54178
Cell:	a = 29.077 (6)<br>alpha = 90	b = 29.982 (6)<br>beta = 90	c = 7.5765 (15)<br>gamma = 90
Temperature:	100 K		
	Calculated		Reported
Volume	6605 (2)		6605 (2)
Space group	F d d 2		F d d 2
Hall group	F 2 -2d		F 2 -2d
Moiety formula	C16 H13 Br3 O4		C16 H13 Br3 O4
Sum formula	C16 H13 Br3 O4		C16 H13 Br3 O4
Mr	508.96		508.99
Dx,g cm-3	2.047		2.047
Z	16		16
Mu (mm-1)	9.220		9.220
F000	3936.0		3936.0
F000’	3911.30		
h, k, lmax	36, 37, 9		36, 37, 9
Nref	3497[1885]		3357
Tmin, Tmax	0.288, 0.832		0.524, 0.755
Tmin’	0.218		

Correction method = # Reported T Limits: Tmin = 0.524 Tmax = 0.755
AbsCorr = MULTI-SCAN

Data completeness = 1.78/0.96		Theta (max) = 77.058
R (reflections) = 0.0506 (3332)		wR2 (reflections) =0.1288 (3357)
S = 1.038	Npar = 216	

8 Electronic circular dichroism

8.1 Experimental

ECD measurements were performed with Applied Photophysics Chirascan Plus using quartz cuvettes with 1 mm pathlength. Samples were dissolved in methanol (c = 0.5 mg/mL) and measured with 5 repetitions each before applying the average.

Table S11. Experimental results of the ECD measurements of procerones A **1a**/*ent*-**1a** and B **1b**/*ent*-**1b**.

nm	**1a**	*ent*-**1a**	**1b**	*ent*-**1b**
400	−0.180029	−0.273988	−0.244318	−0.204354
399	−0.142251	−0.274709	−0.212323	−0.22233
398	−0.145602	−0.289376	−0.231452	−0.226339
397	−0.165275	−0.314363	−0.250719	−0.2113
396	−0.210529	−0.328251	−0.265802	−0.270716
395	−0.180679	−0.343129	−0.248344	−0.217923
394	−0.184454	−0.278164	−0.266513	−0.229968
393	−0.117659	−0.276607	−0.223125	−0.16871
392	−0.140074	−0.278606	−0.215056	−0.199907
391	−0.168173	−0.312549	−0.266832	−0.176482
390	−0.0927361	−0.220565	−0.179544	−0.114498
389	−0.159101	−0.292719	−0.233062	−0.140026
388	−0.125768	−0.238793	−0.169814	−0.140011
387	−0.112956	−0.226608	−0.122943	−0.129561
386	−0.232958	−0.341152	−0.324575	−0.242631
385	−0.100953	−0.176398	−0.15479	−0.100144
384	−0.153555	−0.186666	−0.15456	−0.0910534
383	−0.0966433	−0.175902	−0.150827	−0.117468
382	−0.165264	−0.223038	−0.196108	−0.138857
381	−0.111007	−0.169739	−0.166051	−0.100097
380	−0.236444	−0.287633	−0.254798	−0.198585
379	−0.0759367	−0.151105	−0.101075	−0.0681429
378	−0.180678	−0.187932	−0.200327	−0.124013
377	−0.131775	−0.166775	−0.191713	−0.089261
376	−0.195738	−0.164865	−0.180696	−0.111308
375	−0.166945	−0.140598	−0.216789	−0.0983152
374	−0.20293	−0.173665	−0.219397	−0.0987183
373	−0.2494	−0.154618	−0.215004	−0.0815816
372	−0.134651	−0.0445011	−0.146269	−0.0144561
371	−0.179907	−0.155917	−0.233052	−0.0575861
370	−0.190122	−0.165501	−0.206442	−0.0849416
369	−0.138995	−0.098755	−0.137186	−0.0357834
368	−0.123456	−0.165696	−0.171392	−0.0592704
367	−0.214561	−0.24032	−0.272857	−0.143712
366	−0.151689	−0.250183	−0.250928	−0.127243
365	−0.0310866	−0.152692	−0.107376	−0.0513048

364	−0.0851549	−0.28269	−0.204313	−0.188472
363	−0.0612268	−0.318897	−0.172668	−0.166047
362	0.144565	−0.227956	−0.039288	−0.0514532
361	0.0493609	−0.343547	−0.118233	−0.16922
360	0.0919106	−0.368108	−0.1076	−0.185081
359	0.15595	−0.326821	−0.0999107	−0.179913
358	0.161821	−0.347461	−0.0885515	−0.144176
357	0.179634	−0.328257	−0.101286	−0.172342
356	0.111303	−0.392552	−0.12784	−0.156233
355	0.109515	−0.364655	−0.150971	−0.191991
354	0.0447648	−0.425372	−0.231345	−0.224493
353	0.111713	−0.38283	−0.146435	−0.133081
352	0.117508	−0.412831	−0.219986	−0.166172
351	0.179861	−0.382307	−0.181841	−0.118038
350	0.256118	−0.305008	−0.120325	−0.0857161
349	0.32038	−0.299385	−0.0758912	−0.00173169
348	0.172715	−0.483435	−0.241773	−0.150662
347	0.301982	−0.398725	−0.195416	−0.108002
346	0.343543	−0.399644	−0.204712	−0.0934457
345	0.381651	−0.302902	−0.170103	−0.107259
344	0.368314	−0.322329	−0.213464	−0.0661837
343	0.349608	−0.321088	−0.242371	−0.0548063
342	0.306221	−0.315962	−0.316585	−0.0434743
341	0.324093	−0.230921	−0.286327	0.0282219
340	0.315156	−0.192247	−0.315304	0.0713744
339	0.240158	−0.136906	−0.369231	0.134675
338	0.271584	−0.080905	−0.349538	0.228371
337	0.18541	−0.0598978	−0.432081	0.260877
336	0.126858	−0.141501	−0.54645	0.224983
335	0.139088	−0.125807	−0.590872	0.344313
334	0.122693	−0.0796595	−0.603137	0.413757
333	0.0647423	−0.139231	−0.701138	0.409185
332	0.0650301	−0.186155	−0.707483	0.438809
331	−0.00147499	−0.20864	−0.795956	0.519923
330	0.0944662	−0.164588	−0.786104	0.637971
329	−0.114231	−0.242122	−0.959028	0.545358
328	−0.0753553	−0.204187	−0.956789	0.692475
327	−0.16711	−0.13483	−1.08	0.70273
326	−0.129105	−0.0852463	−1.02666	0.906366
325	−0.353555	−0.136465	−1.20077	0.869579
324	−0.262683	0.0172109	−1.15444	1.02937
323	−0.394813	−0.0785143	−1.31825	1.02111
322	−0.400528	0.0307957	−1.30438	1.13036
321	−0.516611	−0.0516095	−1.40132	1.11932
320	−0.500592	−0.0142437	−1.42983	1.22364
319	−0.66845	−0.116596	−1.5335	1.11364

318	−0.547403	−0.119382	−1.51203	1.20225
317	−0.463057	−0.100716	−1.43314	1.23091
316	−0.447498	−0.0979118	−1.43158	1.23088
315	−0.422934	−0.283032	−1.49139	1.13046
314	−0.347272	−0.369978	−1.43471	1.07359
313	−0.209852	−0.498613	−1.35737	1.07438
312	0.0166616	−0.695971	−1.25792	0.984006
311	0.171437	−0.946714	−1.20081	0.841093
310	0.534428	−1.18781	−0.996962	0.748937
309	0.910709	−1.61939	−0.800813	0.562467
308	1.35693	−2.13917	−0.626769	0.227457
307	2.1074	−2.62514	−0.23673	−0.00198918
306	2.86903	−3.27502	0.192941	−0.36561
305	3.79506	−4.05944	0.667423	−0.880895
304	4.88035	−5.1009	1.20891	−1.44125
303	6.13107	−6.12139	1.87058	−2.06725
302	7.37032	−7.1491	2.58639	−2.73365
301	8.67843	−8.28946	3.33325	−3.46739
300	9.89543	−9.415	4.00759	−4.25035
299	11.1213	−10.4433	4.73224	−4.98491
298	12.3357	−11.3557	5.5504	−5.70615
297	13.1583	−12.2488	6.19651	−6.58447
296	14.0255	−12.8791	6.99674	−7.22882
295	14.5762	−13.4603	7.74233	−8.00665
294	14.8783	−13.7749	8.42424	−8.69997
293	14.7356	−13.5673	9.17344	−9.43497
292	14.2651	−13.1239	9.68768	−9.99767
291	13.2967	−12.331	10.1937	−10.3292
290	12.0936	−11.3879	10.3101	−10.5543
289	11.2274	−10.4356	10.3006	−10.5361
288	10.1233	−9.48635	10.0421	−10.2863
287	9.33817	−8.82366	9.69135	−9.89813
286	8.59731	−8.20837	9.05502	−9.35269
285	7.68233	−7.29914	8.65944	−8.8736
284	6.87364	−6.48859	8.2815	−8.24584
283	5.92642	−5.61171	7.75439	−7.85172
282	5.11613	−4.77142	7.24468	−7.22392
281	4.52205	−4.00274	6.72892	−6.69492
280	3.87439	−3.67886	6.00336	−6.1287
279	3.604	−3.39052	5.36373	−5.44948
278	3.4465	−3.08482	4.91415	−4.79425
277	3.25762	−2.97169	4.43517	−4.47215
276	3.22398	−3.0009	4.10674	−4.10335
275	3.23166	−2.8842	3.83262	−3.85784
274	3.32123	−2.86116	3.79037	−3.67456
273	3.32772	−2.95608	3.48661	−3.64085

272	3.32016	−3.00751	3.37159	−3.50393
271	3.54951	−3.12202	3.21533	−3.25433
270	3.61141	−2.97403	3.25104	−3.10387
269	3.79129	−3.15964	3.06644	−3.12127
268	3.72234	−3.19009	2.96646	−3.10036
267	3.6335	−3.12215	2.85747	−3.10908
266	3.67349	−2.92674	2.91777	−2.91713
265	3.46422	−2.98939	2.72823	−3.01161
264	3.45546	−2.80777	2.6371	−2.91603
263	2.93236	−2.56075	2.53273	−2.91236
262	2.80655	−2.34807	2.52063	−2.87526
261	2.72807	−2.01414	2.47879	−2.73202
260	2.20665	−1.9739	2.31999	−2.82563
259	2.07739	−1.61513	2.34719	−2.78584
258	1.90504	−1.45452	2.4107	−2.74207
257	1.61066	−1.35098	2.17307	−2.82195
256	1.55986	−1.2213	2.24463	−2.77938
255	1.3862	−0.981564	2.14164	−2.72326
254	1.20476	−0.987337	2.06515	−2.75794
253	1.11523	−0.967274	1.93183	−2.78881
252	1.14886	−0.971282	1.87045	−2.60879
251	1.17281	−0.885935	1.72873	−2.4096
250	1.00637	−0.718174	1.48404	−2.11226
249	0.767414	−0.690122	1.03542	−1.94057
248	0.389816	−0.236466	0.682595	−1.47133
247	0.0110652	0.109864	0.245383	−0.946672
246	−0.533885	0.699424	−0.379704	−0.390943
245	−1.4586	1.54988	−0.929694	0.256673
244	−2.40558	2.4225	−1.51777	0.927161
243	−3.44788	3.61545	−2.32631	1.52735
242	−4.32047	4.68002	−2.86445	2.40022
241	−5.26544	5.49084	−3.61574	3.03044
240	−5.54343	6.51032	−4.14438	3.63585
239	−5.65503	7.08249	−4.64326	4.27985
238	−5.54353	7.53041	−5.22781	4.94243
237	−5.185	7.45583	−5.77846	5.29614
236	−4.3794	7.3633	−5.98033	5.82
235	−3.57287	6.83669	−6.48857	6.13732
234	−2.6689	6.11679	−6.69246	6.47428
233	−1.59336	5.53972	−6.87981	6.69127
232	−0.845709	4.66081	−6.91604	6.97409
231	−0.147946	4.32579	−6.83424	7.07906
230	0.303585	3.91235	−6.51538	7.02678
229	0.57051	3.45599	−6.14152	6.82071
228	1.00872	3.33721	−5.67785	6.81563
227	1.12069	3.07461	−5.28645	6.38369

226	1.27336	2.87491	−4.62637	5.98827
225	1.4177	2.8544	−4.01048	5.67512
224	1.5197	2.64003	−3.34177	5.02255
223	1.53729	2.54374	−2.45254	4.39537
222	1.64987	2.50561	−1.88414	4.08558
221	1.69601	2.53436	−1.28356	3.58433
220	1.60595	2.29673	−1.01621	3.16258
219	1.57008	2.25754	−0.539757	2.74964
218	1.82957	2.4921	−0.122511	2.89527
217	1.81573	2.44043	0.152713	2.75519
216	1.80859	2.33177	−0.0598155	2.45974
215	1.93357	2.39717	0.300687	2.76883
214	2.09161	2.40321	0.503727	2.93198
213	2.24809	2.46877	0.747979	3.03964
212	2.30435	2.47598	0.929178	2.99366
211	2.28815	2.56565	1.49692	3.18294
210	2.50823	2.62384	1.90956	3.09534
209	2.48458	2.61004	2.15527	3.09104
208	2.6457	2.73063	2.44954	3.00082
207	2.66193	2.75374	2.44106	3.12086
206	2.51698	2.73006	2.41685	2.93067
205	2.69414	2.75838	2.34211	2.87003
204	2.6682	2.8513	2.49215	2.85273
203	2.59087	2.81133	2.43542	2.6965
202	2.50533	2.64782	2.46031	2.45625
201	2.51959	2.59039	2.53007	2.08027
200	2.29518	2.43405	2.70941	1.41976

8.2 Calculated

Absolute configuration assignment of procerones A **1a** and B **1b**, together with their synthetic enantiomers (*ent*-**1a**/*ent*-**1b**), was carried out following a slightly modified version of a protocol described elsewhere.[15,16] The calculation scheme is shown in Figure S42 and is consistent on a vast conformational search, for covering the whole conformational space. It produced 65 conformers for **1a**, while 50 conformers were found for **1b**. Geometries were optimized at DFT (mpw1pw91/6-31+g(d,p)). After DFT minimization several geometries collapsed into the same conformers; structural redundancy was removed by applying a 0.5 Å RMSD filter above heavy atoms. The conformer population of **1a** (*eight conformers*) and **1b** (*four conformers*) was assigned based on single point energy calculations, including thermal contributions, at a higher theory level (PBE0/Def2-TZVP). Relevant conformers and their populations are shown in Table S12. The structures of the whole conformational space of both compounds are shown in Figure S43 and Figure S44. ECD of procerones were computed at DFT level and compared against its experimental counterparts, resulting curves matches each other very well and are shown in Figure 3 (main text) as well as in additional figures (Figure S45 and Figure S46).

Computational details

Conformational search was carried out in PCModel V 10.075[17]; using: Min. Cut off Distance 0.5 Å; Min. Cut off Energy 0.15 kcal mol^{-1}; Stop Criteria: E. min. found 5000 times; Duplicate Conf. found 2000 times; Min. Conf. Minimized is 20000; Max. Conf. Minimized is 100000. Energy Window 100 kcal mol^{-1}; Module GMMX[18]; Search criteria: Random (applied to both bonds and cartesian). All rings and bonds were included in the search. Found geometries were treated with the force field MMFF94[19,20]. DFT calculations were carried out in Gaussian V16.[21] Geometry Optimization was performed on all the conformers found at mpw1pw91[22]/6-31+g(d,p) and solvent (MeOH) was modelled by iefpcm[23]. The true minima in the optimized geometries were assured by frequency calculation at the same level. A Single Point Energy (SPE) calculation, including thermal corrections taken from the frequency calculations, was carried out on all the conformers at PBE0[24]/Def2-TZVP[25,26]/W06, and solvent (MeOH) modelled with COSMO[27], to accurately estimate the conformer populations. Structural redundancy among DFT optimized geometries was handled using an in-house Python script. The script builds a heavy-atom molecular graph (edges represent heavy-atom bonds and are labelled by bond order)[28], enumerates symmetry-equivalent atom index permutations via graph automorphisms (matching atom types and bond orders within a tolerance), and then superimposes conformers using the Kabsch algorithm[29,30] (optimal rigid-body rotation without reflection), before computing heavy-atom RMSDs. Enantiomer structures of **1a** (*ent*-**1a**) and **1b** (*ent*-**1b**) were generated by mirror reflection through the yz-plane ($x \rightarrow -x$) over DFT optimized conformers.[31] ECD was computed using the following functional/basis set combinations PW6B95D3[32]/Def2TZVPP[33]/W06; PBE0/Def2TZVPP/W06; CAM-B3LYP[34]/x2c-TZVPall-2c[35]/auto and WB97XD[36]/x2c-TZVPall-2c. Those functionals not available in Gaussian 16 were downloaded from the Basis Set Exchange web site.[37,38] Experimental and simulated ECD data were handled with SpecDis V 1.70.[39]

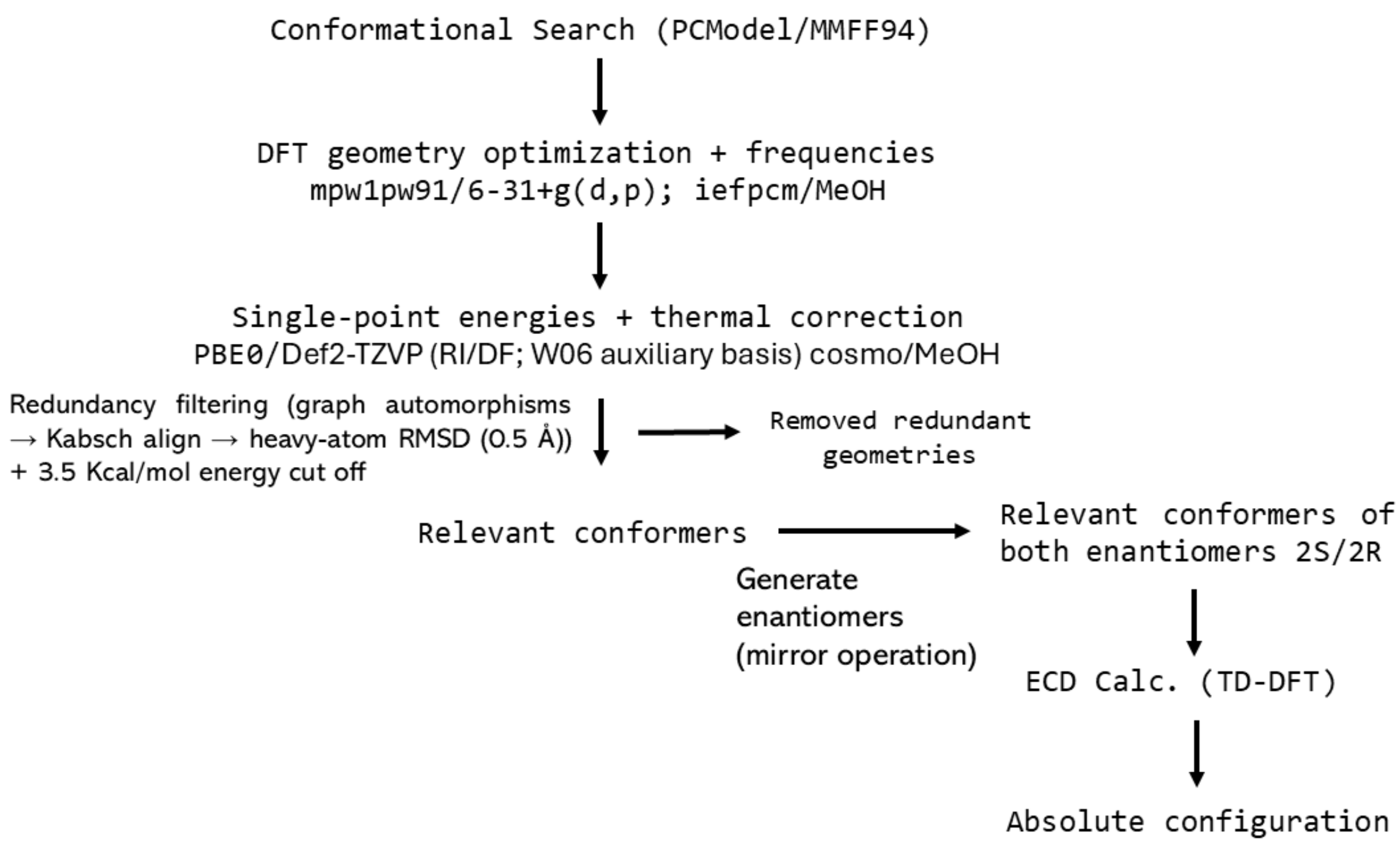

Figure S42. General scheme used in the ECD calculation of procerones.

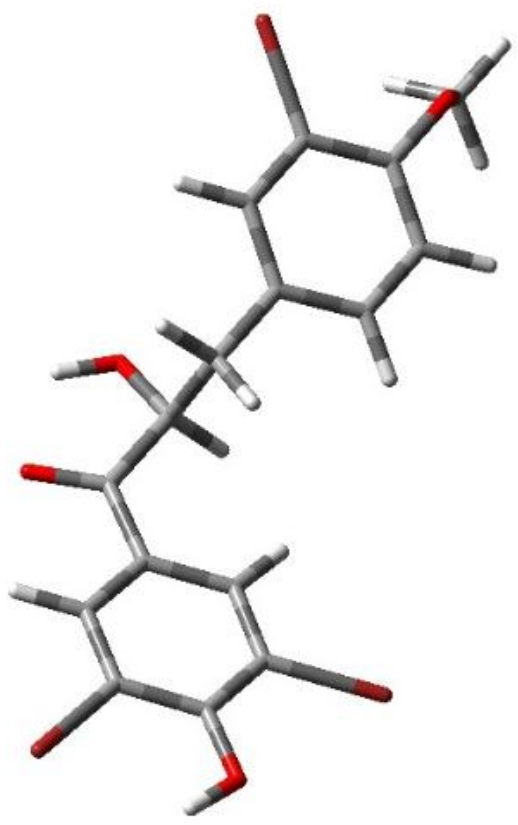

2*S*-1a-Conf-7 (55.67%)

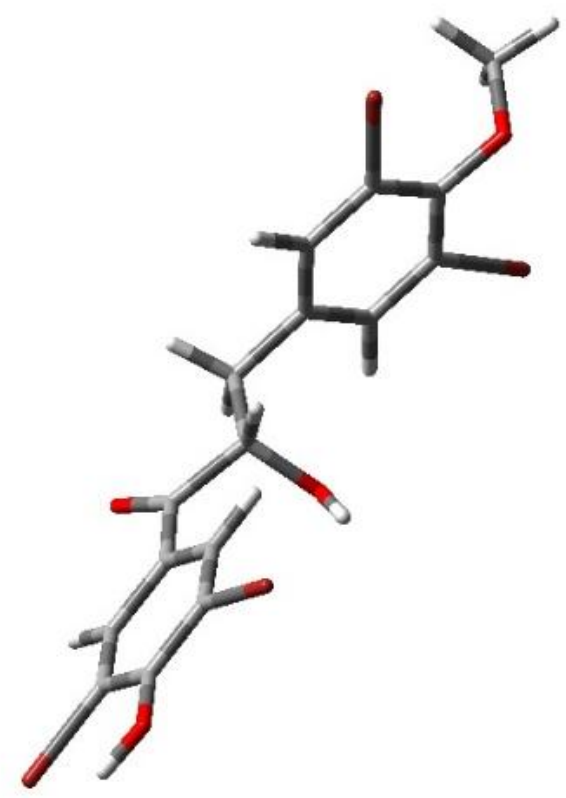

2*S*-1b-Conf-1 (84.93%)

Table S12. Boltzmann populations relative, to the conformer of the minimum energy, of the conformers of **1a** and **1b** (298.1 K) determined by DFT calculations (PBE0/Def2TZVP).

Conformers of **1a**	Boltzmann population (%)	Conformers of **1b**	Boltzmann population (%)
Conf-1	0.51	*Conf-1*	84.93
Conf-4	0.32	*Conf-3*	8.24
Conf-7	55.67	*Conf-4*	3.42
Conf-9	0.28	*Conf-17*	3.41
Conf-16	1.95		
Conf-25	10.83		
Conf-31	25.49		
Conf-41	4.94		

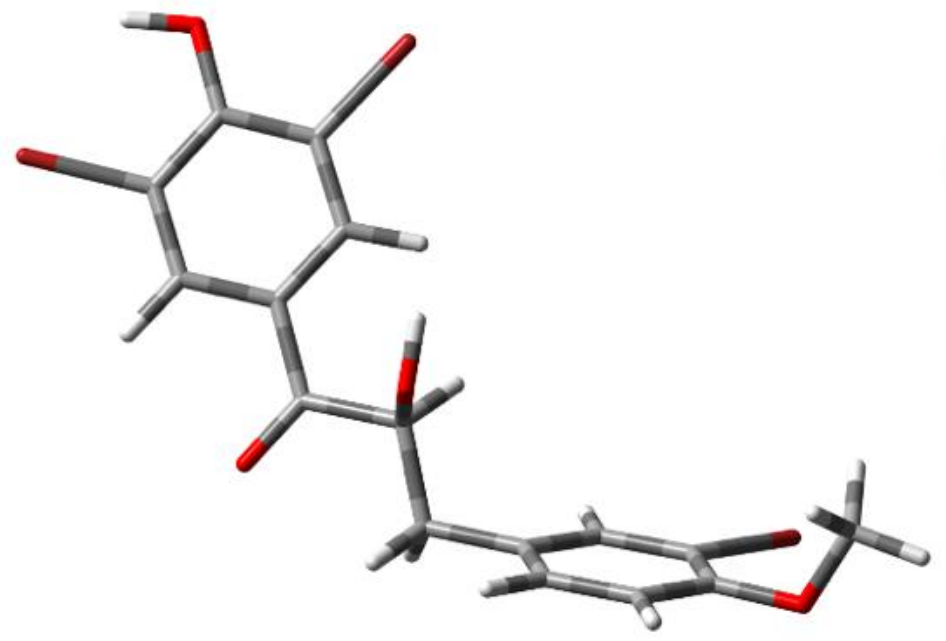

Conformer 1 (0.51 %)

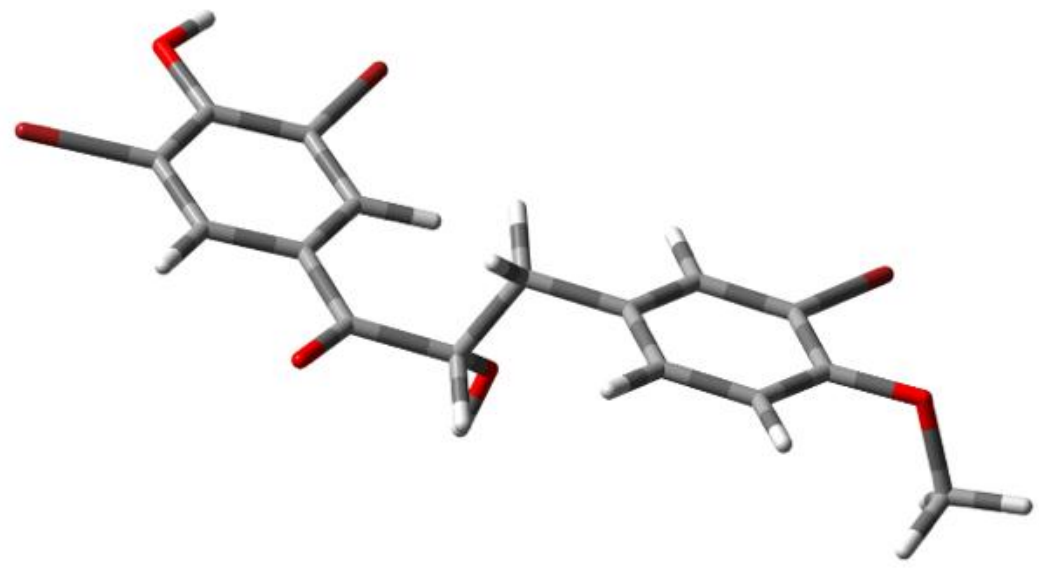

Conformer 4 (0.32 %)

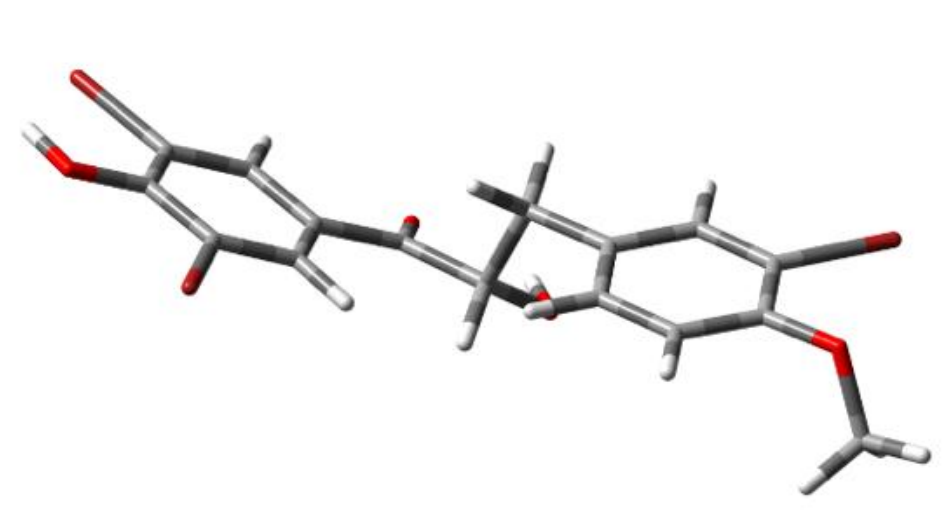

Conformer 7 (55.67 %)

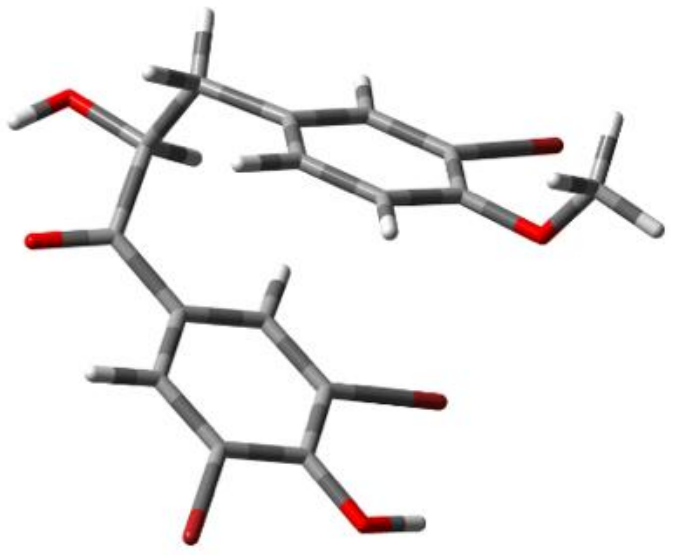

Conformer 9 (0.28 %)

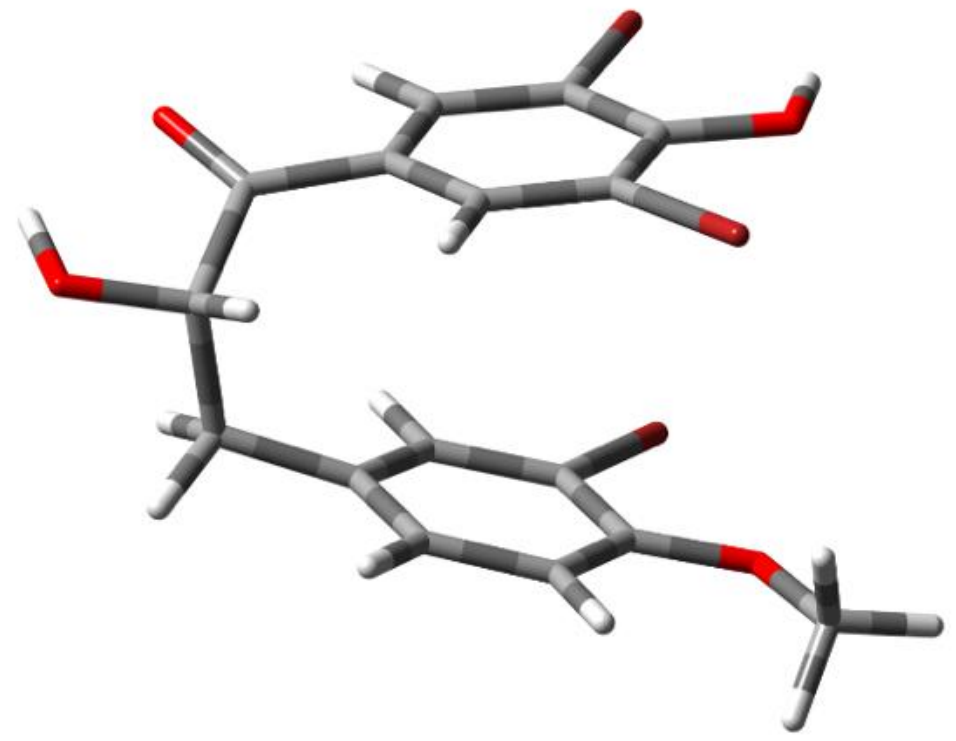

Conformer 16 (1.95 %)

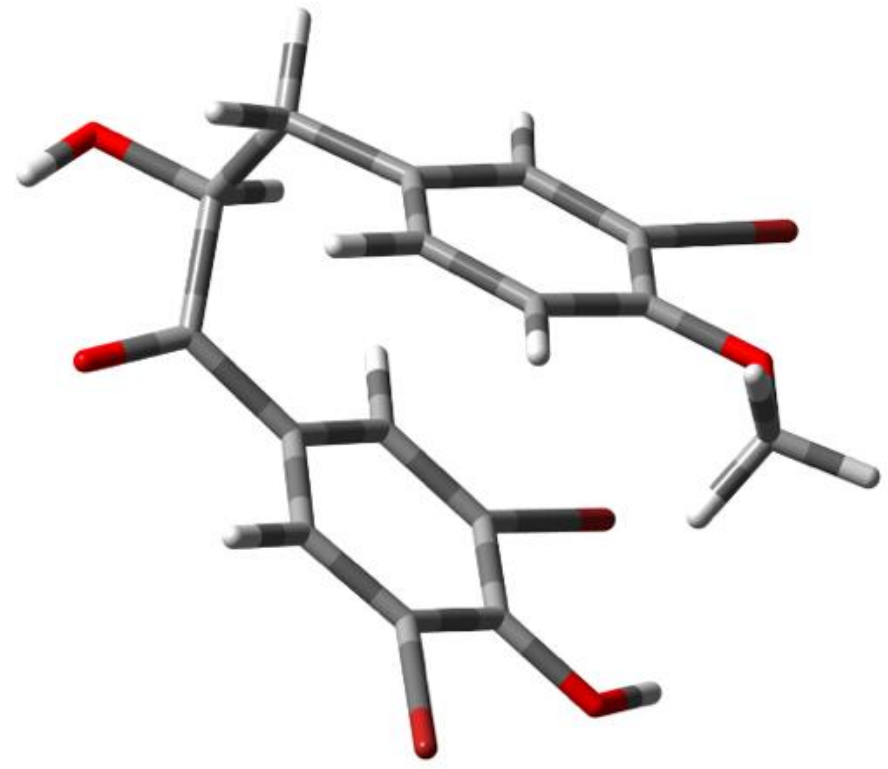

Conformer 25 (10.83 %)

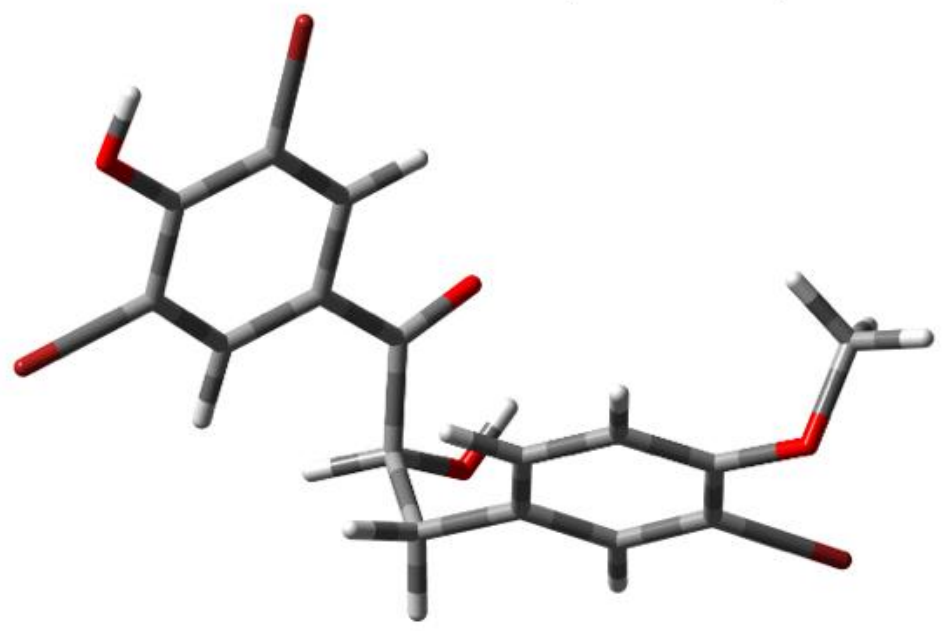

Conformer 31 (25.49 %)

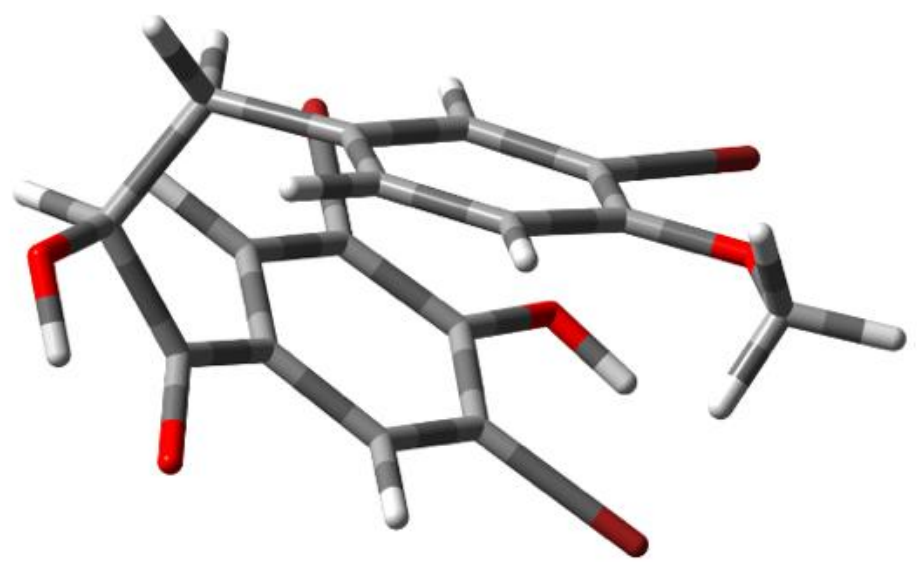

Conformer 41 (4.94 %)

Figure S43. Geometries of the DFT optimized conformers of **1a** (Boltzmann population based on DFT calculations).

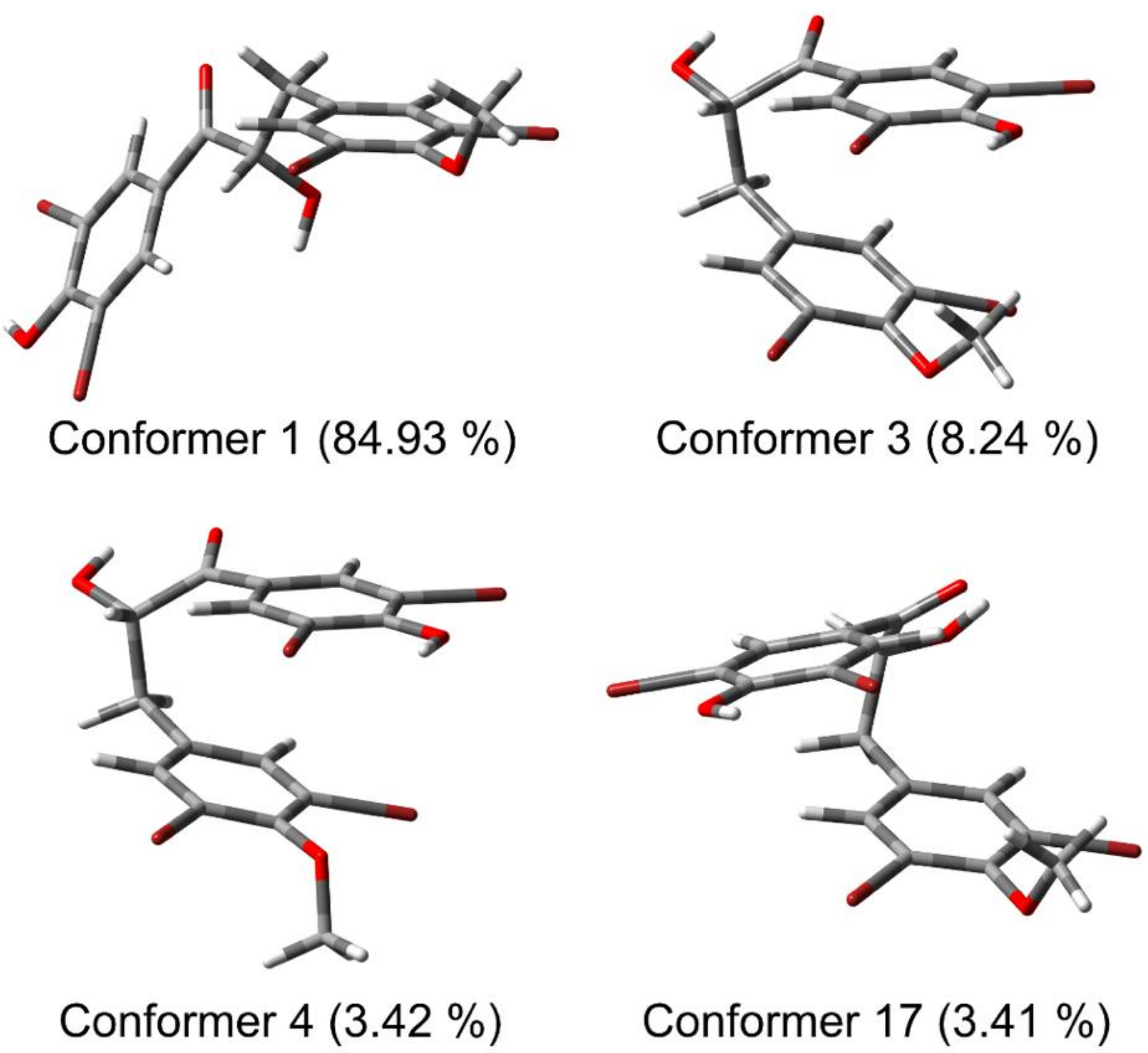

Figure S44. Geometries of the DFT optimized conformers of **1b** (Boltzmann population based on DFT calculations).

Additional ECD calculations of 1a/*ent*-1a

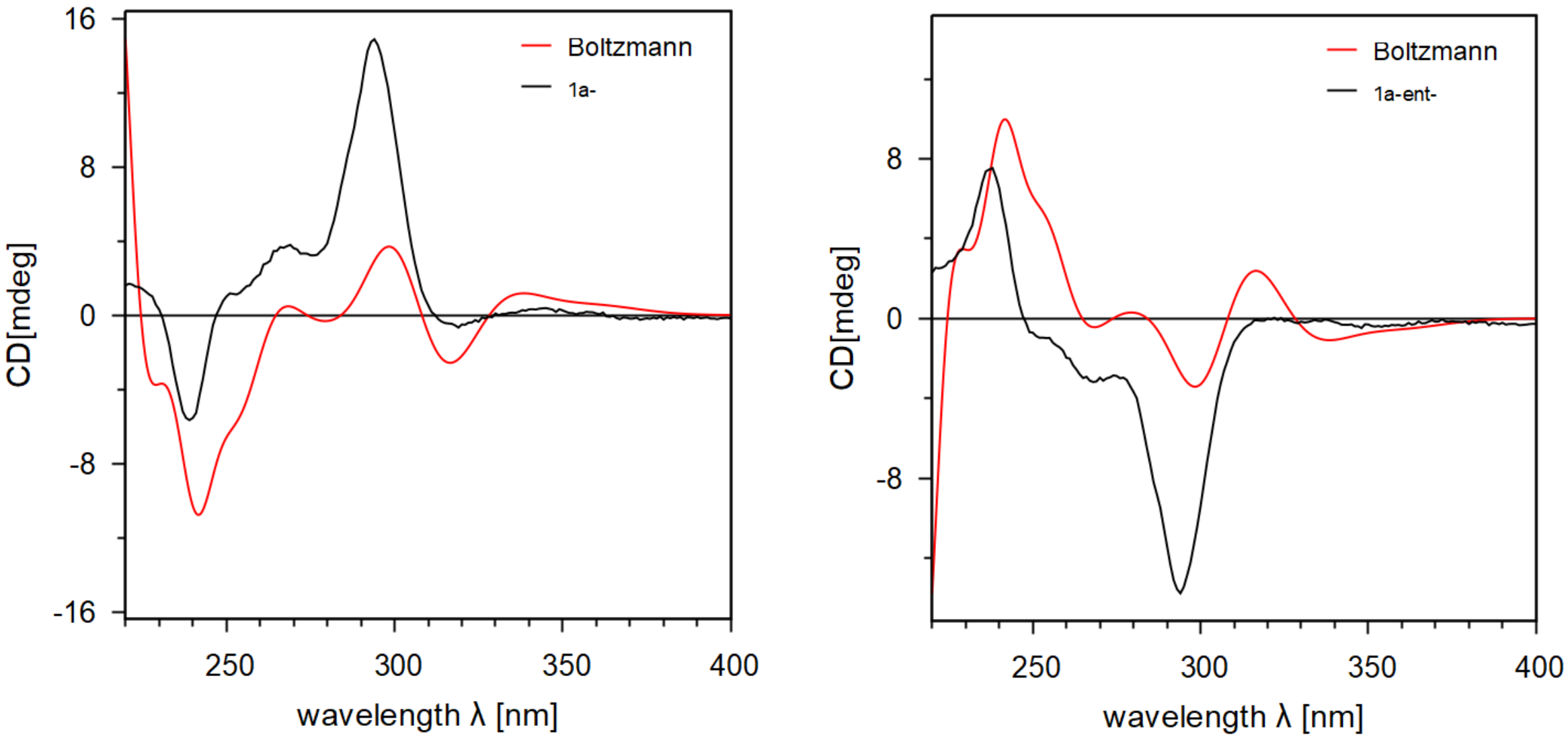

Figure S45. ECD fitting of **1a** (left) and *ent*-**1a** (right). Experimental ECD appears in black, and DFT-computed Boltzmann averaged is shown in red, solvent modelled by COSMO (MeOH), including the lowest 160 singlet excited states. ECD was computed at PBE0/Def2TZVPP/W06. Fitting parameters: Sigma/gamma (eV): 0.19; UV Correction 24 nm; Wavelength (nm) 220–400.

Additional ECD calculations of 1b/*ent*-1b

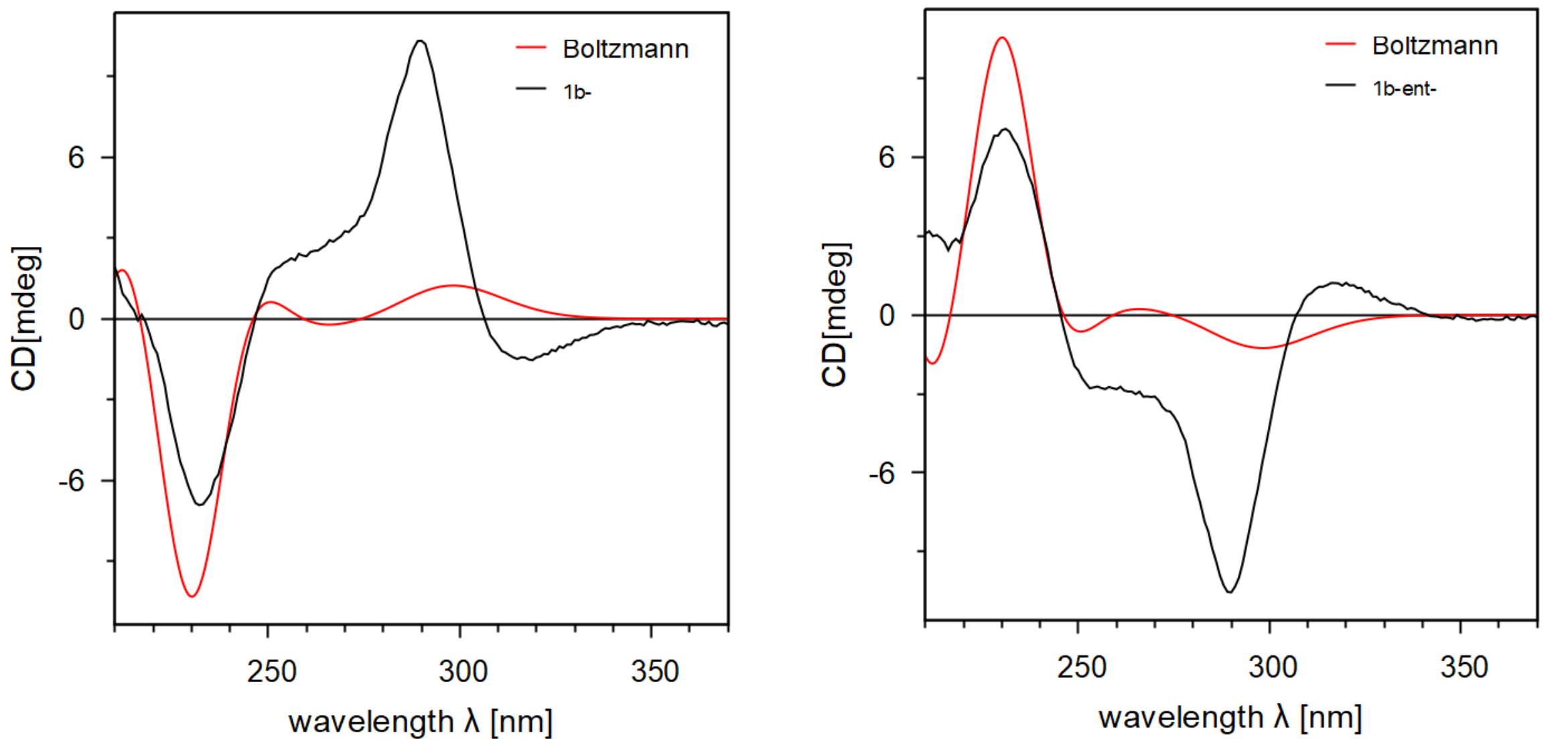

Figure S46. ECD fitting of **1b** (left) and *ent*-**1b** (right). Experimental ECD appears in black, and DFT-computed Boltzmann averaged is shown in red, solvent modelled by IEFPCM (MeOH), including the lowest 260 singlet excited states. ECD was computed at WB97XD/x2c-TZVPall-2c/auto. Fitting parameters: Sigma/gamma (eV): 0.22; UV Correction −20 nm; Wavelength (nm) 210–370.

9 Biological evaluation

9.1 Anti-prion testing

Procerone compound preparation

The procerone compounds employed in this study were: procerone A **1a**, *ent*-**1a**, procerone B **1b**, and *ent*-**1b**. Procerone A **1a** and *ent*-**1a** have a molecular weight of 509 g/mol, while procerone B **1b** and *ent*-**1b** have a molecular weight of 588 g/mol. Each compound was prepared as a 2 mM stock solution in DMSO (Sigma-Aldrich) and stored at −20 °C until use.

Protein misfolding shaking amplification (PMSA) assays

PMSA assays were performed according to previously established protocols with specific modifications for prion propagation analysis.[40] Two recombinant protein substrates were employed: mouse L108I prion protein (rec-MoL108I) and bank vole 109I prion protein (rec-Vole109I). Both substrates were prepared supplemented with 0.5% dextran sulfate (Sigma-Aldrich) as cofactor. Previously characterized recombinant protein preparations were used as inocula: Ust02 strain derived from bank vole 109I prion protein (approximately 0.1 mg/mL)[41] and btMI-09 strain derived from mouse L108I prion protein (approximately 0.1 mg/mL)[42], generated according to methods described by Eraña et al. and previous laboratory studies. Assays were performed in 2 mL polypropylene tubes with a substrate volume of 600 µL. Each compound was tested in duplicate at a final concentration of 50 µM. Controls included tubes treated with DMSO alone at the same volume as the compounds. Inocula were added at an initial 1:100 dilution, and two additional serial passages were performed with 1:100 dilutions between passages (R1, R2, and R3). For each passage and substrate, two negative control replicates (no seed) were included.

Proteinase K digestion and electrophoretic analysis

After each passage, 450 µL of each sample were processed by proteinase K (PK) digestion at a concentration of 50 µg/mL for 1 h at 42 °C. Subsequently, samples were precipitated and analyzed by polyacrylamide gel electrophoresis followed by total protein staining to assess the presence of protease-resistant forms, indicative of successful prion propagation.

9.2 GABA$_A$ testing

Functional expression of GABA$_A$ receptors in Xenopus laevis oocytes

We purchased *Xenopus laevis* oocytes grade II from Ecocyte (Germany) and injected them with 50–75 nL of nuclease-free water containing cRNAs encoding for GABA$_A$ receptor alpha1, beta2, and gamma2 subunits (concentrations 100-1000 ng/µL) in a ratio of 1:1:10 to ensure gamma-subunit incorporation using a Nanoject-III.[43–45] Oocytes were then stored overnight at 19 °C in a ND96-buffer (composition [mM]: NaCl 90, KCl 1, $MgCl_2$ 1, $CaCl_2$ 1, HEPES 5, pH adjusted to 7.4 using 1 M NaOH) supplemented with Penicillin G (100 U/L) and Streptomycin (100 µg/mL).

Electrophysiological assessment of GABA$_A$ receptor modulation

After one day of cRNA injection, oocytes were transferred to the recording chamber, and two-microelectrode voltage clamp recordings were performed as previously described in detail.[46] Briefly, solutions containing either GABA, GABA plus the indicated drug, or ND96 as wash buffer were applied by means of a fast perfusion system.[44–46] To elicit I_{GABA}, the recording chamber was perfused with 200 µL of a GABA-containing solution (duration 20 seconds) and functional expression of GABA$_A$ receptors was determined by application of a saturating GABA concentration (1 mM), followed by application of lower GABA concentrations corresponding to approximately 10% of the maximal response (EC10). Oocytes were held at a holding potential of −70 mV using a TURBO TEC-05X amplifier (npi electronic, Tamm, Germany) connected to an Axon Digidata 1440A digitizer (Molecular Devices, Sunnyvale, CA, USA). Data acquisition was performed using pCLAMP software version 10.2 (Molecular Devices), and current traces were analyzed offline using Clampfit. Microelectrodes were pulled from borosilicate glass (Science Products, Hofheim, Germany), filled with 2 M KCl, and had resistances between 1 and 3 MΩ.

Formal and statistical analysis

Percent modulation of I_{GABA} was calculated using the formula:

$$\frac{(\text{amplitude}_{IGABA-\text{drug}} - \text{amplitude}_{\text{IGABA}-\text{control}})}{\text{amplitude}_{IGABA-\text{control}}} \cdot 100$$

Data are presented as mean ± S.E.M from 5–8 independent measurements for each data point and drawn from individual cells. We run two-tailed, *one-sample t-tests* to determine significant drug effects with *P*-values < 0.05 accepted as statistically significant using GraphPad Prism version 10.4.1 (La Jolla, USA).

Drugs

All chemicals used for the electrophysiological characterization were obtained from Sigma Aldrich (Vienna, Austria). We prepared 30 mM stock solutions of all compounds in DMSO, and test solutions were freshly prepared immediately before the experiment. Equal amounts of DMSO were present in control and compound-containing solutions at concentrations known not to affect I_{GABA}.[44–46]

9.3 Antiproliferation testing

Human hepatocyte carcinoma cells (HepG2 cells, ATCC HB-8065) were grown in Iscove´s Dulbecco Medium (IMDM) supplemented with 10% FCS and 1% of Penicillin/Streptomycin at 37 °C in an atmosphere containing 5% CO_2. WST-1 assays (water-soluble tetrazolium assay) were performed to assess the effects of the different compounds on HepG2 cell viability. The CC_{50} values were determined by incubating 2 × 10^4 HepG2 cells per well with serial dilutions (1 µM, 5 µM, 10 µM, 25 µM, 50 µM, and 100 µM) of the corresponding compound in a 96-well plate (Cellstar® 96-well Microplate, flat bottom clear polystyrene wells, Greiner Bio-One). After 48 h of incubation, WST-1 reagent was diluted 1:11 in PBS, and 110 µL was added to each well. After 2 h of incubation at 37 °C and 5% CO_2, cell viability was assessed in a Tecan microplate reader (Tecan Infinite M Plex). Absorbance values were normalized to the corresponding DMSO control. CC_{50} values were calculated using non-linear regression analysis on GraphPad Prism version 11 for Windows (GraphPad Software, Boston, Massachusetts USA; www.graphpad.com). Results were based on three independent experiments (n ≥ 3).

Table S13. WST-1 assay results of the synthesized compounds.

Compound	CC_{50} [µM]
Chalcone derivative **12b**	25.6
Chalcone epoxide **13a**	43.6
Chalcone epoxide **13b**	22.3
Hydrogenated derivative **14a**	60.7
Hydrogenated by-product **15a**	50–100
Hydrogenated derivative **14b**	50–100
Hydrogenated by-product **15b**	50–100
Procerone A **1a**	(a)
ent-**1a**	36.9
Procerone B **1b**	23.3
ent-**1b**	26.8

(a) Insufficient activity within the tested concentration range (1–100 µM) to determine the CC_{50} value.

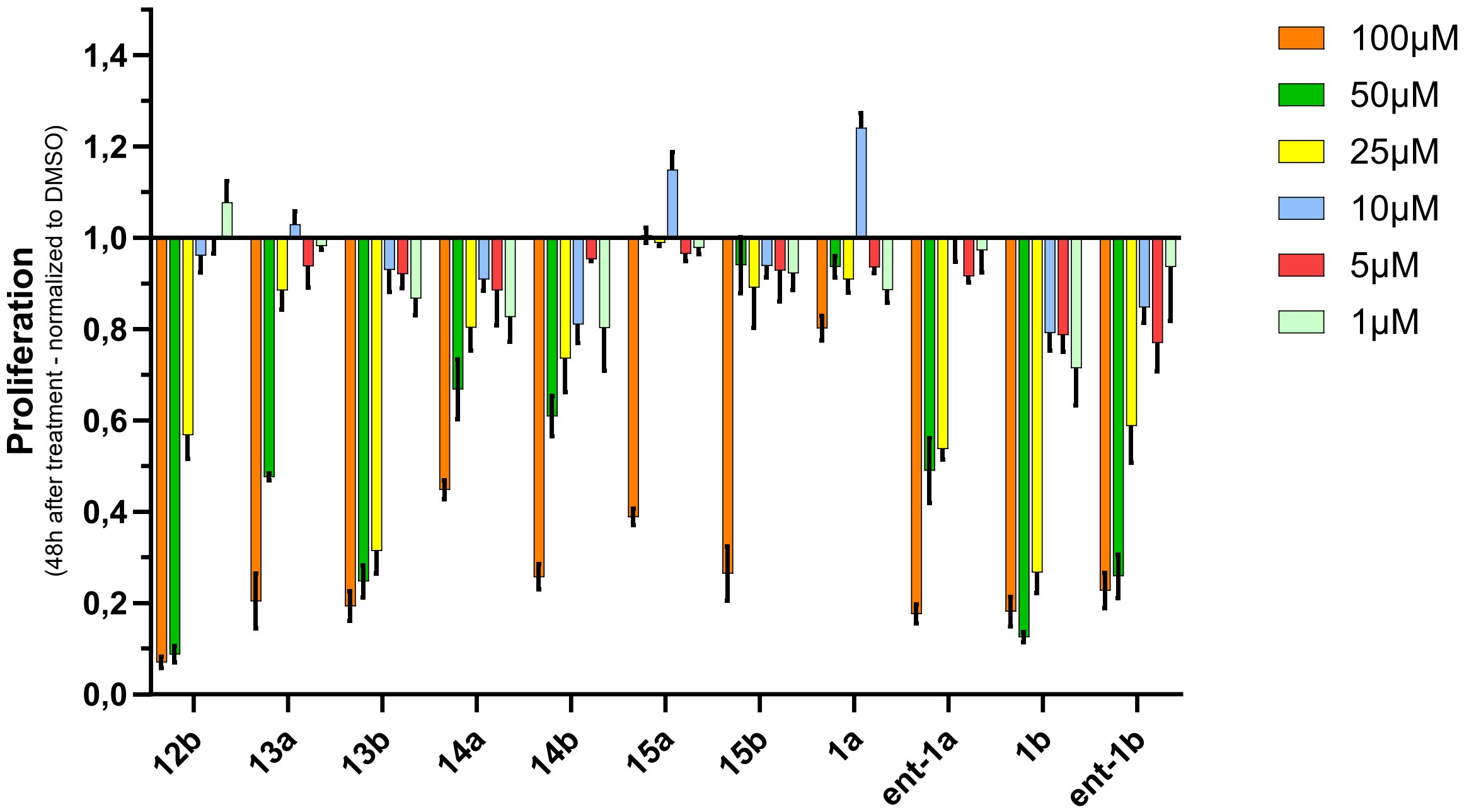

Figure S47. WST-1 assay results of the synthesized precursors **12b**, **13a**, **13b**, **14a**, **14b**, **15a**, **15b** and of procerones A **1a** and B **1b** and their corresponding enantiomers (*ent*-**1a** and *ent*-**1b**) at concentrations of 1 µM, 5 µM, 10 µM, 25 µM, 50 µM, and 100 µM. HepG2 cell viability was assessed 48 h after treatment and normalized to DMSO.

10 References

(1) Fulmer, G. R.; Miller, A. J. M.; Sherden, N. H.; Gottlieb, H. E.; Nudelman, A.; Stoltz, B. M.; Bercaw, J. E.; Goldberg, K. I. NMR Chemical Shifts of Trace Impurities: Common Laboratory Solvents, Organics, and Gases in Deuterated Solvents Relevant to the Organometallic Chemist. *Organometallics* **2010**, *29* (9), 2176–2179. https://doi.org/10.1021/om100106e.

(2) Jennings, L. K.; Robertson, L. P.; Rudolph, K. E.; Munn, A. L.; Carroll, A. R. Anti-Prion Butenolides and Diphenylpropanones from the Australian Ascidian Polycarpa Procera. *J. Nat. Prod.* **2019**, *82* (9), 2620–2626. https://doi.org/10.1021/acs.jnatprod.9b00551.

(3) Mitsui, S.; Senda, Y.; Shimodaira, T.; Ichikawa, H. Studies of Hydrogenolysis. XLIX. The Selective Hydrogenolysis of α,β-Epoxyketones. *Bull. Chem. Soc. Jpn.* **1965**, *38* (11), 1897–1900. https://doi.org/10.1246/bcsj.38.1897.

(4) Augustyn, J. A. N.; Bezuidenhoudt, B. C. B.; Swanepoel, A.; Ferreira, D. Enantioselective Synthesis of Flavonoids. Part 2. Poly-Oxygenated α-Hydroxydihydrochalcones and Circular Dichroic Assessment of Their Absolute Configuration. *Tetrahedron* **1990**, *46* (12), 4429–4442. https://doi.org/10.1016/S0040-4020(01)86778-8.

(5) Hu, Y.; Uno, M.; Harada, A.; Takahashi, S. Selective Ring-Opening Reaction of Epoxides with Sodium Borohydride in the Presence of Cyclodextrins in Aqueous Media. *Chem. Lett.* **1990**, *19* (5), 797–798. https://doi.org/10.1246/CL.1990.797.

(6) Hutchins, R. O.; Cistone, F. Utility and Applications of Borane Dimethylsulfide in Organic Synthesis. A Review. *Org. Prep. Proced. Int.* **1981**, *13* (3–4), 225–240. https://doi.org/10.1080/00304948109356130.

(7) Cossy, J.; Bouzide, A.; Ibhi, S.; Aclinou, P. Formation of β-Hydroxyketones from α,β-Epoxyketones by Photoinduced Single Electron Transfer Reactions. *Tetrahedron* **1991**, *47* (37), 7775–7782. https://doi.org/10.1016/S0040-4020(01)81935-9.

(8) Marques, C. S.; Moura, N.; Burke, A. J. A Simple, Highly Regioselective, One-Pot Stereoselective Synthesis of Tertiary α-Hydroxyesters: A Tandem Oxidation/Benzilic Ester Rearrangement. *Tetrahedron Lett.* **2006**, *47* (34), 6049–6052. https://doi.org/10.1016/j.tetlet.2006.06.107.

(9) Kabsch, W. XDS. *Acta Cryst.* **2010**, *D66* (2), 125–132. https://doi.org/10.1107/S0907444909047337.

(10) Krause, L.; Herbst-Irmer, R.; Sheldrick, G. M.; Stalke, D. Comparison of Silver and Molybdenum Microfocus X-Ray Sources for Single-Crystal Structure Determination. *J. Appl. Crystallogr.* **2015**, *48* (1), 3–10. https://doi.org/10.1107/S1600576714022985.

(11) Sheldrick, G. M. SHELXT – Integrated Space-Group and Crystal-Structure Determination. *Acta Cryst.* **2015**, *A71*, 3–8. https://doi.org/10.1107/S2053273314026370.

(12) Sheldrick, G. M. Crystal Structure Refinement with SHELXL. *Acta Cryst.* **2015**, *C71*, 3–8. https://doi.org/10.1107/S2053229614024218.

(13) Hübschle, C. B.; Sheldrick, G. M.; Dittrich, B. ShelXle : A Qt Graphical User Interface for SHELXL. *J. Appl. Cryst.* **2011**, *44* (6), 1281–1284. https://doi.org/10.1107/S0021889811043202.

(14) Farrugia, L. J. WinGX and ORTEP for Windows: An Update. *J. Appl. Crystallogr.* **2012**, *45* (4), 849–854. https://doi.org/10.1107/S0021889812029111.

(15) Noguez, C.; Hidalgo, F. Ab Initio Electronic Circular Dichroism of Fullerenes, Single-Walled Carbon Nanotubes, and Ligand-Protected Metal Nanoparticles. *Chirality* **2014**, *26* (9), 553–562. https://doi.org/10.1002/chir.22348.

(16) Pescitelli, G.; Bruhn, T. Good Computational Practice in the Assignment of Absolute Configurations by TDDFT Calculations of ECD Spectra. *Chirality* **2016**, *28* (6), 466–474. https://doi.org/10.1002/chir.22600.

(17) Gille, A. L.; Dutmer, B. C.; Gilbert, T. M. PCMODEL 9.2. *J. Am. Chem. Soc.* **2009**, *131* (15), 5714–5714. https://doi.org/10.1021/ja9022144.

(18) Tobiason, F. L.; Vergoten, G. GMMX Conformation Searching and Prediction of NMR Proton-Proton Coupling Constants. In *Biomolecular Structure and Dynamics*; Springer Netherlands: Dordrecht, 1997; pp 179–186. https://doi.org/10.1007/978-94-011-5484-0_8.

(19) Halgren, T. A. MMFF VI. MMFF94s Option for Energy Minimization Studies. *J. Comput. Chem.* **1999**, *20* (7), 720–729. https://doi.org/10.1002/(SICI)1096-987X(199905)20:7<720::AID-JCC7>3.0.CO;2-X.

(20) Halgren, T. A. Merck Molecular Force Field. I. Basis, Form, Scope, Parameterization, and Performance of MMFF94. *J. Comput. Chem.* **1996**, *17* (5–6), 490–519. https://doi.org/10.1002/(SICI)1096-987X(199604)17:5/6<490::AID-JCC1>3.0.CO;2-P.

(21) Frisch, M. J.; Trucks, G. W.; Schlegel, H. B.; Scuseria, G. E.; Robb, M. A.; Cheeseman, J. R.; Scalmani, G.; Barone, V.; Mennucci, B.; Petersson, G. A.; Nakatsuji, H.; Caricato, M.; Li, X.; Hratchian, H. P.; Izmaylov, A. F.; Bloino, J.; Zheng, G.; Sonnenberg, J. L.; Hada, M.; Ehara, M.; Toyota, K.; Fukuda, R.; Hasegawa, J.; Ishida, M.; Nakajima, T.; Honda, Y.; Kitao, O.; Nakai, H.; Vreven, T.; Montgomery, J. J. A.; Peralta, J. E.; Ogliaro, F.; Bearpark, M.; Heyd, J. J.; Brothers, E.; Kudin, K. N.; Staroverov, V. N.; Kobayashi, R.; Normand, J.; Raghavachari, K.; Rendell, A.; Burant, J. C.; Iyengar, S. S.; Tomasi, J.; Cossi, M.; Rega, N.; Millam, J. M.; Klene, M.; Knox, J. E.; Cross, J. B.; Bakken, V.; Adamo, C.; Jaramillo, J.; Gomperts, R.; Stratmann, R. E.; Yazyev, O.; Austin, A. J.; Cammi, R.; Pomelli, C.; Ochterski, J. W.; Martin, R. L.; Morokuma, K.; Zakrzewski, V. G.; Voth, G. A.; Salvador, P.; Dannenberg, J. J.; Dapprich, S.; Daniels, A. D.; Farkas, Ö.; Foresman, J. B.; Ortiz, J. V.; Cioslowski, J.; Fox, D. J. *Gaussian 09, Revision B.01, Gaussian, Inc., Wallingford CT, Wallingford CT*; 2009.

(22) Adamo, C.; Barone, V. Exchange Functionals with Improved Long-Range Behavior and Adiabatic Connection Methods without Adjustable Parameters: The MPW and MPW1PW Models. *J. Chem. Phys.* **1998**, *108* (2), 664–675. https://doi.org/10.1063/1.475428.

(23) Scalmani, G.; Frisch, M. J. Continuous Surface Charge Polarizable Continuum Models of Solvation. I. General Formalism. *J. Chem. Phys.* **2010**, *132* (11), 114110. https://doi.org/10.1063/1.3359469.

(24) Adamo, C.; Barone, V. Toward Reliable Density Functional Methods without Adjustable Parameters: The PBE0 Model. *J. Chem. Phys.* **1999**, *110* (13), 6158–6170. https://doi.org/10.1063/1.478522.

(25) Weigend, F.; Ahlrichs, R. Balanced Basis Sets of Split Valence, Triple Zeta Valence and Quadruple Zeta Valence Quality for H to Rn: Design and Assessment of Accuracy. *Phys. Chem. Chem. Phys.* **2005**, *7* (18), 3297–3305. https://doi.org/10.1039/b508541a.

(26) Weigend, F. Accurate Coulomb-Fitting Basis Sets for H to Rn. *Phys. Chem. Chem. Phys.* **2006**, *8* (9), 1057–1065. https://doi.org/10.1039/b515623h.

(27) Klamt, A.; Schüürmann, G. COSMO: A New Approach to Dielectric Screening in Solvents with Explicit Expressions for the Screening Energy and Its Gradient. *J. Chem. Soc. Perkin Trans. 2* **1993**, No. 5, 799–805. https://doi.org/10.1039/P29930000799.

(28) Hagberg, A. A.; Schult, D. A.; Swart, P.; Hagberg, J. *Proceedings of the Python in Science Conference*; 2008.

(29) Kabsch, W. A Solution for the Best Rotation to Relate Two Sets of Vectors. *Acta Crystallogr. Sect. A* **1976**, *32* (5), 922–923. https://doi.org/10.1107/S0567739476001873.

(30) Cioslowski, J. Rovibrational States of Wigner Molecules in Spherically Symmetric Confining Potentials. *J. Chem. Phys.* **2016**, *145* (5), 054116. https://doi.org/10.1063/1.4959899.

(31) Fuentes-Monteverde, J. C. C.; Nath, N.; Forero, A. M.; Balboa, E. M.; Navarro-Vázquez, A.; Griesinger, C.; Jiménez, C.; Rodríguez, J. Connection of Isolated Stereoclusters by Combining 13C-RCSA, RDC, and J-Based Configurational Analyses and Structural Revision of a Tetraprenyltoluquinol Chromane Meroterpenoid from Sargassum Muticum. *Mar. Drugs* **2022**, *20* (7), 462. https://doi.org/10.3390/md20070462.

(32) Zhao, Y.; Truhlar, D. G. Design of Density Functionals That Are Broadly Accurate for Thermochemistry, Thermochemical Kinetics, and Nonbonded Interactions. *J. Phys. Chem. A* **2005**, *109* (25), 5656–5667. https://doi.org/10.1021/jp050536c.

(33) Gulde, R.; Pollak, P.; Weigend, F. Error-Balanced Segmented Contracted Basis Sets of Double-ζ to Quadruple-ζ Valence Quality for the Lanthanides. *J. Chem. Theory Comput.* **2012**, *8* (11), 4062–4068. https://doi.org/10.1021/ct300302u.

(34) Yanai, T.; Tew, D. P.; Handy, N. C. A New Hybrid Exchange–Correlation Functional Using the Coulomb-Attenuating Method (CAM-B3LYP). *Chem. Phys. Lett.* **2004**, *393* (1–3), 51–57. https://doi.org/10.1016/j.cplett.2004.06.011.

(35) Pollak, P.; Weigend, F. Segmented Contracted Error-Consistent Basis Sets of Double- and Triple-ζ Valence Quality for One- and Two-Component Relativistic All-Electron Calculations. *J. Chem. Theory Comput.* **2017**, *13* (8), 3696–3705. https://doi.org/10.1021/acs.jctc.7b00593.

(36) Chai, J.-D.; Head-Gordon, M. Long-Range Corrected Hybrid Density Functionals with Damped Atom–Atom Dispersion Corrections. *Phys. Chem. Chem. Phys.* **2008**, *10* (44), 6615. https://doi.org/10.1039/b810189b.

(37) Pritchard, B. P.; Altarawy, D.; Didier, B.; Gibson, T. D.; Windus, T. L. New Basis Set Exchange: An Open, Up-to-Date Resource for the Molecular Sciences Community. *J. Chem. Inf. Model.* **2019**, *59* (11), 4814–4820. https://doi.org/10.1021/acs.jcim.9b00725.

(38) Schuchardt, K. L.; Didier, B. T.; Elsethagen, T.; Sun, L.; Gurumoorthi, V.; Chase, J.; Li, J.; Windus, T. L. Basis Set Exchange: A Community Database for Computational Sciences. *J. Chem. Inf. Model.* **2007**, *47* (3), 1045–1052. https://doi.org/10.1021/ci600510j.

(39) Bruhn, T.; Schaumlöffel, A.; Hemberger, Y.; Bringmann, G. SpecDis: Quantifying the Comparison of Calculated and Experimental Electronic Circular Dichroism Spectra. *Chirality* **2013**, *25* (4), 243–249. https://doi.org/10.1002/chir.22138.

(40) Eraña, H.; Díaz-Domínguez, C. M.; Charco, J. M.; Vidal, E.; González-Miranda, E.; Pérez-Castro, M. A.; Piñeiro, P.; López-Moreno, R.; Sampedro-Torres-Quevedo, C.; Fernández-Veiga, L.; Tasis-Galarza, J.; Lorenzo, N. L.; Santini-Santiago, A.; Lázaro, M.; García-Martínez, S.; Gonçalves-Anjo, N.; San-Juan-Ansoleaga, M.; Galarza-Ahumada, J.; Fernández-Muñoz, E.; Giler, S.; Valle, M.; Telling, G. C.; Geijó, M.; Requena, J. R.; Castilla, J. Understanding the Key Features of the Spontaneous Formation of Bona Fide Prions through a Novel Methodology That Enables Their Swift and Consistent Generation. *Acta Neuropathol. Commun.* **2023**, *11* (1), 1–30. https://doi.org/10.1186/s40478-023-01640-8.

(41) Eraña, H.; Sampedro-Torres-Quevedo, C.; Charco, J. M.; Díaz-Domínguez, C. M.; Peccati, F.; San-Juan-Ansoleaga, M.; Vidal, E.; Gonçalves-Anjo, N.; Pérez-Castro, M. A.; González-Miranda, E.; Piñeiro, P.; Fernández-Veiga, L.; Galarza-Ahumada, J.; Fernández-Muñoz, E.; Perez de Nanclares, G.; Telling, G.; Geijo, M.; Jiménez-Osés, G.; Castilla, J. A Protein Misfolding Shaking Amplification-Based Method for the Spontaneous Generation of Hundreds of Bona Fide Prions. *Nat. Commun.* **2024**, *15* (1), 1–14. https://doi.org/10.1038/s41467-024-46360-2.

(42) Pérez-Castro, M. Á.; Eraña, H.; Vidal, E.; Charco, J. M.; Lorenzo, N. L.; Gonçalves-Anjo, N.; Galarza-Ahumada, J.; Díaz-Domínguez, C. M.; Piñeiro, P.; González-Miranda, E.; Giler, S.; Telling, G.; Sánchez-Martín, M. A.; Garrido, J.; Geijo, M.; Requena, J. R.; Castilla, J. Cofactors Facilitate Bona Fide Prion Misfolding in Vitro but Are Not Necessary for the Infectivity of Recombinant Murine Prions. *PLOS Pathog.* **2025**, *21* (1), e1012890. https://doi.org/10.1371/journal.ppat.1012890.

(43) Strommer, B.; Khom, S.; Kastenberger, I.; Cicek, S. S.; Stuppner, H.; Schwarzer, C.; Hering, S. A Cycloartane Glycoside Derived from Actaea Racemosa L. Modulates GABAA Receptors and Induces Pronounced Sedation in Mice. *J. Pharmacol. Exp. Ther.* **2014**, *351* (2), 234–242. https://doi.org/10.1124/jpet.114.218024.

(44) Hintersteiner, J.; Haider, M.; Luger, D.; Schwarzer, C.; Reznicek, G.; Jäger, W.; Khom, S.; Mihovilovic, M. D.; Hering, S. Esters of Valerenic Acid as Potential Prodrugs. *Eur. J. Pharmacol.* **2014**, *735* (1), 123–131. https://doi.org/10.1016/j.ejphar.2014.03.019.

(45) Stadler, M.; Monticelli, S.; Seidel, T.; Luger, D.; Salzer, I.; Boehm, S.; Holzer, W.; Schwarzer, C.; Urban, E.; Khom, S.; Langer, T.; Pace, V.; Hering, S. Design, Synthesis, and Pharmacological Evaluation of Novel Β2/3 Subunit-Selective γ-Aminobutyric Acid Type A (GABA A) Receptor Modulators. *J. Med. Chem.* **2019**, *62* (1), 317–341. https://doi.org/10.1021/acs.jmedchem.8b00859.

(46) Khom, S.; Baburin, I.; Timin, E.; Hohaus, A.; Sieghart, W.; Hering, S. Pharmacological Properties of GABAA Receptors Containing Gamma1 Subunits. *Mol. Pharmacol.* **2006**, *69* (2), 640–649. https://doi.org/10.1124/mol.105.017236.
